# Spatially resolved multiomics of human meningeal development reveal lineage and disease dynamics

**DOI:** 10.1038/s41556-026-02075-8

**Published:** 2026-09-28

**Authors:** Ken To, J. Patrick Pett, Olli Dufva, Nana-Jane Chipampe, Krzysztof Polanski, Ana-Maria Cujba, Kenny Roberts, Alexander V. Predeus, Nadav Yayon, Kazumasa Kanemaru, Manal I. Patel, David Horsfall, Tong Li, Pavel Mazin, Batuhan Cakir, Rakesh Kapuge, Alicja Wilk, Fani Memi, Xiaoling He, Nita Solanky, Saeed Kayhanian, Richard Mair, Koen Rademaker, Omer Ali Bayraktar, Muzlifah Haniffa, Roger A. Barker, Christopher D. Buckley, Sarah A. Teichmann

**Affiliations:** 1 Cambridge Stem Cell Institute, Jeffrey Cheah Biomedical Centre, Cambridge Biomedical Campus, University of Cambridge, Cambridge, UK; 2 Wellcome Trust Sanger Institute, Wellcome Genome Campus, Hinxton, UK; 3 Department of Surgery, University of Cambridge, Cambridge, UK; 4 Newcastle University, Biosciences Institute, Newcastle University, Newcastle, UK; 5 John van Geest Centre for Brain Repair, Department of Clinical Neuroscience, Cambridge University, Cambridge, UK; 6 Human Developmental Biology Resource, UCL Great Ormond Street Institute of Child Health, University College London, London, UK; 7 Division of Neurosurgery, Department of Clinical Neuroscience, Cambridge University Hospitals, Cambridge, UK; 8 Cancer Research UK Cambridge Centre, Cambridge Biomedical Campus, University of Cambridge, Cambridge, UK; 9 Department of Dermatology, NIHR Newcastle Biomedical Research Centre, Newcastle Hospitals NHS Foundation Trust, Newcastle, UK; 10 Kennedy Institute of Rheumatology, University of Oxford, Oxford, UK

**Keywords:** Cell lineage, RNA sequencing, Gene regulatory networks

## Abstract

The meninges are increasingly recognized as key stromal and immune niches supporting cranial development. Here we constructed a paired single-nuclei RNA and Assay for Transposase-Accessible Chromatin atlas of human meningeal development from 6 to 21 postconception weeks across cranial regions. By integrating spatial transcriptomics, we resolved cell types across meningeal layers. Our multiomic atlas defines gene regulatory networks underlying blood–brain barrier endothelial identity during development. Within immune compartments, the data support a yolk sac origin for meningeal myeloid lineages, including perivascular macrophages primed for barrier immunity. We present two analytical tools: Nichefinder, for spatial label transfer and proximity analysis, and cell2home, for inferring chemotactic signalling. Using cell2home, we identify interactions potentially driving B cell migration from cranial marrow into prenatal meninges. We studied trisomy 21, and observed reduced fibroblast chemokine expression with enhanced JAK–STAT signalling. Together, this work provides a developmental atlas of the human meninges and defines cellular and regulatory programs shaping early barrier immunity.

## Abstract (web summary)

To, Pett, Dufva et al. perform single-nucleus RNA and ATAC sequencing, as well as spatial transcriptomics, to study human meninges at 6–21 weeks post conception.

## Main

The meninges are connective tissue layers formed by fibroblasts that overlie the central nervous system. In adulthood, the pia, arachnoid and dural meningeal layers act as physical barriers that regulate solute transport, and form interfaces housing immune sentinel populations that defend against circulating antigens. Recent single-cell studies have mapped mouse meninges across developmental and postnatal stages, defining distinct fibroblast populations^1^. Similar studies in adult human dura and arachnoid have resolved the taxonomy of adult meningeal cell states, identifying conserved markers such as *MGP* for dural fibroblasts and *LAMA2* for leptomeningeal layers^2,3^. However, the developmental origins and cellular ontology of the human meningeal fibroblasts remain unclear. Fibroblasts of the meninges are thought to derive from both mesodermal and cranial neural crest progenitors, separated by anatomical boundaries. This lineage distinction may be of clinical importance, as meningiomas, the most common adult brain tumour, are hypothesized to originate from regionally distinct meningeal fibroblasts^4,5^. In adulthood, meningeal immunity is formed by resident myeloid and lymphoid populations. In mice, yolk sac progenitors seed the brain during early development, while intracranial progenitors sustain macrophage and microglial compartments throughout life^6^. Integrated human cell atlases suggest similar yolk sac contributions to microglia (MG)^7,8^, but notably exclude meningeal tissues, leaving the developmental trajectory of human central nervous system (CNS) border-associated macrophages (BAMs) unknown. Atlasing the meninges across the first and second trimester is therefore essential to understand how these critical immune populations emerge.

B cells represent another important axis of meningeal immunity. In adulthood, plasma cells accumulate at the dural venous sinuses, and carry out barrier protection. Experimental studies demonstrate that gut-derived IgA plasma cells can traffic to the meninges in response to infection, linking peripheral immunity to CNS defence^9,10^. These findings highlight the meningeal–brain border as a site of B cell education against CNS antigens. Despite these insights, little is known regarding the initial B lineage colonization of the meninges during development. While second-trimester bone marrow niches and peripheral tissues such as the gut can support B lymphopoiesis, the contribution of fetal cranial bone marrow and the mechanisms by which B cells enter the developing meninges remain unexplored. Defining the ontogeny of meningeal fibroblasts, macrophages and B cells is therefore critical to understanding the developmental basis of CNS barrier immunity, with implications for both homeostasis and disease.

## Results

### Cellular taxonomy of the developing meninges

**Fig. 1:**
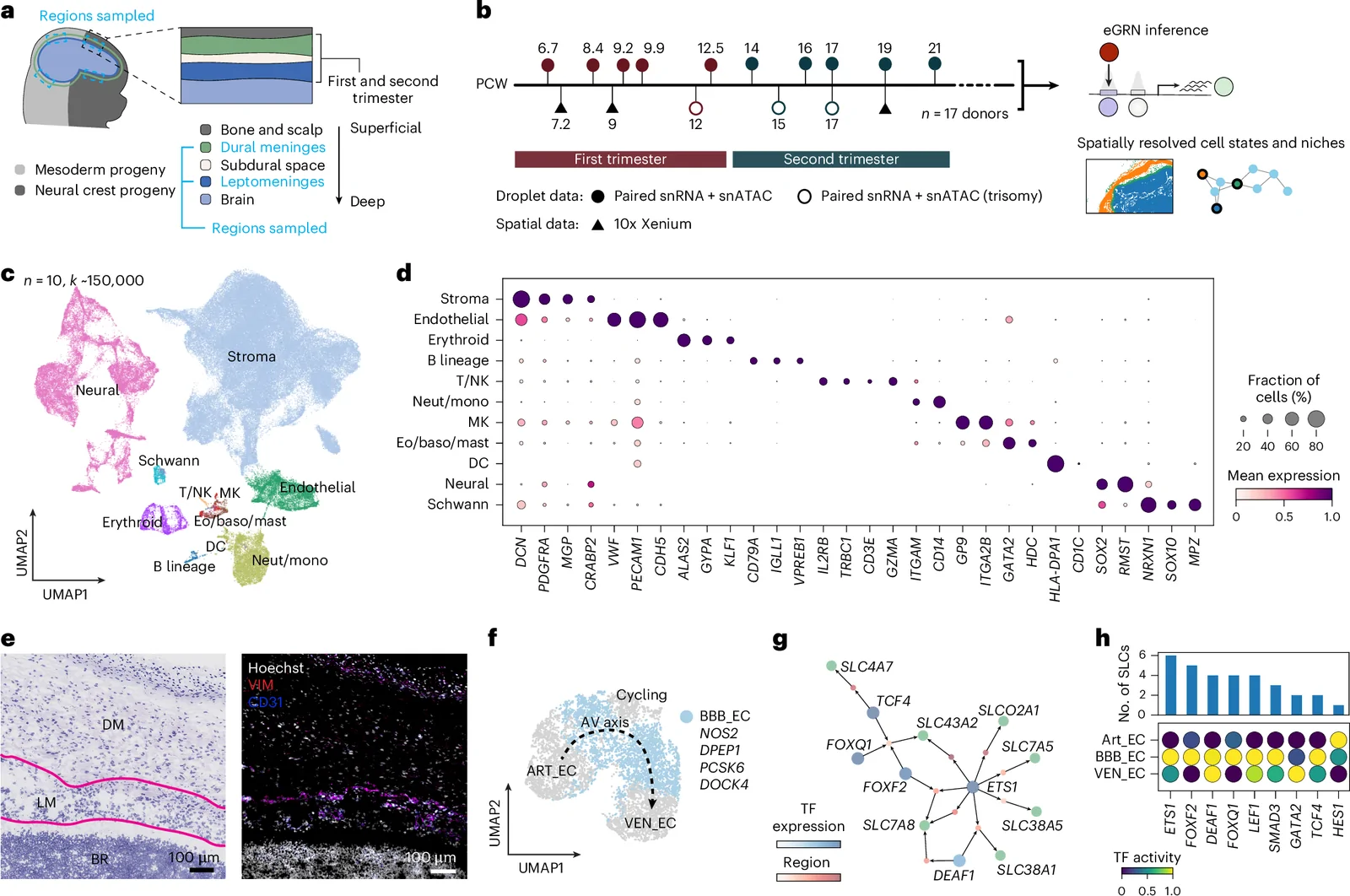
Developmental meninges atlas. **a**, A diagram showing the meningeal regions sampled and a schematic of the layers. **b**, A schematic overview of the samples. The colours indicate the first and second trimester, and the shapes indicate technology applied to sampling. Aneuploidy 21 status of the samples is shown (*n* = 16). Right: analyses undertaken using the multimodal datasets. **c**, A UMAP representation of nuclei droplets (10x Multiome) in the euploid atlas (*n* = 10). **d**, A dot plot showing mean expression of marker genes per cell type. **e**, Left: H&E staining of nine PCW dural and leptomeningeal regions (*n* = 1). The pink line outlines the LM. Right: protein staining (Rarecyte) of CD31 and vimentin, markers of BBB vasculature (*n* = 1). **f**, A UMAP of EC compartment embedding, showing marker genes of BBB_ECs, and the AV axis (dotted arrow). **g**, eGRN of key TFs governing SLC genes in BBB_ECs. **h**, The number of SLC genes directly associated with individual TFs within the BBB_EC eGRN. Baso, basophils; BR, brain region; DC, dendritic cells; DM, dura meninges; Eo, eosinophils; Mast, mast cells; MK, megakaryocytes; Mono, monocytes; NK, natural killer cells; Neut, neutrophils; T, T cells.

**Extended Data Fig. 1:**
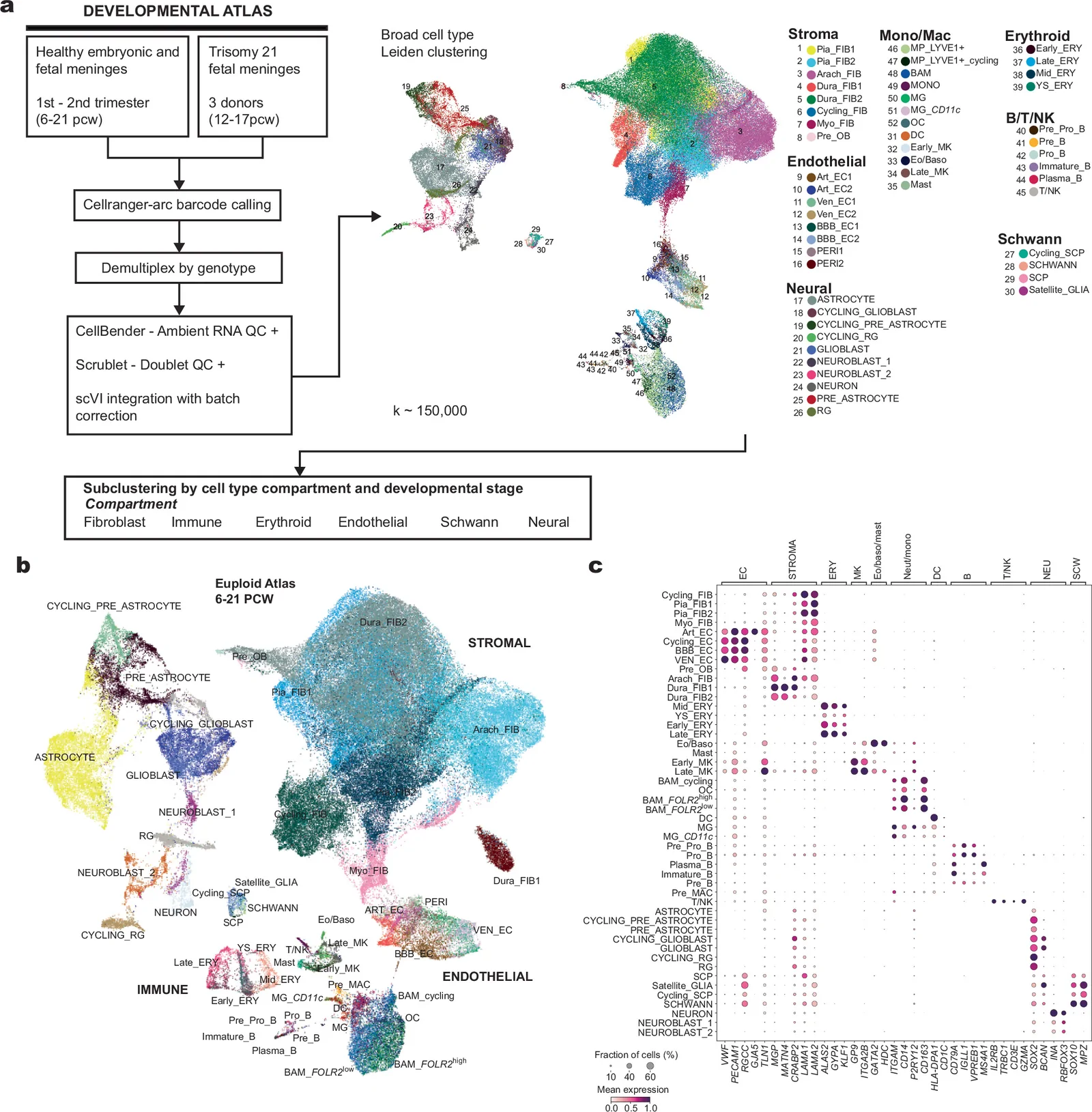
Computational workflow of the developmental meninges atlas. **a**. Trisomy 21 (T21) and Euploid data (n = 13) were integrated, and demultiplexed, followed by QC steps. Each cell type was annotated and subsequently clustered for additional fine-grain annotation. **b**. Global UMAP of the annotated euploid atlas from all developmental stages (n = 10). **c**. Dotplot of marker genes for each cell state.

**Extended Data Fig. 2:**
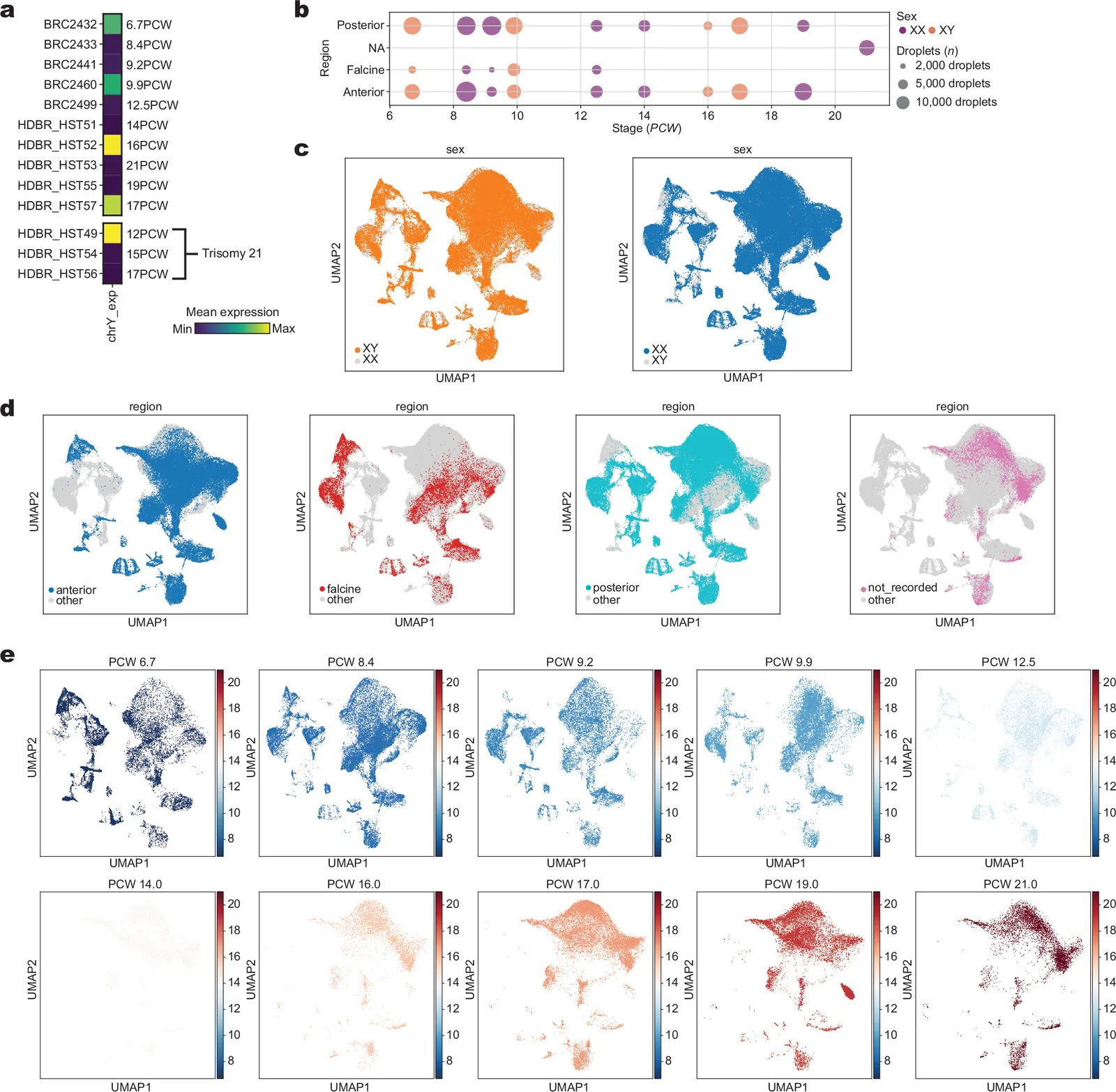
Dataset metadata. **a**. Average expression of Chromosome Y genes within each donor sample. **b**. Dotplot of sex and droplet number metadata for each donor. **c**. UMAP plots of sex metadata. **d**. UMAP plots of region sampling metadata. **e**. UMAP plots of stage (/PCW) metadata.

To curate a transcriptional and epigenetic atlas of the cell states across human meningeal development, we generated paired single-nuclei RNA (snRNA) and single-nuclei Assay for Transposase-Accessible Chromatin (snATAC) sequencing data using (10x Genomics) Multiome, and highly multiplexed (310-plex) cellular-resolution spatial transcriptomics data using a custom panel Xenium (Methods). We performed a study of human embryonic and fetal dural and leptomeningeal layers and sampled from both anterior and posterior regions of the brain relative to the central gyrus (Fig. 1a). Our data comprised tissues from 17 donors, ranging from 6 to 21 postconception weeks (PCW) in developmental age, and included 3 aneuploidy samples with trisomy 21 (T21) (Fig. 1b and Extended Data Fig. 1a–c) and both male and female samples (Extended Data Fig. 2a–e and Supplementary Table 1). We identified 51 transcriptional clusters in our suspension data (*k* ≈ 150,000), comprising predominant fibroblast and endothelial clusters, and a repertoire of immune populations, including B, T, erythroid and myeloid lineages, reflective of cells of the adult meninges, identified by lineage-specific marker genes^11^ (Fig. 1c,d and Extended Data Fig. 1c).

Myeloid and erythroid cell states were differentially enriched in the first trimester, and lymphoid lineage enrichment expanded from the start of the second trimester, concordant with general observations of cross-tissue fetal lymphoid development. All stages of B lineage development were captured, ranging from Pre_Pro_B to Plasma_B clusters (discussed later). This was previously unknown in human development and confirms the findings reported in mouse development^12,13^. Samples in the first trimester also enriched neural populations, suggestive of possible contamination from dissection.

### BBB endothelial development

**Extended Data Fig. 3:**
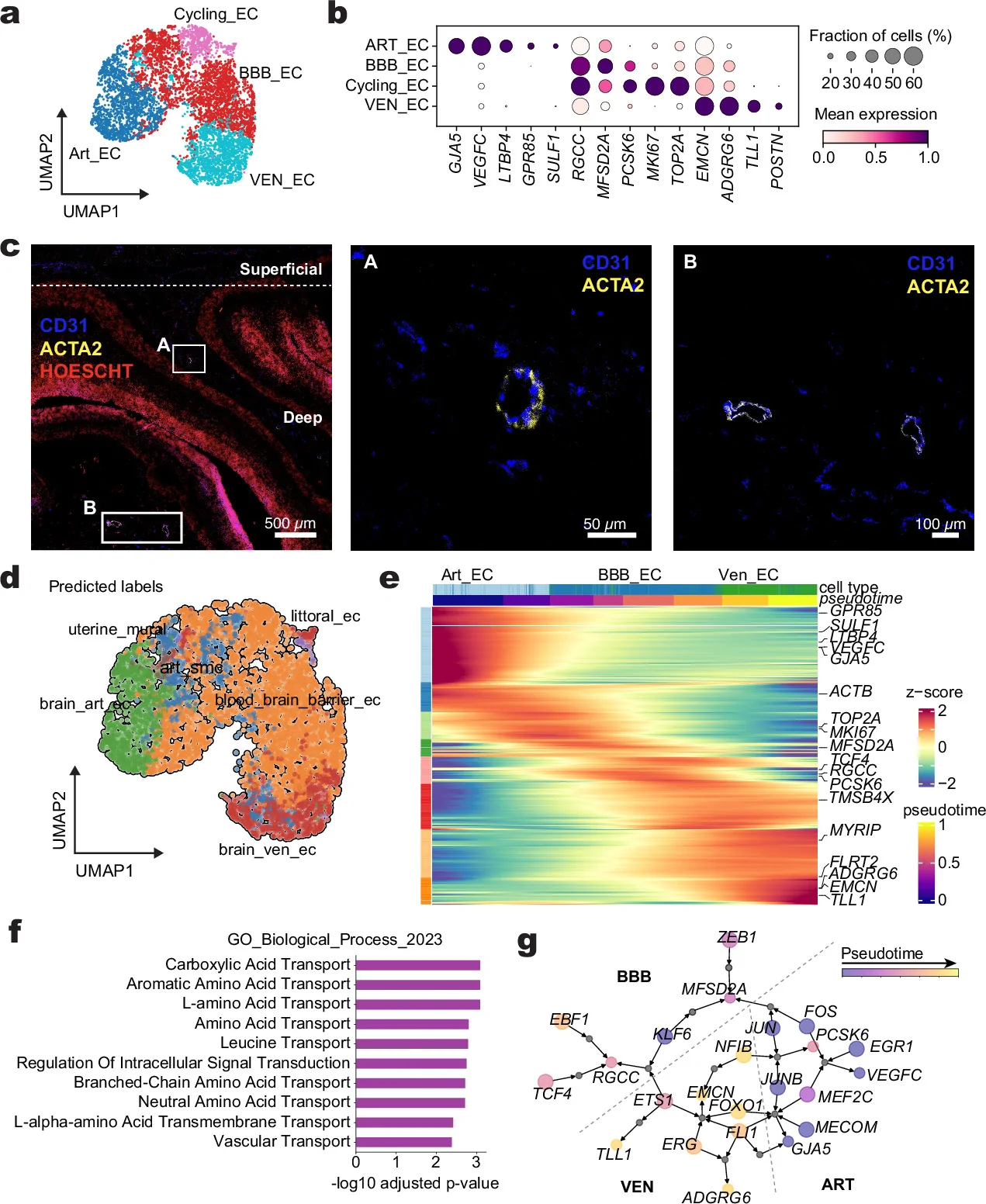
Characterisation of meningeal endothelial cells. **a**. UMAP of annotated cell states (n = 10) and **b**. their maker genes. **c**. Protein staining of endothelial marker genes, demonstrating large diameter arterioles in the parenchyme-associated regions (n = 1). **d**. Celltypist label transfer shown over UMAP of endothelial cells **e**. Monocle3 differential pseudotime associated genes plotted as a heatmap of genes across pseudotime. **f**. Pathway enrichment analysis of BBB_ECs, using the GO geneset. **g**. GRN diagram showing cell-state specific TFs across the endothelial populations.

Our sampling protocol included regions of lepto and dural meninges with resident endothelial cells (ECs) of the BBB (Fig. 1e). This therefore allowed capture of the paired RNA–ATAC profiles of developmental BBB capillary ECs (BBB_ECs). We clustered *CD31* (PECAM)-expressing cells and revealed arterial EC (ART_EC; *GJA5* and *VEGFC*), cycling EC (Cycling_EC; *MKI67* and *TOP2A*) and venous EC (VEN_EC; *EMCN* and *TLL1*) clusters, expressing markers concordant with descriptions in mouse meninges and human adult vasculature atlases^14,15^ (Extended Data Fig. 3a,b). Human cranial endothelium formation is evident at 5 PCW, comparable to mice at embryonic day 11; however, consistent with the absence of lymphatic EC capture in our data, in mice, lymphatic vasculature is reported to colonize the brain and meninges only in the postnatal period, suggesting comparable milestones in human lymphatic development.

We sought to investigate the paucity of *ACTA2*-positive smooth muscle cells within our atlas (Extended Data Fig. 3c) and applied protein staining of ACTA2 and CD31 to identify perivascular smooth muscle cell colocalization with ECs within a 9-PCW sample that included large areas of brain regions. This revealed ACTA2^+^ populations around blood vessels penetrating the brain parenchyma deeply, which were absent within the thin layers of leptomeninges (LM) or the dural meninges, where the embryonic samples were dissected from, suggesting the meninges overlying the brain surface tend to contain small-diameter vessels (Fig. 1e).

Using label transfer via CellTypist (Methods) on an integrated single-cell RNA sequencing atlas of cross-tissue endothelial cells^16,17^, we found concordance at the transcriptional level with prior integrated atlases of brain vasculature (Extended Data Fig. 3d). Embedding of BBB_ECs with ART_ECs and VEN_ECs demonstrated an arteriovenous (AV) axis (Extended Data Fig. 3a,e), previously reported in a single-cell RNA sequencing atlas of brain vasculature^14^. Our EC states recapitulated key transcription factors (TFs) that defined their marker genes (Extended Data Fig. 3b,e) across transitions along the AV axis. For example, *RGCC* enriched for BBB_EC, *GJA5* enriched for ART_EC and *TLL1* enriched for VEN_EC. To gain appreciation for the functional characteristics of BBB_ECs, we applied gene set enrichment analysis and observed enrichment of a number of solute transport functions. This is unsurprising, as the barrier function of BBB_ECs relies on expression of a wealth of genes encoding a vast array of transporters, such as *SLC2A1*, *SLC7A5* and *SLC6A12*, to gate-keep entry to the brain parenchyma^18^. We additionally leveraged the snATAC sequencing data to evaluate TF shifts across pseudotime, which govern the transitions in identity across the AV axis, and identified concordance with marker gene expression for each cell state (Extended Data Fig. 3a,e–g).

To gain an appreciation for aspects of the BBB_EC Gene Regulatory Network (GRN) (Fig. 1f,g) that conferred expression of solute transporters that defined their functional identity, we applied SCENIC+ to predict the key TFs regulating solute transporter genes. We observed *ETS1*, a TF-encoding gene, at the centre of a network that regulates expression of a number of solute carrier (SLC) genes. *SLC7A5*, *SLC7A8* and *SLC43A2*, which all encode amino acid transporters, were directly regulated by *ETS1*. *SLC7A5* has been shown to mediate l-DOPA uptake^19,20^, an important precursor to dopamine synthesis, the neurotransmitter that is deficient in Parkinson’s disease. *ETS1* expression is reported to underpin EC fate^21^, and loss of function results in disruption of BBB function in mice^22,23^. We sought then to determine expression of TFs that regulate solute-transporters across ECs (Fig. 1h), and found strong enrichment for TF activity in BBB_ECs compared with other EC cell states. *FOXF2* and *FOXQ1*, both encoding TFs that regulate BBB development and maintenance, were among the top BBB_EC-specific TFs^24,25^. *DEAF1* was also enriched, and has been linked to numerous neurodevelopmental phenotypes, including neurodevelopmental delay, suggesting a possible link between BBB development and brain development^26^.

### Spatially resolved meningeal mesenchyme development

**Fig. 2:**
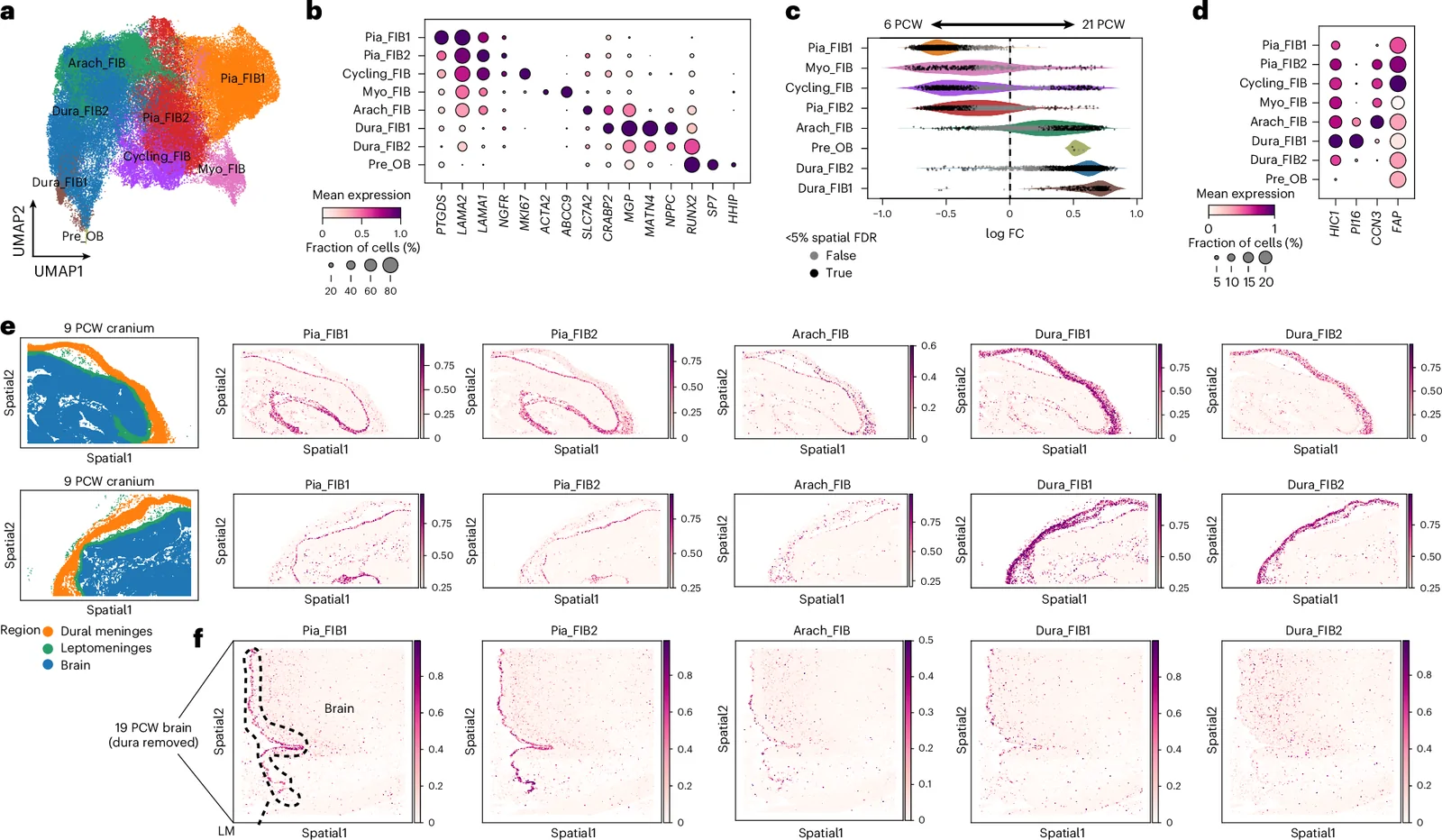
Meningeal mesenchyme development. **a**, A UMAP of mesenchyme clusters in the snRNA sequencing data. **b**, A dot plot of marker genes for each cluster. **c**, A beeswarm plot of MILO results comparing differential neighbourhood PCW enrichment of each cluster. **d**, Developmental fibroblast marker gene expression across the clusters. **e**, Left: spatial plot of segmented droplets from 9 PCW ISS dataset (*n* = 1), with manual annotations of dural and leptomeningeal layers, and the brain. Right: probability of label predictions in segmented droplets within the ISS data. **f**, Left: spatial plot of segmented droplets from a 19-PCW ISS dataset (*n* = 1), with manual annotations of dural and leptomeningeal layers, and the brain. Right: probability of label predictions in segmented droplets within the ISS data. log FC, log fold change.

**Extended Data Fig. 4:**
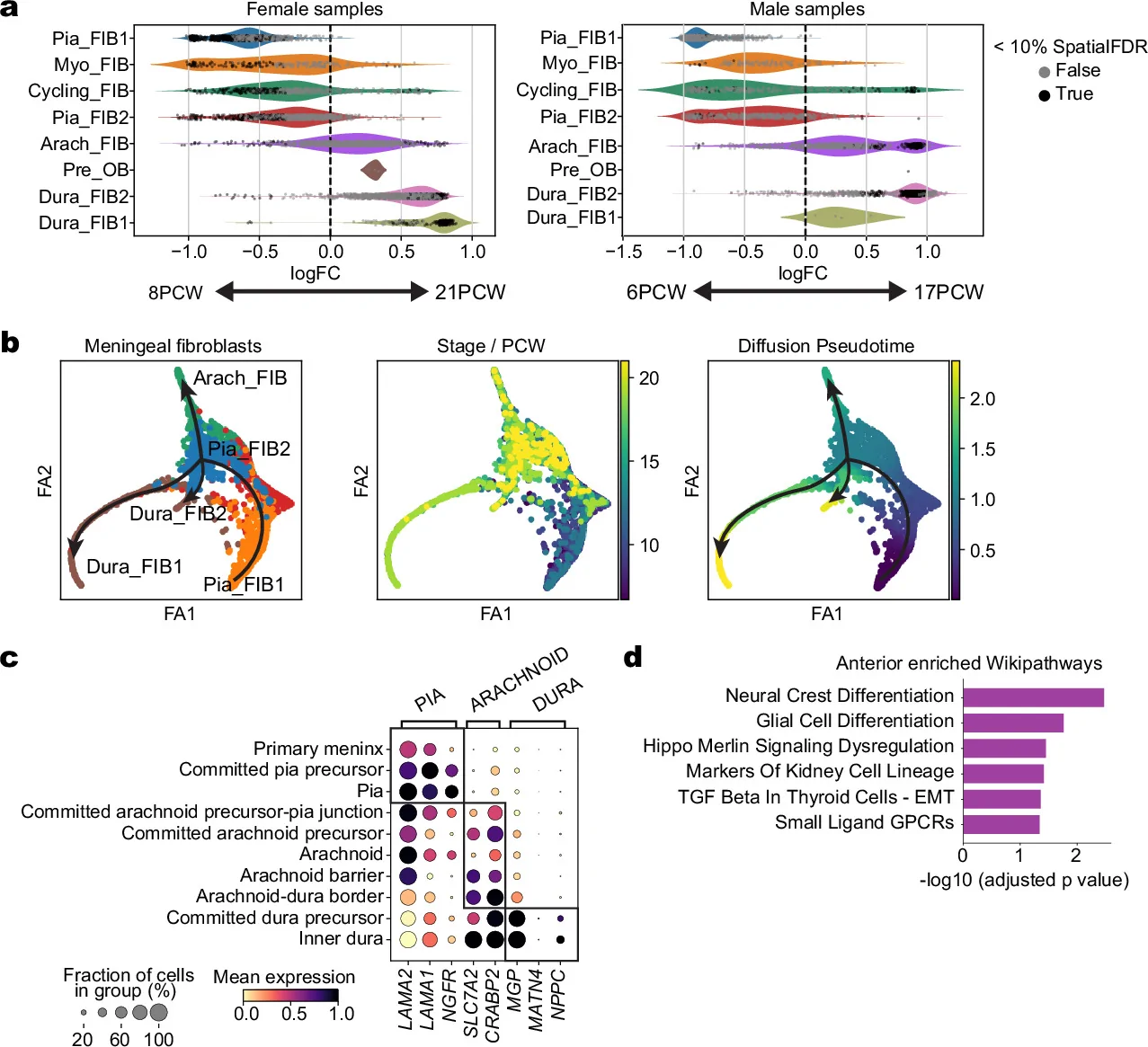
Meningeal mesenchyme development. **a**. Force-directed UMAP of mesenchyme clusters in the snRNAseq data (n = 10), showing cell type labels (left), PCW metadata (middle), Diffusion pseudotime (right). **b**. Functional enrichment of Wikipathway database terms in fibroblasts (grouped) from the anterior region samples. **c**. Dotplot of fibroblast marker gene expression within publicly accessible dataset^27^ of first trimester human meninges development. **d**. Gene-set enrichment analysis of anterior-enriched genes within the Wikipathways geneset (Wilcoxon).

Subclustering of the mesenchyme (*PDGFRA*-enriched; Fig. 2a) compartment revealed seven fine-grained meningeal fibroblast (FIB) cell states, expressing markers consistent with pial (*LAMA2* and *LAMA1*), arachnoid (*SLC7A2* and *CRABP2*) and dural (*MGP*, *MATN4* and *NPPC*) fibroblast populations described in mouse meninges (Fig. 2b). We also detected a minute population of pre-osteoblasts (Pre_OBs), strongly expressing *RUNX2*, encoding a master regulator of osteogenesis, and *HHIP*, which has been described to mark human cranial suture Pre_OBs, possibly related to inclusion of bone tissue adherent to the dura. Using neighbourhood-based differential abundance testing (Methods) across 6–21 PCW, we observed the Pia_FIB and Cycling_FIB clusters to be most enriched at earlier developmental stages, whereas the Arach_FIB and Dura_FIB clusters were significantly enriched at later stages (Fig. 2c). A comparable pattern of enrichment was observed across both male and female samples (Extended Data Fig. 4a and Supplementary Tables 1 and 2). We modelled differentiation pseudotime, which correlated with PCW metadata, and predicted pia cell states as progenitors to arachnoid fibroblasts, and dural fibroblasts, with Pia_FIB1 assigned the earliest pseudotime values (Extended Data Fig. 4b), given its early PCW enrichment. Layer-specific marker genes from our dataset showed concordant expression patterns in an independent first-trimester human meninges dataset^27^, consistent with its layer-specific cell-type annotations (Extended Data Fig. 4c). Although neural crest progenitors were not captured in our dataset, gene set enrichment analysis of combined anterior region-sampled fibroblast populations highlighted neural crest differentiation as the top enriched pathway (Extended Data Fig. 4d and Supplementary Table 3).

We investigated the expression of known developmental fibroblast signatures, which revealed strong enrichment of *HIC1*, a known marker of limb and heart mesenchymal progenitors, across all meningeal fibroblast clusters (Fig. 2d). This aligns with observations in mouse embryos with *HIC1* knockout, which results in lack of dura formation and profound cranial deficits. Among the fibroblasts, Arach_FIB and Dura_FIB1 both distinctly expressed *PI16*, a marker of pan-tissue adventitial fibroblasts, suggesting potential vascular association. Our previous work indicated that *PI16*^+^ embryonic fibroblasts may emerge after *HIC1*^+^ fibroblasts, and this is supported by the differential enrichment of Arach_FIB and Dura_FIB1 in later developmental stages observed here (Fig. 2c). *FAP*, a marker of activated or cancer-associated fibroblasts in adulthood, and *CCN3*, was most enriched in the earliest clusters (Pia_FIB and Cycling_FIB), possibly related to an embryonic phenotype and role in proliferation.

To spatially resolve these fibroblast populations at single-cell resolution, we performed semi-automated selection of cluster-differentiating genes for logistic regression on segmented cells in the Xenium data, which we included as part of our toolkit—Nichefinder (Methods). This enabled precise localization of fibroblast clusters at single-cell resolution into regionally delineated pial, arachnoid and dural layers, concordant with manual annotations of the Xenium dural and leptomeningeal regions (Fig. 2e). At 9 PCW, the outermost dural meningeal region localized Dura_FIB1 and Dura_FIB2 clusters, separate from Arach_FIB, and Pia_FIB1 and 2, which mapped to the regions annotated as LM. In our 19-PCW sample, we observed strong enrichment of Pia_FIB1 and Pia_FIB2 clusters in the LM, with relatively weak predictions for dural layers, which had been removed through dissection. Separation of the dural and leptomeningeal layers is not macroscopically or histologically discernible at earlier stages (6 PCW)^28^, suggesting that these layers probably form within the first trimester between 6 and 9 PCW, consistent with the late differential enrichment of Arach_FIB and Dura_FIB1/2 (Fig. 2c), and their predicted trajectory (Extended data Fig. 4b). These results together bring clarity to the origin, taxonomy and localization of fibroblasts in the human embryonic meninges.

### Cells of the T21 meninges

**Fig. 3:**
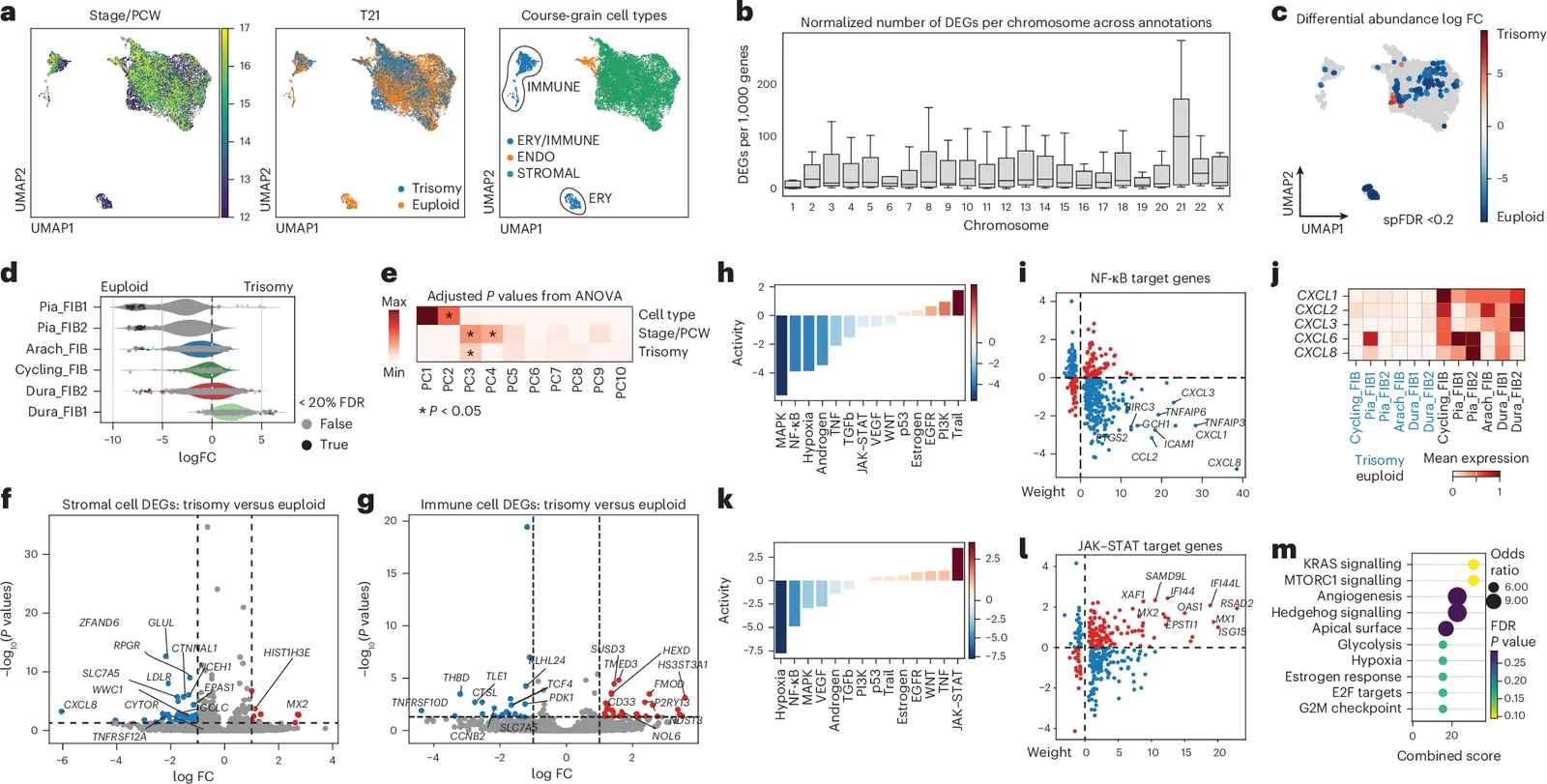
Cells of the T21 meninges. **a**, A UMAP of the T21 samples from 12–17 PCW (12, 15 and 17 PCW, *n* = 3), including age-matched euploid samples (12, 12.5, 14 and 16 PCW, *n* = 4). Left: overlaid metadata of stages, in PCW. Middle: T21 metadata. Right: coarse-grain cell types. **b**, Normalized DEGs per chromosome across cell states. DEGs were divided by 1,000 genes to account for chromosome length. The median is represented by the centre line, box edges show the first and third quartiles and whiskers demonstrate 1.5× the interquartile range. **c**, The log fold change (log FC) shown on a UMAP of neighbourhoods that are differentially enriched between Down syndrome (DS) and non_DS. Neighbourhoods with spatial FDR (spFDR) <0.2 are coloured. **d**, A beeswarm plot of neighbourhoods per stromal cell state. **e**, A heat map of the ANOVA test statistic for each PC of the coarse-grain cell-type pseudobulk samples. **f**,**g**, Volcano plots showing stromal cell (**f**) and immune cell (**g**) DEGs across the two conditions. For DEGs, a two-sided Wald test was performed, and adjusted *P* values are shown. **h**, Pathway analysis for stromal cells. **i**, Target gene correlation with the NF-κB pathway enriched in stromal cells; *x* axis values represent weights, and *y* axis are *t*-values. **j**, Chemokine expression across trisomy and euploid fibroblast cell states. **k**, Pathway analysis for immune cells. **l**, Target gene correlation with the JAK–STAT pathway enriched in immune cells; *x* axis values represent weights, and *y* axis are *t*-values. **m**, Functional enrichment analysis for immune cells.

T21 aneuploidy results in clinical and molecular phenotypes in neurodevelopment and haematopoiesis. Prior single-cell data have illustrated increased haematopoiesis and lymphopoiesis in T21 relative to euploid bone marrow and blood, as well as reduced proliferative capacity in fibroblasts^29–31^. We therefore sought to investigate whether these cellular phenotypes affect the immune and stromal compartments of the developing meninges, which are closely interlinked with in utero neurodevelopment. We first performed age-matched integration of aneuploidy samples (12, 15 and 17 PCW) with euploid samples (12, 12.5, 14 and 16 PCW), and re-embedded the data together to allow direct comparisons (Fig. 3a).

A gene-dosage effect, marked by an increased number of differentially expressed genes (DEGs) attributed to chromosome 21, was observed in the trisomy samples (Fig. 3b). To investigate potential effects of this on cell state abundance, we applied MILO (Methods) to test differences across the euploid and T21 condition. Stromal and erythroid cells were broadly found to be depleted in T21 (Fig. 3c), the latter possibly because most of the droplets were derived from an unsorted 12-PCW sample, whereas the remaining samples all underwent fluorescence-activated cell sorting (Methods) for nuclei enrichment (Fig. 3a). Within stromal cell states, pia fibroblasts, forming the early progenitors of the stromal populations (Extended data Fig. 4), were shown to be most depleted (Fig. 3d), which could suggest that fibroblast development at earlier stages may be more susceptible to T21 effects.

To understand underlying T21 transcriptomic changes governing this, we performed pseudobulking of the compartments and carried out principal component (PC) analysis across metadata (Methods). Cell-type differences and T21 aneuploidy status were both significant (*P* < 0.05) in driving major variance of the data (Fig. 3e). We therefore investigated pseudobulk gene expression differences across T21 and euploid conditions in the stromal and immune cell states (Fig. 3f,g). Numerous genes, including *CXCL8*, shown to mediate fibroblast proliferation in the context of carcinogenesis^32,33^, were downregulated in stromal populations (Fig. 3f). *MX2* was upregulated in T21 stromal cells and has uncertain role in developmental fibroblasts, but has been shown to drive part of the interferon response in T21 (ref. ^34^). Pathway enrichment analysis then showed downregulation of MAPK and NF-κB signalling pathways. Numerous chemokine signalling target genes were correlated with NF-κB downregulation, including *CXCL1*, *CXCL2*, *CXCL3* and *CXCL8*, and were depleted in trisomic versus euploid stromal populations in our data (Fig. 3h–j). Within the setting of inflammatory responses, secretion of these chemokines by fibroblasts promotes neutrophil infiltration^35^. However, specific data and functional implications of this reduced chemokine expression profile in prenatal T21 are lacking. One possibility may be that the reduced chemokine profile serves as a compensatory response to globally increased lymphopoiesis in the T21 state.

Upregulation of JAK–STAT signalling in T21 has been linked to a state of interferon response hyperactivation^34,35^. Presence of the JAK2V617F mutation in blood cancers associated with myeloid proliferation, and the upregulation of JAK–STAT signalling in acute myeloid leukaemia (AML) additionally highlights the gain-of-function consequences of JAK–STAT upregulation on the immune system^36,37^. Therapeutic intervention against AML by inhibition of JAK–STAT signalling has also shown promise clinically^38^. Indeed, individuals with T21 experience an increased risk of AML^39^. In our data, JAK–STAT pathway activity was enriched (Fig. 3k) in the T21 immune compartment, and numerous target genes of interferon signalling (*IFI44*, *MX2*, *OAS*, *ISG15*, *MX1* and *IFI44L)* were associated with the T21 state^40,41^ (Fig. 3l). Functional enrichment using the MsigDB database also highlighted upregulated KRAS signalling, which is known to be constitutively activated in T21-associated leukaemia^42^. We additionally observed MTORC1 signalling enrichment (Fig. 3m). In T21, hyperactivation of MTOR signalling has previously been linked to deficits in MG-mediated processes such as autophagy and mitophagy^43^, although the functional consequences of this remain uncertain.

### Developmental meningeal myeloid niches

**Fig. 4:**
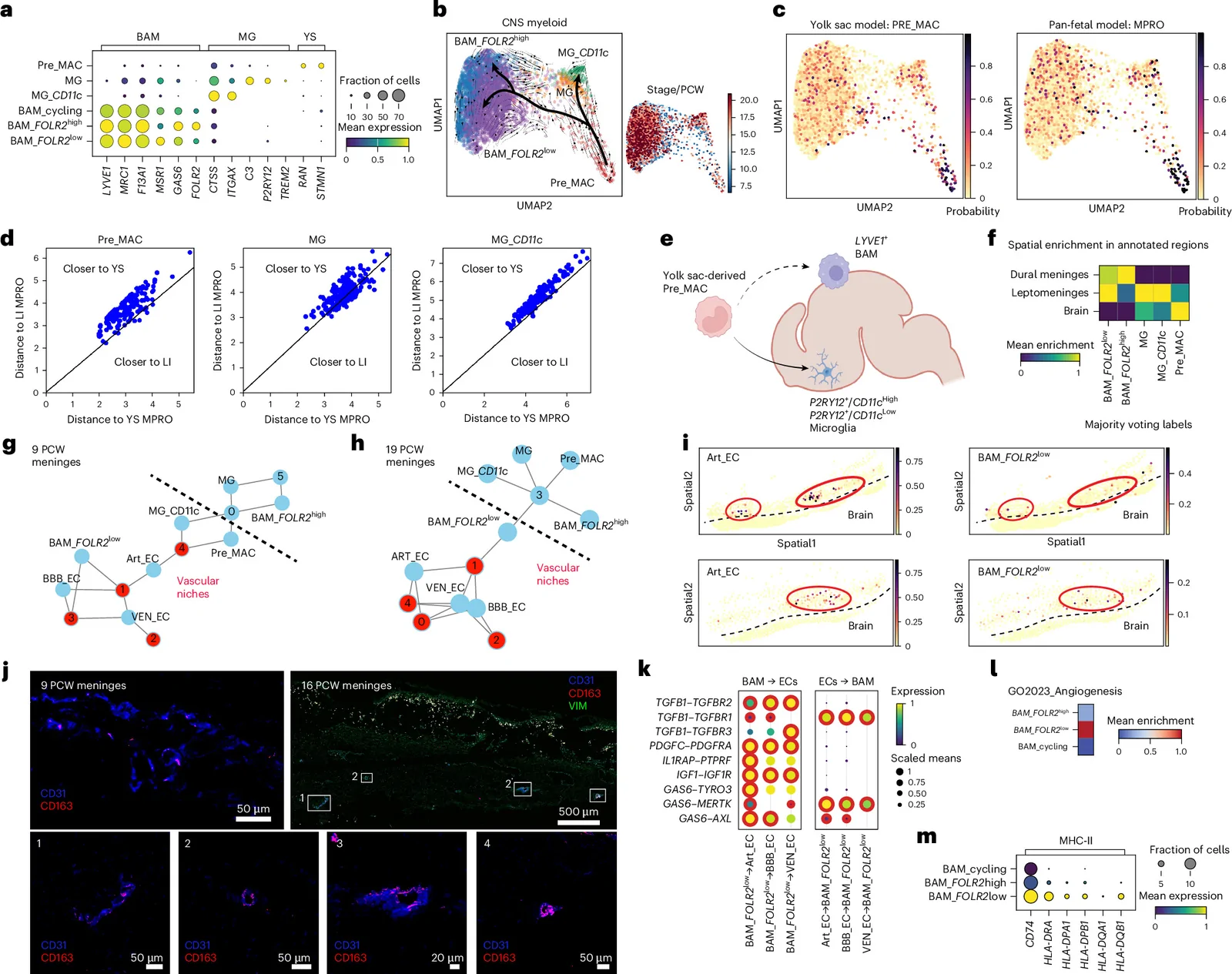
Meningeal myeloid lineage development. **a**, A dot plot of marker genes for myeloid cells from the euploid atlas (*n* = 10). **b**, A UMAP of CNS myeloid cells, with arrows showing pseudotime. Right: sample stage/PCW. **c**, A UMAP of CNS myeloid showing predicted probability of the yolk sac Pre_MAC label from Goh et al. (left) and Suo et al. (right). **d**, A plot of the Euclidean distance of the scVI latent variable between MG_*CD11c*, MG and Pre_MAC scVI to yolk sac (YS) or liver (LI) myeloid progenitors (MPRO) when integrated with myeloid clusters from Suo et al.^10^. **e**, A schematic of YS Pre_MAC ontology in the developing meninges, giving rise to BAM and MG cell states. **f**, A heat map summarizing spatial enrichment of cell states in manually annotated regions of the Xenium data at 9 PCW. **g**,**h**, A plot of membership nodes of spatial niches (numbered) for myeloid and vascular cell states in the first (9 PCW) (**g**) and second trimester (19 PCW) (**h**). The dotted line demarcates niches that include EC states (red), from those that do not, directly. **i**, A spatial plot of label transfer of Art_EC and BAM_*FOLR2*^low^ cell states in the ISS dataset at 9 PCW (*n* = 1). **j**, Protein staining images of PRAMs in the meninges, probably representing BAM_*FOLR2*^low^ (*n* = 2). **k**, CellphoneDB results showing ligand–receptor interaction enrichment per cell-type pair. **l**, Functional enrichment of angiogenesis modules in BAM cell states. **m**, MHC-II expression across BAM populations. Panel **e** created in BioRender; Dufva, O. https://biorender.com/5m83086 (2026).

Clustering of the healthy meningeal myeloid compartment revealed macrophage progenitors (premacrophage (Pre_MAC)), MG clusters and three *LYVE1-*expressing BAMs. The MG clusters expressed *P2RY12* and *TREM2*, both yolk sac markers, suggestive of yolk sac origin^44^. Consistent with this, the Pre_MAC cluster expressed YS macrophage precursor markers *RAN* and *STMN1*, suggesting the possibility that these meningeal-resident YS-derived progenitors may be a source of meningeal myeloid cells. Furthermore, by modelling the pseudotime trajectory, we predict that Pre_MACs may differentiate toward BAMs and MG (Fig. 4a,b).

**Extended Data Fig. 5:**
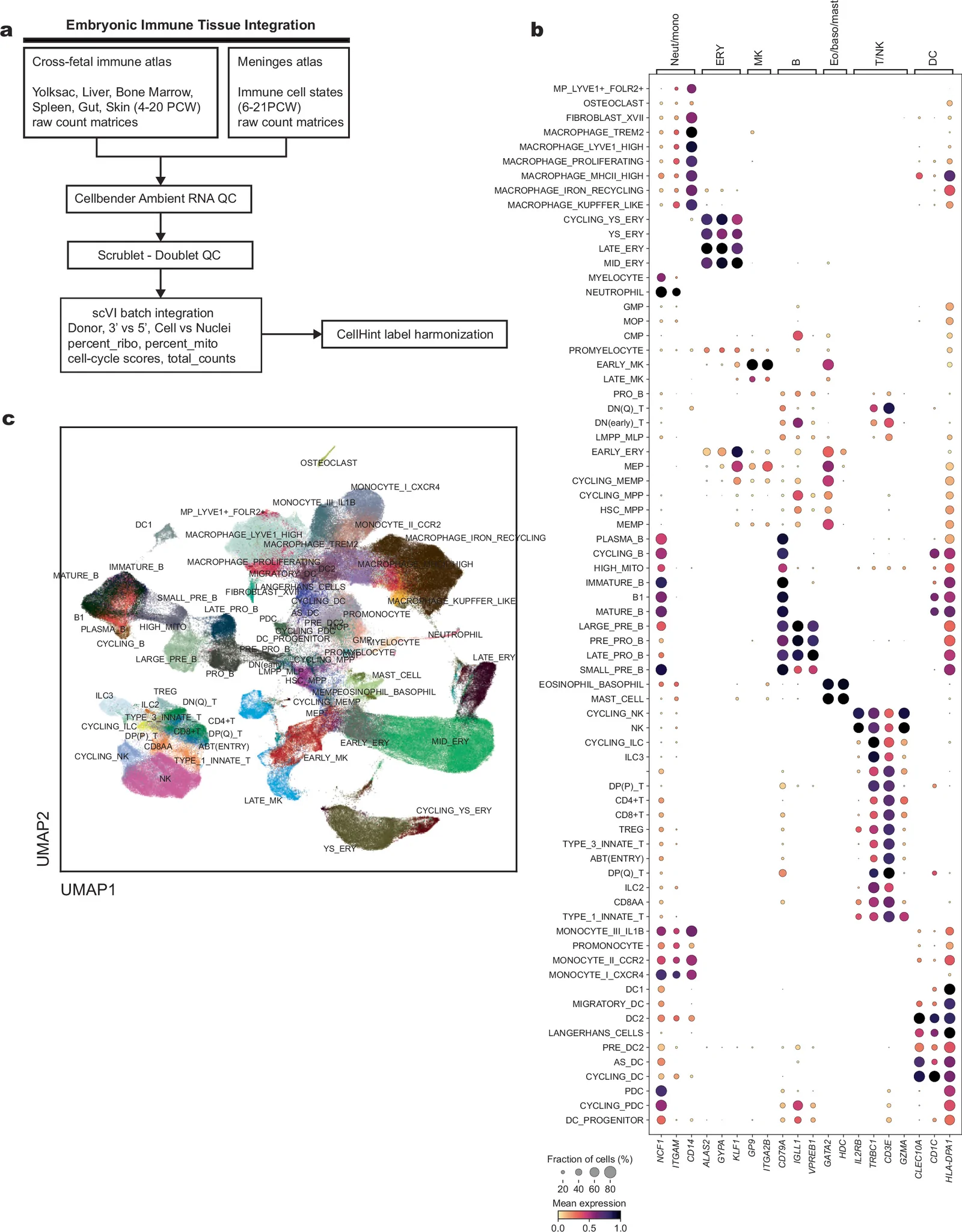
Cross-tissue integration workflow. **a**. Workflow diagram for integration approach, including merging, QC and scVI integration of the meninges atlas (n = 10) with the cross-fetal immune atlas, subsetted for the yolk sac, liver, bone marrow, spleen, gut, skin data. Cellhint was used to harmonize labels. **b**. Dotplot showing marker genes for cell types included in the atlas. **c**. UMAP showing the integrated atlas, with cell state labels.

Pre_MACs also closely resembled yolk sac Pre_MAC and myeloid progenitors transcriptionally (Fig. 4c), gleaned by label transfer from atlases of the yolk sac (Goh et al.), and pan-fetal tissues (Suo et al.)^10,44^. To investigate, we integrated our myeloid compartment with fetal liver and yolk sac data (Extended Data Fig. 5a–c), and calculated comparative Euclidean distance between meningeal myeloid and myeloid progenitors from yolk sac and liver. Consistent with prior reports of MG taxonomy, which focused only on the first trimester, our Pre_MAC and MG clusters were closer and therefore transcriptionally more similar to yolk sac progenitors than liver progenitors (Fig. 4d). These data suggest that yolk sac Pre_MACs may continuously serve as a CNS resident MG/BAM progenitor up to the late second trimester (Fig. 4e).

We next explored spatial niches within which the myeloid cell developed, and found enrichment of BAMs as the major cell types in the manually annotated dural and LM, and MG in the deeper brain parenchyma and LM (Fig. 4f). Leveraging the spatial mapping of cell states, we constructed tissue niche networks with Nichefinder by inferring the distance between cell types and using non-negative matrix factorization (NMF) (Methods) to identify frequently colocalizing populations. Focusing on myeloid lineages and vasculature, we found BAM_*FOLR2*^low^ most connected to vascular cell states, suggesting that they are located within or near vasculature (Fig. 4g). We confirmed this in the in situ sequencing (ISS) spatial dataset (Fig. 4h,i) and protein immunofluorescence staining, which demonstrated PRAMs (CD163) with larger-diameter vasculature (CD31) in the 9 and 16 PCW meninges (Fig. 4j). Single-cell data of the adult brain have highlighted *FOLR2* expression in tissue-resident macrophages; it is therefore likely that the BAM_*FOLR2*^low^ cluster is spatially distinct from BAM_*FOLR*^high^, with the latter associating with the parenchyma of the meninges.

Using cell–cell interaction inference through CellPhoneDB, a number of pro-angiogenic signalling interactions were found to be directed by BAM_*FOLR2*^low^ ligands towards receptors expressed by the EC clusters, including *TGFB1*–*TGFBR1*, *TGFB1*–*TGFBR2*, *TGFB1*–*TGFBR3* and *IGF1*–*IGF1R* signalling pairs^45,46^. ECs were also shown to express *TGFB1* and *GAS6*, the latter of which drives macrophage recruitment^47^ (Fig. 4k). These predicted interactions are therefore suggestive of a functional tissue microenvironment, whereby BAM_*FOLR2*^low^ may be recruited to the perivascular region by ECs, and subsequently promote angiogenesis. We performed gene set enrichment analysis of angiogenesis pathways across BAM populations (Methods) and highlighted enrichment in BAM_*FOLR2*^low^ (Fig. 4l), which also expressed class II MHC strongly (Fig. 4m). Together, these suggest the possible prenatal role of BAM_*FOLR2*^low^ in promoting vasculature formation. It is also tempting to hypothesize, based on MHC expression and their organization in space, that these perivascular BAMs may serve as immune sentinels postnatally to monitor intravascular pathogens.

### Meningeal B cell trafficking

**Fig. 5:**
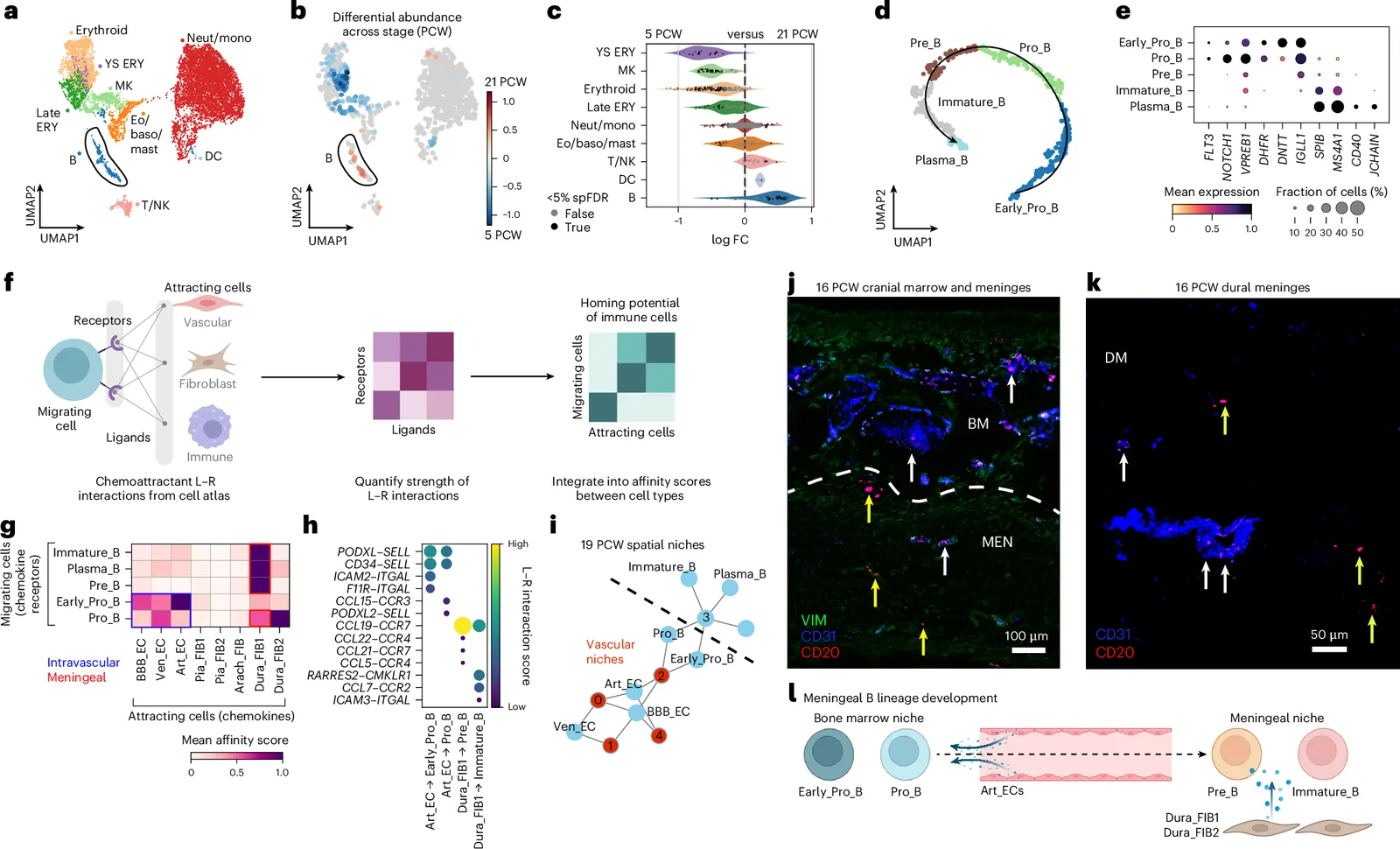
Meningeal B lineage development. **a**, A UMAP of immune cells from the euploid meninges dataset (*n* = 10), with an outline of B lineage cell states. **b**, A UMAP of neighbourhoods from MILO, showing significantly (*P* < 0.05) enriched neighbourhoods (by PCW) overlaid. **c**, A beeswarm plot of MILO results comparing differential neighbourhood PCW enrichment of each cluster. **d**, A UMAP of B lineage populations, with an arrow showing progression from progenitors to mature cell states. **e**, A dot plot of marker genes for B lineage cells. **f**, A schematic of the cell2home computational workflow. **g**, A heat map showing scaled homing affinity scores between migrating B lineage and attracting endothelial and fibroblast cell states. **h**, Cell2home results showing ligand–receptor interaction enrichment per cell-type pair, with the pair with highest contribution to the aggregate homing affinity scores shown. **i**, A plot of membership nodes of spatial niches (numbered) for B lineage and vascular cell states in the 19-PCW spatial data. The dotted line demarcates niches that include EC states (red), from those that do not, directly. **j**,**k**, Protein staining images of B lineage cells (CD20) in the cranial marrow and meninges (**j**) and dural meninges (**k**) at 16 PCW (*n* = 1). **l**, A schematic of B lineage traffic from bone marrow niche to meningeal niche. B, B cells; Baso, basophils; DC, dendritic cells; DM, dura meninges; Eo, eosinophils; ERY, erythrocyte; L–R, ligand–receptor; Mast, mast cells; MK, megakaryocytes; Mono, monocytes; NK, natural killer cells; Neut, neutrophils; spFDR, spatial FDR; T, T cells; YS, yolk sac. Panels **f** and **l** created in BioRender; Dufva, O. https://biorender.com/5m83086 (2026).

Our atlas captured a broad immune repertoire, comprising predominantly myeloid and erythroid cell states, but also included eosinophils, basophils, mast cells, and lymphoid T and B lineages (Fig. 5a). Differential abundance testing across developmental time (21 PCW versus 6 PCW) showed erythroid enrichment in early stages, possibly reflecting the lack of nuclei sorting in the first-trimester samples (Methods), which may have resulted in a greater proportion of red blood cells profiled at the corresponding stages. Notably, B lineages were enriched towards the second trimester (Fig. 5b) significantly, and this was apparent when comparing neighbourhoods directly (Fig. 5c), consistent with previous data of tissue-resident human B lineage development mostly occurring after the first trimester^10^.

B lineage development, including meninges-resident B progenitors, have been observed in adult mouse meninges^13,48^. To resolve fine-grain cell states in our human B lineage population, we subclustered and annotated the droplets by marker genes of B development, showing Early_Pre_B and precursor B cells (Pre_B), marked by *VPREB1*, *NOTCH1* and *FLT3* expression. Pre_B, expressing *IGLL1* and low levels of *DNTT*, were also captured. Immature_B and Mature Plasma_B cells, both expressing *SPIB* and *MS4A1*, which encodes CD20, were abundant towards the latter parts of the B trajectory (Fig. 5d,e).

**Extended Data Fig. 6:**
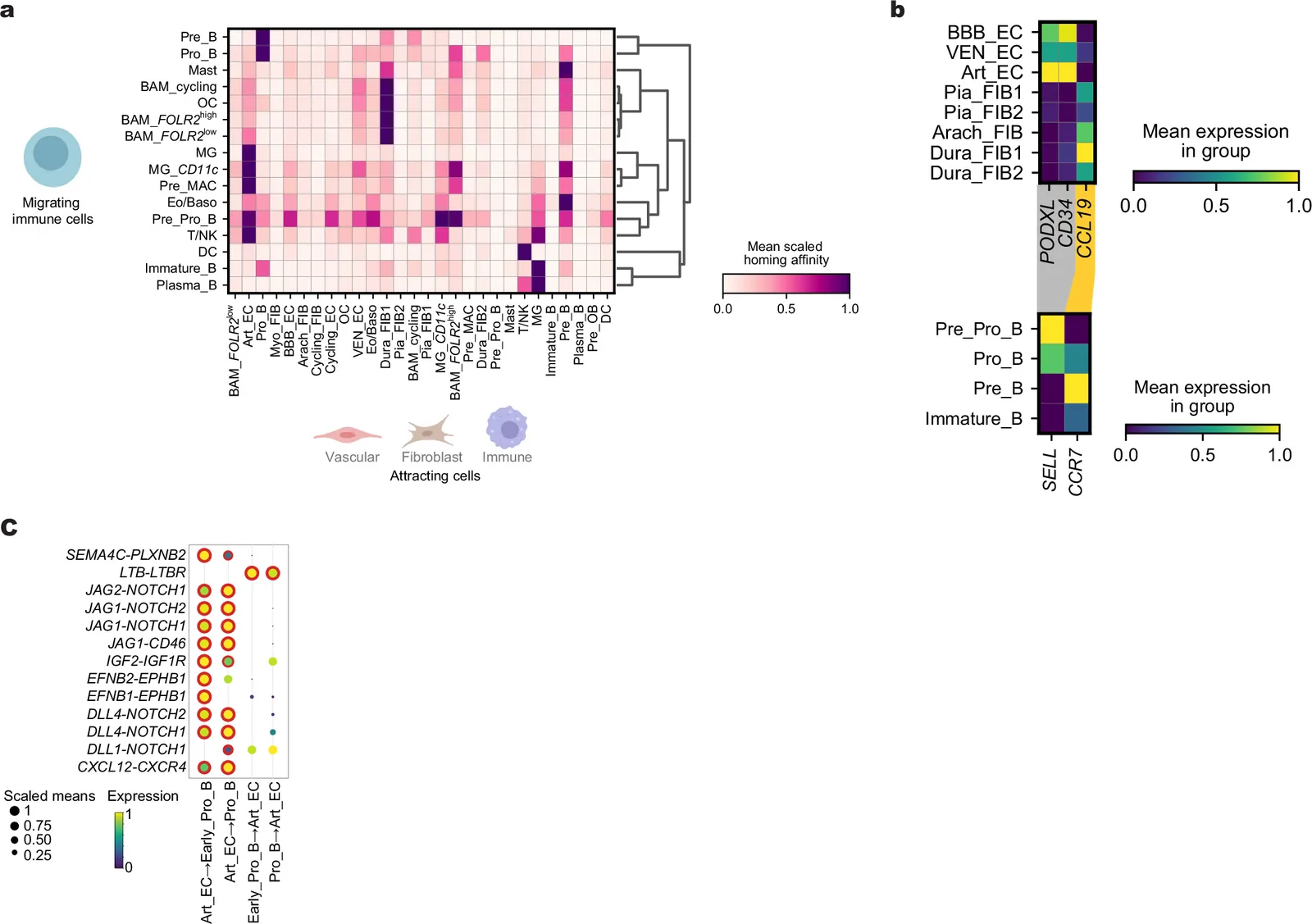
Homing interactions in meningeal immune development using cell2home. **a**. Homing affinity scores of immune cell subtypes towards vascular, stromal and other immune cells in the developing meninges. **b**. Expression of ligands for the top ligand-receptor interactions contributing to B cell homing affinity scores towards endothelial cells and fibroblasts and expression of corresponding receptors in B cell subtypes. **c**. CellphoneDB results showing ligand-receptor interaction enrichment per cell type pair to investigate general cell-cell communication beyond migration signals in the B cell vascular and meningeal niches.

To comprehensively understand the trafficking of immune cells to tissue niches during meningeal development, we developed cell2home, a tool to computationally infer cell–cell homing interactions (Methods). Cell2home predicts which cell types (such as vascular, fibroblast and other immune cells) immune cell subsets are attracted to on the basis of a global chemokine receptor–ligand network. Cell2home first computes receptor–ligand interaction scores by multiplying the expression levels of each chemoattractant receptor with those of their cognate receptors, and then summarizes these homing signals between a migrating immune cell and an attracting cell, repeating this across all migrating and attracting cell types to achieve a global immune cell homing landscape in the developing meninges (Fig. 5f and Extended Data Fig. 6a). We focused on B lineage cells, ECs and fibroblasts to delineate homing signatures driving B cell traffic into the meningeal niche. We found evidence of homing signatures between ECs and Early_Pro_B and progenitor B cells (Pro_B), suggesting that they may be chemoattractant and driving progenitors intravascularly. For the more mature B cell states, including Pre_B and Plasma_B cells, greater chemoattractant signalling from fibroblasts, particularly Dura_FIB1 was detected, suggesting that mature B states are likely to be tissue-resident, partly driven by chemoattractants from meningeal fibroblasts (Fig. 5g). These homing interactions were predicted to be primarily driven by the CD34/PODXL–SELL ligand–receptor pairs between Early_Pro_B/Pro_B and ECs, and CCL19–CCR7 between Pre_B/Immature_B and Dural_FIB1 (Fig. 5h), with a switch from *SELL* to *CCR7* expression upon B cell maturation underlying the change in homing affinity (Extended Data Fig. 6b). Spatial niche analysis showed colocalization of Pro_B with vasculature, providing orthogonal evidence in support of the chemokine predictions (Fig. 5i).

We then studied interactions through which ECs could support the maintenance of B cells after homing to these niches. Multiple signalling pairs were predicted between Art_EC and Pro_B cells, such as JAG–NOTCH interactions, which are involved in vasculature-driven maintenance of haematopoietic cells^49,50^. Similarly, others have proposed that DLL4–NOTCH1 interactions, predicted here, play a key role in vasculature-driven bone marrow haematopoiesis^51^. CD46 is broadly expressed on the cell surface of haematopoietic cells, and the JAG-1 ligand can signal via this to drive an *IFN* response, so it is possible that B lineage cells may be engaged by ECs via this predicted interaction^52^. CXCL12, secreted by bone marrow ECs, have also been shown to specifically drive Pro_B residency in the bone marrow niche, via Pro_B CXCR4, mirroring our results here between Art_EC and Pro_B (Extended Data Fig. 6c). Protein staining for a B lineage marker (CD20) in our meninges sections revealed B cells in 16-PCW cranial marrow, colocating with vasculature within the marrow and meninges. Dural meningeal-resident and extravascular B cells were also noted (Fig. 5j,k). These data demonstrate a close physical association between developmental B cells with vasculature, and hint at the possibility that they may travel into the meningeal niches through vasculature from the bone marrow, and mature B cells, may, through chemoattraction mediated by fibroblasts, remain resident in the meningeal parenchyma (Fig. 5l).

## Discussion

A broad plethora of cell types were captured in this multiomic atlas of all developing layers of the meninges across the first and second trimester. We revealed the scope of major immune cell types, including B and T cells, enriching particularly in the second trimester, consistent with observations in other fetal tissues^10^. Others have atlased human leptomeningeal development, and correlated arachnoid and pia cell types to those described in single-cell mouse atlases. Here, we further this by capturing fibroblasts across the dural layer through developmental time. Through single-cell resolution spatial mapping in our ISS data, we were able to obtain direct confirmation that the pia, arachnoid and dura fibroblast clusters corresponded to spatially distinct layers. However, the earlier neural crest progenitors of the anterior cranial meninges were not captured, and future studies that profile human developmental stages before 5 PCW will facilitate their characterization. Our droplet data of these spatially resolved cell states allowed their ontogeny to be clarified, showing that early *HIC1*-enriched pia fibroblast cell states precede *PI16*-expressing arachnoid and dura fibroblasts, mirroring fibroblast development in the joints. This cross-tissue homology warrants exploration, and is achievable by large-scale integration of fetal atlas data, parallel to what has been achieved in cataloguing cross-tissue adult fibroblast lineages^53^. Such an analysis may offer insights into the role of fibroblast subsets in adult inflammatory disease.

Within the myeloid lineage, we observed shared origins between the MG and BAM of the meninges. We defined a Pre_MAC progenitor expressing *STMN2* and *P2RY12*, both markers of yolk sac progenitors. Trajectory analyses suggested that this gave rise to BAMs and MG in our data. Integration of multiple published single-cell atlases supported our hypothesis that Pre_MACs are probably yolk-sac-derived progenitors. Indeed, lineage tracing in human data, if possible in some way, would provide new insights into these possible lineage relations. However, current studies in human embryos are limited by logistical challenges of studying tissue samples from very early development, in the case of the yolk sac, for example, sampling is required at 4 PCW. Limitations related to the error rates of sequencing technologies also do not allow us to detect mutations present only in a small number of cells, as these become overwhelmed by noise. Our analyses of BAMs allowed us to map these in space, and identify a subset of BAMs (BAM_*FOLR2*^low^) within perivascular niches. We hypothesize that these coordinate meningeal vascularization during fetal development, and postulate as to their possible role in postnatal immunity. Across human fetal development, a comparable cross-tissue PRAM, also marked by expression of *VEGFA*, has been characterized^54^. However, in that study, only meningeal tissues at 6 PCW were profiled. Regardless, PRAMs were observed to localize to leptomeningeal vessels at ~6 PCW. Our work has helped develop further insights into this population by demonstrating their cross-time developmental trajectory and shared origins with MG.

Nearly all parts of the B lineage, stemming from progenitor B populations, were identified in our droplet data, consolidating the meninges as a key tissue for embryonic and fetal B lineage development. Using protein staining, we confirmed their abundant presence in both the cranial marrow and underlying dural meninges during human development. This, to our knowledge, has not been described previously in humans, and warrants a substantial amount of future exploration. For example, there are major outstanding questions relating to the functional implications of this developmental niche, the consequences of perturbation and the postnatal destiny and immune role of meningeal-resident B lineage cells. We were able to use the gene expression droplet dataset here to glean insights into the chemotaxis signatures of each B cell–EC and B cell–fibroblast pair, and show that B progenitors, possibly from the bone marrow, are attracted into the blood vessels, whereas more developed cell states are attracted by the meningeal fibroblasts, possibly hinting at a mechanism for traffic out of the bone marrow and subsequent meningeal tissue residency. Confirmation of this may request the study of mouse meningeal development, and use of perturbation or knockout models to interrogate such a proposed mechanism. Lineage tracing in mice might represent one exciting avenue to probe the destinations of the B progenitors within the developing mouse cranial bone marrow. This, however, may not necessarily be homologous to human development. In human tissues, use of B cell receptor sequencing of cell-sorted B lineage droplets will allow correlation of clones in the bone marrow and meninges, shedding light on the possible sharing of developmental progenitors between the two niches.

We developed cell2home to provide insights into homing interactions establishing B cell localization in vascular and fibroblast niches in the developing meninges. This computational approach is based on the key biologically grounded insight that the functional output of chemoattractant receptor signalling in response to ligand engagement, across all of the different receptors, is to increase migration of the receptor-expressing cell towards increasing density of the ligand. Therefore these ligand–receptor interactions with identical outputs can be aggregated together into a single homing affinity score between two cell types. The homing affinity score can then be used to identify which cell type a given cell is predicted to home towards. While tools such as CellPhoneDB and CellChat answer the question of which ligand–receptor interactions are predicted to occur between two cell types, cell2home answers the question of which cell types are predicted to attract a given cell to home towards them. We envision cell2home to have utility in deciphering how immune cells are recruited to tissue niches during development across organs as well as in inflammation and cancer.

Our mapping of T21 developmental meninges revealed increased signalling in JAK–STAT pathways, and upregulation in interferon signalling genes, that may explain the myeloproliferative phenotype observed in T21. Our finding of upregulated MTOR signalling is less clear. Increased MTOR signalling has been shown to be associated with insulin resistance in T21, possibly explaining the metabolic phenotype^55,56^. However, robust functional experiments are needed to investigate. Confounders to these possible T21 effects include maternal factors, genetic background, gestational stage and sex, and therefore, future work targeting larger-scale profiling of stage- and sex-matched aneuploidy states, as well as normal meningeal development, will accelerate research into this important area of human developmental biology.

Together, our data define the developing human meninges as a niche characterized by stromal and immune diversity, providing a reference for future studies of human development and beyond.

## Methods

### Sampling and ethical approvals

First-trimester (<12 PCW) meninges samples were obtained from elective terminations under REC 96/085 with written and informed consent obtained from all sample donors (East of England, with full approval from the Cambridge Central Research Ethics Committee). Second-trimester tissues (>12 PCW) were obtained from the joint MRC/Wellcome Trust Human Developmental Biology Resource (HDBR) (MR/R006237/1, REC:18/LO/0822 and 2008/05/HDBR, IRAS:326492; http://hdbr.org) at the Institute of Child Health, London (registration number 200683) under full ethical approval from the London-Fulham Research Ethics Committee. Karyotype information was provided by the HDBR for all second-trimester samples, allowing assignment of ploidy and sex status. For assignment of samples in the first trimester, not obtained from the HDBR, gene expression of chromosome Y genes was used as a surrogate (Supplementary Table 2). All meningeal samples were dissected under microscope vision, and meningeal layers were lifted using microforceps, off brain tissue, and separated from cranial bone. Samples were manually dissected into anterior and posterior portions. Samples were then suspended in optimal cutting temperature embedding medium and frozen in a mixture of isopentane and dry ice.

### Single-nucleus isolation and library preparation

Single nuclei were isolated from fresh-frozen samples through cryosectioning followed by mechanical dissociation as described in previous work^57^. In brief, 10-μm sections were homogenized in homogenization buffer (250 mM sucrose, 25 mM KCl, 5 mM MgCl_2_, 10 mM Tris–HCl, 1 mM dithiothreitol, 1× protease inhibitor, 0.4 U μl^−1^ RNaseIn, 0.2 U μl^−1^ SUPERaseIn and 0.1% Triton X-100 in nuclease-free water) using a glass Dounce tissue grinder (Merck). The resulting suspension was passed through a 50-μm cell strainer, followed by centrifugation (500*g* for 5 min). The pellet was then resuspended in 300 μl of storage buffer (1× PBS, 4% BSA and 0.2 U μl^−1^ Protector RNaseIn) and passed through a 20-μm cell strainer. For samples within the first trimester of gestation (<12 PCW), the resulting single-cell suspension contained minimal debris upon visual inspection under the microscope. However, for second-trimester samples (>12 PCW), substantial amounts of debris were present within the single-cell suspension, and nuclei enrichment was performed, using a Thermo Fisher Bigfoot Spectral Sorter following 7-AAD staining (BioLegend 420403). Nuclei were then processed for 10x Genomics Chromium Single Cell Multiome ATAC + Gene Expression assay according to the manufacturer’s protocol. A subset of samples were processed as mixtures of droplets from multiple separate donors within one reaction and subsequently demultiplexed by genotype. Quality control of cDNA and final libraries was carried out using Bioanalyzer High Sensitivity DNA Analysis (Agilent). Libraries were sequenced using a NovaSeq 6000 (Illumina) with a minimum sequencing depth of 20,000 read pairs per droplet.

### Multiplex immunofluorescence staining

Fresh-frozen tissue blocks were utilized for multiplex immunofluorescence analysis using the RareCyte 16-plex platform. Cryosections of 10 µm thickness were mounted onto Superfrost slides, air-dried for 10 min and fixed in PFA fix buffer to promote tissue adherence. Autofluorescence was reduced by bleaching slides in AF Quench Buffer (4.5% H_2_O_2_ and 24 mM NaOH in PBS) under high-intensity white light for 60 min, followed by UV illumination at 365 nm for 30 min. The sections were subsequently stained with a 16-plex panel of custom-formulated ArgoFluor-conjugated antibodies (RareCyte) following the manufacturer’s protocol. The following antibodies were utilized: CD31 (2.0 μg ml^−1^; EPR5251, Abcam, lot: 210910), CD20 (1.0 μg ml^−1^; L26, eBioscience, lot: 210429), CD163 (2.0 μg ml^−1^; EPR14647, Abcam, lot: 210910), Vimentin (2.0 μg ml^−1^; 091d3, Biolegend, lot: 3) and ACTA2 (1A4, RareCyte in-house). Nuclei were counterstained with Hoechst dye. Stained slides were imaged using the RareCyte Orion system equipped with seven excitation lasers. The slides were imaged the following day using a RareCyte Orion microscope equipped with a 20× objective. Image acquisition was performed using Imager software and data preprocessed with Artemis 4.0 software (RareCyte) to correct for autofluorescence and spectral crosstalk among fluorescence channels.

### Xenium spatial transcriptomics

Formalin-fixed, paraffin-embedded tissue samples (Supplementary Table 4) were sectioned onto Xenium slides (10x Genomics, 3000941) at a thickness of 5 µm and stored at room temperature in desiccant for up to 4 weeks. These were then processed using the Xenium Sample Preparation Kit (10x Genomics, 1000460) as per the manufacturer’s protocols (CG000580 for pretreatment, including deparaffinization and decrosslinking, followed by CG000582 for probe hybridization and rolling circle product generation). Following autofluorescence quenching and nuclei counterstaining as per the manufacturer’s instructions (CG000582), slides were transferred onto a Xenium Analyzer instrument alongside decoding reagents (10x Genomics, 1000461) and decoding consumables (1000487) all prepared according to the manufacturer’s protocol (CG000584). Following the automated assay, in which cycles of hybridization and imaging are performed to assign transcript identities to single RNA spots using a fluorescent barcode readout, data were exported from the instrument in the vendor format of ome.tif images (DAPI-stained nuclei), .h5-format cell–gene count matrices and .csv-format cell and transcript data.

### ISS imaging decoding

We used the ISS decoding pipeline outlined in Li et al.^58^. This pipeline consists of five distinct steps. First, we performed image stitching using Acapella scripts provided by PerkinElmer, which generated two-dimensional maximum-intensity projections of all channels for each cycle. Next, we used Microaligner (https://github.com/VasylVaskivskyi/microaligner) (v.1.0.0) to register all cycles on the basis of DAPI signals using the default parameters. For cell segmentation, we utilized a scalable algorithm that leverages CellPose (v.3.0)^59^ as the segmentation method. The expected cell size is set to 70 pixels in diameter and further expanded 10 pixels to mimic the cytoplasm. To decode the RNA molecules, we used the PoSTcode algorithm^60^ (v.1.0) with the following parameters: rna_spot_size = 5, prob_threshold = 0.6, trackpy_percentile = 90 and trackpy_separation = 2. Furthermore, we assigned the decoded RNA molecules to segmented cells using STRtree (v.2.0.6) and subsequently generated AnnData objects following the approach described by Virshup et al.^61^. Finally, only cells with more than four RNA molecules were kept for downstream analysis.

### Droplet data preprocessing

Sequencing data were aligned to the human reference genome (GRCh38-2020-A-2.0.0) using CellRanger ARC software (v.2.0.0). The called barcodes from 10x Multiome lanes with pooled genotypes from multiple sample donors were demultiplexed per genotype using BAM outputs through Souporcell (v.2.0)^62^. Subsequently, the Souporcell outputs were clustered by genotype for metadata assignment to each barcode. For gene expression data, cellbender (v.0.2.0)^63^ was applied to remove background ambient RNA. Droplets were filtered for more than 200 genes and less than 5% mitochondrial and ribosomal reads. Doublet removal is described below.

### Doublet detection

All potential doublets detected in both RNA and ATAC modalities were removed from our data. For RNA data, Scrublet (v.0.2.3)^62,64^ was applied to estimate doublet probability, and a score of more than 0.3 was used as a cutoff value. To apply a stringent doublet threshold, we conducted an adapted Scrublet workflow as previously described^65^. In brief, per-droplet Scrublet scores were first determined for CellRanger ARC count matrices from each 10x Multiome (gene expression) lane independently. The droplets were then overclustered through the standard scanpy^66^ workflow using default parameters up to Leiden clustering. Each individual cluster was further clustered. A per-cluster median of Scrublet scores was computed. A normal distribution of doublet score, centred at the score median with a standard deviation estimated from the median absolute deviation, was used to compute *P* values for each of the clusters. After false discovery rate (FDR) adjustment using Benjamini–Hochberg correction, a *P* > 0.65 was deemed as a cutoff value of good-quality cells, as doublets were significant outliers.

### Droplet cluster annotation

We adopted a hierarchical clustering approach by first conducting Leiden clustering on the global integrated scVI RNA embedding to obtain cell-type-level clusters. We utilized canonical marker genes to annotate these large clusters and subset for cell types. For each cell-type cluster, for example, fibroblasts, we further embedded each subset using scVI and conducted Leiden clustering (resolution = 0.5 to 1.0), followed by DEG analyses (method = ‘wilcoxon’) to obtain cluster markers. We also utilized the inferred spatial location of cell states (described below) to inform annotations.

### Differential abundance testing

We applied the Python implementation of the MILO package (v.0.1.1) for differential abundance testing (http://github.com/emdann/milopy). We used the scVI latent representation to create a *k*-nearest neighbour graph of droplets in the relevant compartment and subsequently applied milopy to allocate droplets to overlapping neighbourhoods, with these droplets originating from multiple donor samples. Each neighbourhood was then annotated as a cluster on the basis of majority voting. We binarized values for comparator covariates (for example, anterior versus posterior) to allow testing across these variables. We then determined log fold-change values for differential abundance and FDR values on the basis of the Benjamini–Hochberg correction.

### Pseudobulk enrichment analysis for trisomy samples

For the age-matched trisomy-euploid atlas, containing 12–17-PCW samples, the Python implementation of decoupler was used to perform pseudobulking with default parameters^67^. In brief, pseudobulk profiles were generated for each cell type and donor sample. PCs were then computed, and each inferred PC was tested against covariates individually, including PCW stage, trisomy and cell type, which captured a significant degree of variance (PC1, PC2 and PC3). Default parameters were utilized to filter for genes by min_total_counts = 15. Differential expression analysis was conducted using the Python implementation of DESeq2^67,68^. For pathway enrichment analysis, we utilized the PROGENy pathway database, and calculated pathway scores using the univariate linear model (ulm) function within the decoupler package^69^.

### GRN analysis

The SCENIC+^70^ pipeline was used to predict transcription factors and target genes. The fragment matrix of peaks was called using MACS2 and then processed within ArchR^71^ together with the matched RNA count matrix as inputs. Metacells were created by clustering droplets into groups of around 10–15 droplets on the basis of their RNA profiles and were then aggregated for both counts and fragments. The pipeline was applied to subsets of the dataset corresponding to each cell type. pyCisTopic was used to identify region topics and differentially accessible regions from the fragment counts as putative regions for transcription factor binding. CisTarget was used to scan the regions for transcription factor-binding sites, and GRNBoost2^72^ was used to link transcription factors and regions to target genes on the basis of co-expression or accessibility. Enriched transcription factor motifs in the regions linked to target genes were used to construct transcription factor–region and transcription factor–gene regulons. Finally, regulon activity scores were computed with AUCell on the basis of target gene expression and target region accessibility, and regulon specificity scores were derived from them. Networks of transcription factors, regions and target genes (enhancer-driven GRNs (eGRNs)) were constructed by linking individual regulons.

### Label transfer using CellTypist

For characterization of meningeal EC states, we performed automated annotation using a CellTypist model obtained from authors of an integrated atlas of human vascular cell types^16,17^. Default parameters of the celltypist.annotate() function within CellTypist 1.7.1 was used. Marker gene expression was then used to evaluate transferred labels.

### Trajectory analysis

For pseudotime trajectory construction in the myeloid and fibroblast compartments, non-cycling droplets were removed, and X_scVI was used as a projection for Palantir to obtain diffusion space. A neighbourhood graph was generated on the diffusion space using sc.pp.neighbors, and the first two PCs were used as initial positions to create ForceAtlas2 embeddings using sc.tl.draw_graph. For diffusion pseudotime, scFates^73^ (v.1.0.3) was used to predict a principle graph that captures the differentiation path. Within the myeloid lineage, Cellrank, using the CytoTRACE kernel, was utilized to predict differentiation potential and pseudotime^74^.

### Cell homing analysis using cell2home

To predict homing of migrating immune cells towards attracting cells, we developed an analysis toolkit, cell2home (implemented as a Python package: https://github.com/Teichlab/cell2home). As a biological function-driven basis for the computational framework, we curated chemoattractant receptors from the literature into a homing interaction dictionary. We included only chemoattractant receptors that have been demonstrated to regulate migration of affinity towards cells expressing their cognate ligands to be able to summarize these signalling inputs from a variety of receptors into a single functional output: cell migration. These receptors include the CC, CXC, CX3C and XC chemokine receptor families as well as adhesion-mediating integrin receptors. Each ligand was linked to its cognate receptors using CellPhoneDB. This homing interaction dictionary is provided as part of the cell2home package.

First, a reference of chemoattractant ligand expression levels across attracting cell types is generated by pseudobulking the single-cell transcriptomes per cell type (and optionally additional cell metadata variables such as donor groups) through summing raw counts (with an option to pseudobulk using mean log-normalized counts for applicability across dataset types). The package comes with a streamlined, matrix operation-based function for category-level pseudobulking capable of quick, memory-efficient processing of larger datasets. This is followed by normalization, log1p transformation, scaling and extracting the resulting expression levels of the chemoattractant ligands for each pseudobulk profile. Ligand–receptor interaction scores are then computed by multiplying the expression of each receptor in the single cells with the cognate ligand expression in the reference cell types. The resulting interaction scores are averaged for each receptor across all ligands of the receptor in a given reference cell type to summarize all incoming migration cues received by one receptor from an attracting cell (receptor homing affinity). Subsequently, the receptor level scores are aggregated, taking the mean by attracting cell type to obtain scores measuring predicted affinity of the single cell to all attracting cell types. The resulting scores, including (1) ligand–receptor interaction scores, (2) receptor homing affinity scores and (3) final cell-level homing affinity scores are stored in a MuData object to facilitate downstream analyses and visualization. Using the MuData object, cell2home allows extraction of the ligand–receptor pairs with the strongest predicted contribution to a homing interaction between two cell types and ranking of these interactions on the basis of the ligand–receptor interaction scores.

### Nichefinder

**Extended Data Fig. 7:**
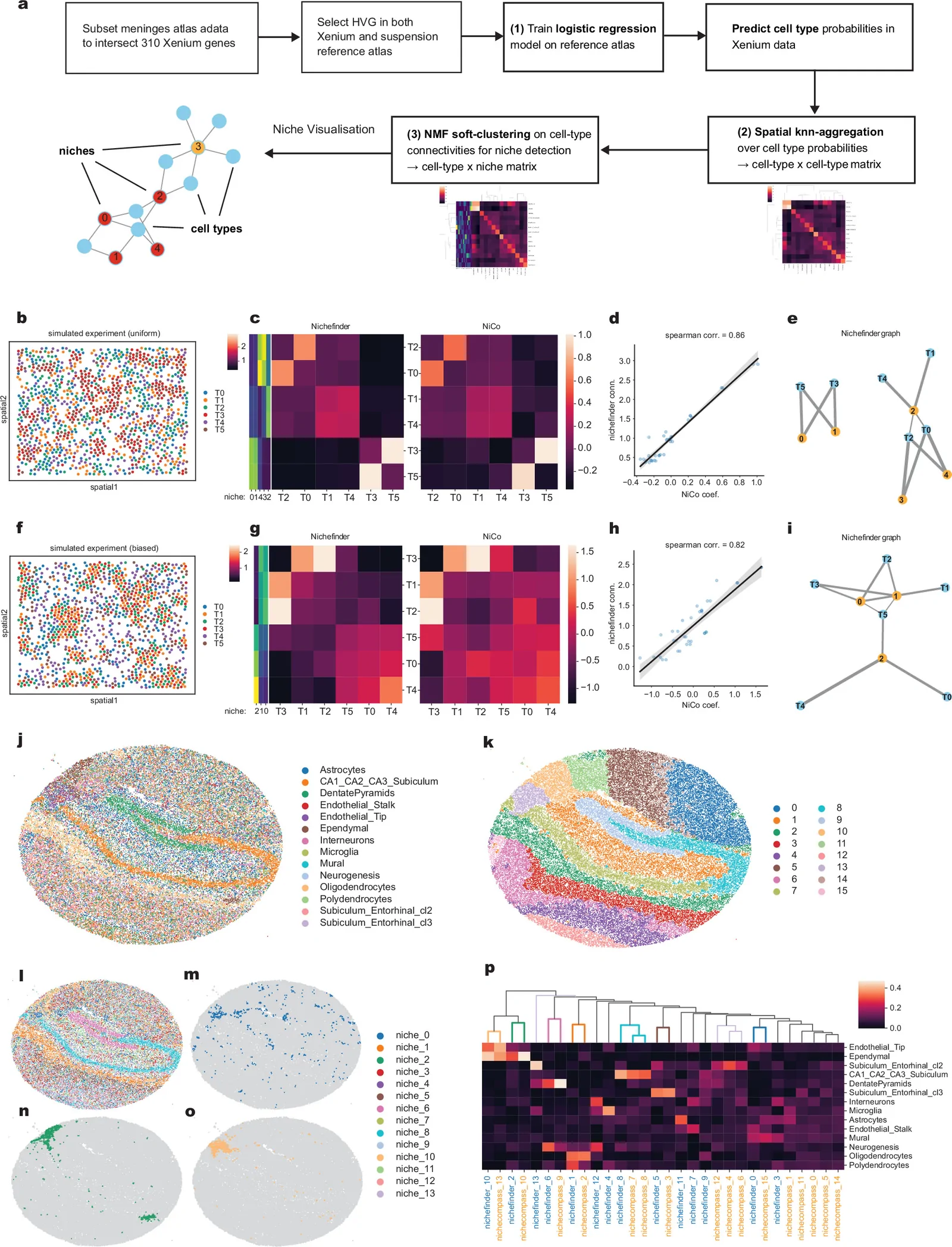
Nichefinder workflow. **a**. Nichefinder workflow showing highly variable gene (HVG) selection, followed by label predictions and negative matrix factorization (NMF) to identify spatial niches. **b**. Simulated spatial transcriptomics data (uniform) from^84^. **c**. Cell type interaction matrices predicted by nichefinder and NiCO for b. **d**. Scatter plot and correlation between nichefinder and NiCO cell type interaction matrices for c. Bands around the regression line show the 95% confidence interval. **e**. Bipartite graph representation of d. **f**. Simulated spatial transcriptomics data (biased) from^84^. **g**. Cell type interaction matrices predicted by nichefinder and NiCO for f. **h**. Scatter plot and correlation between nichefinder and NiCO cell type interaction matrices for g. Bands around the regression line show the 95% confidence interval. **i**. Bipartite graph representation of i. **j**. Cell type labels on mouse hippocampus data^88^. **k**. NicheCompass niche predictions for j. **l**. Nichefinder niches mapped back to individual cells using the cell type probability matrix. **m-o**. Nichefinder niches 0, 2 and 10 mapped to individual neighborhoods (each cell and its k = 5 nearest neighbors) using a geometric mean weighted by niche-membership weights. **p**. Clustering of nichfinder and NicheCompass niches based on niche membership distributions, showing matches as well as distinct branches.

To quantify the interactions between cell types in our atlas on each spatial slide, we sought a direct and simple method. Most existing tools frame niche detection as a problem of labelling each cell with a categorical niche label. However, as our main goal was to contextualize the cell states annotated within the atlas using spatial information, we defined niche detection in a more abstract way, as identifying motifs of colocalizing cell types that re-occur across a slide. To this end, we set up a modular analysis pipeline of independent steps (Extended Data Fig. 7a–p), combining (1) cell-type label transfer, (2) spatial *k*-nearest neighbours (*k*-NN) aggregation and (3) soft-clustering of cell-type interactions (implemented as a Python package for reusability: https://github.com/Teichlab/nichefinder).

### Label transfer

First, for label transfer (1), we train a logistic regression classifier on a reference dataset to predict cell types for each cell in the ISS dataset, an established strategy for automatic cell-type annotation on single-cell data^17^. To make best use of the returned information, we base subsequent steps on the full probability distribution of cell-type assignments rather than using only the cell type with highest probability. Given the limited plexity of the ISS data, we subset genes for logistic regression training to highly variable genes (HVGs) shared between the two datasets. Using scanpy ‘sc.pp.highly_variable_genes’ with ‘min_disp=0.1’ for HVG detection on both datasets, this yields around 30–100 overlapping HVGs, depending on gene expression complexity of the analysed section.

### *k*-NN aggregation

Next, for spatial contextualization (2), we apply spatial *k*-NN aggregation in an approach similar to PAGA^21^, which is commonly used to analyse cell-type relationships in suspension data. When constructing the cell type-by-cell type connectivity matrix, we make use of the full probability matrix of cell-type assignments from (1) and further normalize by the expected number of connectivities to account for imbalances in cell-type label abundance. For spatial *k*-NN aggregation, nichefinder also requires a precomputed spatial *k*-NN graph in the AnnData.obsp[‘connectivities’] slot, which can be easily achieved using scanpy (sc.pp.neighbors) or squidpy (sq.gr.spatial_neighbors). Here, we use default scanpy settings on two-dimensional coordinates with *k* = 5 to capture proximal interactions. While this uses a uniform manifold approximation and projection (UMAP)-style connectivity kernel^10^, producing a sparse weighted connectivity matrix C, nichefinder is agnostic to the weighting scheme as long as C≥0. The cell-type interaction network is then obtained by a single bilinear projection:

$$A={P}^{{\rm{T}}}{CP},$$

where $A\in {{\mathbb{R}}}^{t\times t},\,A\ge 0$ is the resulting cell-type interaction matrix, $C\in {{{{\mathbb{R}}}}}^{n\times n},\,C\ge 0$ the spatial connectivity matrix and $P\in {{{{\mathbb{R}}}}}^{n\times t},\,P\in \left[\mathrm{0,1}\right]$ the cell-type probability matrix for n cells, t cell types and k niches. When using probabilistic cell-type assignments in P, cells with fractional type membership contribute proportionally to every pairwise interaction term. Note that fixed cell-type assignments can be easily used, too, by setting the probability of the respective assignments to 1.

To remove trivial enrichment arising from cell-type abundance differences, nichefinder normalizes A by a randomized background model ${A}^{\mathrm{rand}}$. In addition, we introduce a parameter $\delta \in [\mathrm{0,1}]$ to reduce the weight of self-interactions, which may dominate in slides with spatially segregated cell types, and focus on more informative cross-cell-type interactions. The default of δ=0 corresponds to no reduction of same-cell-type interactions, 1 to complete removal and values in between to partial reduction. With this, the random background normalization is calculated as follows:

Step 1, estimate marginal frequencies ${\hat{p}}_{b}\in {{{{\mathbb{R}}}}}^{t}$ from a diagonal-deflated copy ${\tilde{\rm{A}}}\in {{{{\mathbb{R}}}}}^{t\times t}$:

$${\tilde{A}}=A-\delta {\mathrm{diag}} (A),{\hat{p}}_{b}=\frac{{\sum }_{a}{\tilde{\mathrm{A}}}_{{ab}}}{{\sum }_{a,b}{\tilde{\mathrm{A}}}_{{ab}}}.$$

Step 2, form the random background matrix ${A}^{\mathrm{rand}}\in {{{{\mathbb{R}}}}}^{t\times t}$, calculating non-diagonal and diagonal elements as:

${A}_{{ab}}^{\mathrm{rand}}={\hat{p}}_{a}\mathop{\sum }\limits_{c}{{A}_{ca}}$ for a≠b and otherwise

$${A}_{{aa}}^{\mathrm{rand}}=\delta{A}_{{aa}}+(1-\delta ){\hat{p}}_{a}\mathop{\sum }\limits_{c}{{A}_{ca}}.$$

Step 3, element-wise normalization into a matrix ${\hat{A}}\in {{{{\mathbb{R}}}}}^{t\times t}$:

$${\hat{\mathrm{A}}}_{{ab}}=\frac{{A}_{{ab}}}{{A}_{{ab}}^{\mathrm{rand}}}.$$

After that, values ${\hat{\mathrm{A}}}_{{ab}} > 1$ indicate colocalization enrichment and values <1 indicate depletion relative to the spatial random baseline. Thus, ${\hat{A}}$ constitutes a cell type-to-cell type adjacency matrix that quantifies the spatial association strength between pairs of cell types.

### Niche clustering

Finally, for niche clustering (3), the normalized cell type-by-cell type matrix ${\hat{\mathrm{A}}}\ge 0$ is factorized using NMF as:

$$\hat{\mathrm{A}}\approx WH,W\in {{{{\mathbb{R}}}}}^{t\times k}\ge 0,H\in {{{{\mathbb{R}}}}}^{k\times t}\ge 0,$$

using the NMF implementation in sklearn^75^, which minimizes the Frobenius reconstruction error ${\min }_{W,H\ge 0}{\parallel \hat{\mathrm{A}}-WH\parallel }_{\mathrm{F}}^{2}$. If desired, the optimal rank k can be selected as the elbow of the reconstruction-error curve ${\left\{{{||\hat{\mathrm{A}}}-{W}_{k}{H}_{k}{||}}_{{\rm{F}}}\right\}}_{k=1}^{{k}_{\max }}$ via the second discrete-derivative method. In the case of a directed, asymmetric cell-type adjacency matrix $\hat{\mathrm{A}}$, the resulting matrices W and H could be interpreted as sender and receiver profiles of niches. However, for the standard *k*-NN-based colocalization analysis performed here,

W and H are blended into a single niche-membership matrix $S\in {{{{\mathbb{R}}}}}^{t\times k}$:

$$S=\alpha {{\mathrm{col}}\_{\mathrm{norm}}}(W)+(1-\alpha ){{\mathrm{col}}\_{\mathrm{norm}}}({{H}^{\rm{T}}}),$$

where α=0.5. This is justified if $\mathrm{col\_norm}(W)\approx \mathrm{col\_norm}({H}^{{\rm{T}}})$, which is usually the case for symmetric $\hat{\mathrm{A}}$, and can be assessed via the mean column-wise cosine similarity between col-normalized W and ${H}^{{\rm{T}}}$; values close to 1 (>0.95) indicate that the two factors encode equivalent niche profiles. As an alternative, we implemented a symmetric NMF variant that solves $\hat{\mathrm{A}}\approx H{H}^{{\rm{T}}}$ via a multiplicative update with column renormalization in each step to prevent column collapse to zero^76^: $H\leftarrow H\odot \frac{{\hat{\mathrm{A}}{H}}}{{{HH}}^{{\rm{T}}}H+\varepsilon }$, producing a single niche-membership matrix H.

### Interpretation

The weights of the k factors in $S\in {{{{\mathbb{R}}}}}^{t\times k}$ over t cell types can be interpreted as membership weights of cell types in clusters, or ‘topics’ (motifs) of frequently colocalizing cell types. Similar to the application of NMF in document text analysis and other tasks^77,78^, cell types fall into clusters, because since W and H are non-negative, the columns of S are additive and cannot cancel each other, forcing each NMF component to explain a distinct, non-overlapping portion of the observed colocalization signal—while optimizing the low-rank approximation of the original cell-type interaction matrix incentivizes the capture of frequent motifs^79^. Thus, nichefinder yields a soft-clustering (the matrix S) of cell types into colocalization motifs, where each cell type can be part of multiple motifs, allowing to capture nuanced participation patterns (for example the same immune cell type in different microenvironments), not forcing cell types to ‘pick’ a cluster. Such global cell-type colocalization motifs can be interpreted as cell-type niches depending on which aspects to highlight in the definition of a niche: nichefinder motifs are likely to match microenvironments that are characterized by cell type composition and re-occur across space. Thus, nichefinder focuses on distinguishing global archetypical interaction patterns from noise and background distributions. However, rare structurally characterized microenvironments (for example, a small diseased area) or microenvironments characterized by molecule concentrations (such as secreted ligands) may be better identified with other respectively specialized methods.

The cell-type to niche assignments identified by nichefinder can be visualized as a bipartite graph for an abstract representation of cell-type interactions bundled as niches. Similar to PAGA, a threshold on the weights in the cell-type by niche membership matrix can be applied to obtain a decluttered visualisation. This approach facilitates the interpretation of spatial microenvironment complexity, revealing both distinct and overlapping cell type contributions to each niche within the analysed tissue.

### Comparison with other methods

Most existing tools frame niche detection as a problem of labelling each individual cell with a categorical niche label. By contrast, nichefinder defines niches as groups of cell types (not cells) that are in close spatial proximity. If annotating spatially contiguous domains is not required, focusing on cell-type-level interactions rather than labelling individual cells circumvents dealing with noise on the single-cell level and is easier to achieve without the use of complex modelling, simplifying the interpretation of results. In addition, not assuming spatial contiguity may be more suitable for analysing interactions of more dispersed cell types such as immune cells or smaller blood vessels. Niche detection in nichefinder is mainly achieved by direct quantification through aggregating the spatial *k*-NN graph, similar to how PAGA works, and subsequent clustering of interactions into niches. For example, high-complexity algorithms include NicheCompass^80^, SpaGCN^81^ and GraphST^82^ and low-complexity cell-level niche assignment workflows include Seurat neighbourhood *k*-means^83^. For interaction analysis, high-complexity workflows include NiCO^84^, SpaceMarkers^85^ and Niche-DE^86^ and low-complexity methods include Nichefinder, as presented here.

Published methods that focus on cell-type interactions rather than niche assignments of individual cells add further complexity to the analysis by also modelling gene expression and ligand–receptor interactions. By contrast, we developed nichefinder as a low-complexity modular pipeline of simple consecutive and interchangeable steps, separating the use of gene expression profiles (step 1, see above) and spatial information (steps 2–3) for better interpretability. For all the steps, we chose methods that are well established in single-cell and spatial transcriptomics analyses (logistic regression^16^, *k*-NN graph aggregation^87^ and NMF, respectively). Importantly, the last step only adds extra interpretation of the cell-type interaction matrix in the form of niche assignments.

As nichefinder approaches niche analysis in a unique way, direct comparison with published methods is difficult. NiCO not only uses gene expression profiles for interaction analysis but also focuses on cell-type interactions. We therefore, benchmarked nichefinder against NiCO on simulated data from the NiCO paper (Extended Data Fig. 7b–i). The cell-type interactions detected by nichefinder and NiCO are nearly identical, with high correlations between them for two different simulated scenarios (0.86 and 0.82; Extended Data Fig. 7d,h), thereby validating the nichefinder approach. In addition, NMF-derived niches add another level of interpretation.

Although difficult to benchmark, we aimed at comparing the nichefinder approach with cell-level niche assignments, using NicheCompass as an example (Extended Data Fig. 7j–p). Applied to mouse hippocampus data^88^, NicheCompass clearly annotates broad spatial domains (Extended Data Fig. 7k). To be able to compare the methods, we mapped nichefinder niche memberships back to cells using the cell-type probability matrix (Extended Data Fig. 7i). Compared with NicheCompass, the nichefinder niches are more dispersed and resemble the original cell-type label distribution more closely. When niche labels are mapped onto individual cell neighbourhoods (a cell and its *k*-NN) using a weighted (using niche-membership weights) geometric mean to ensure all cell types in a niche are represented, patterns of individual niches can be highlighted well (Extended Data Fig. 7m–o). This includes a relatively dispersed vascular niche 0, a localized niche 2 consisting of mostly ependymal cells and niche 10, a mix of ependymal cells and endothelial tip cells. When clustering Nichecompass and nichefinder niches together on the basis of their cell-type memberships (Extended Data Fig. 7p), a matching nichefinder niche can be found for at least half of the Nichecompass niches. Unsurprisingly, a block of NicheCompass niches that cannot be identified from the cell-type labels (0, 1, 5, 11 and 14), but map to structural domains^80^, are not distinguished by nichefinder. By contrast, nichefinder focuses more niches on dispersed cell types such as astrocytes, MG and ECs, detecting more informative patterns for them (Extended Data Fig. 7m) compared with the broad localized areas obtained from NicheCompas (Extended Data Fig. 7k).

### Gene set enrichment analysis for angiogenesis

For analysis of angiogenesis pathway-associated gene expression within the BAM populations, we obtained the Gene Ontology biological process 2023 sprouting angiogenesis geneset (GO:0002040). For the cell states of interest, the scanpy function sc.tl.score_genes was utilized to compute gene expression scores for the gene set.

### Cell–cell interactions

Ligand–receptor interactions were inferred using ‘cpdb_analysis_method.call’ in CellPhoneDB (v.4.0.0)^89^. We included genes expressed in more than 10% of cells within each cluster. Inferred interactions with a *P* > 0.001 were removed.

### Statistics and reproducibility

No statistical methods were used to predetermine sample sizes, but our sample sizes are similar to those reported in previous publications^57^. No data were excluded from the analyses, and data distribution was assumed to be normal; however, this was not formally tested. The experiments were not randomized. The investigators were not blinded to allocation during experiments and outcome assessment.

### Reporting summary

Further information on research design is available in the Nature Portfolio Reporting Summary linked to this article.

## Online content

Any methods, additional references, Nature Portfolio reporting summaries, source data, extended data, supplementary information, acknowledgements, peer review information; details of author contributions and competing interests; and statements of data and code availability are available at https://doi.org/10.1038/s41556-026-02075-8.

## Supplementary information

Reporting Summary

Peer Review File

Supplementary Table 1 Droplet dataset sample-level metadata. Metadata of each donor, including regions from which they were sampled (anterior, falcine, posterior and not available), the ploidy status, sex and PCW stage.

Supplementary Table 2 Y chromosome gene set. Genes of the Y chromosome, used in gene set enrichment analysis to confirm sample sex.

Supplementary Table 3 Anterior-region-enriched pathways. Gene set enrichment analysis results for terms within WikiPathway 2024 biological pathways.

Supplementary Table 4 Spatial dataset sample-level metadata. Metadata of each donor, region and PCW stage.

## Data Availability

Data that support the findings of this study, including processed snRNA–scATAC sequencing and in situ sequencing (10x Xenium), are available at https://developmental.cellatlas.io/meninges-development. Raw sequencing data are accessible via ArrayExpress under accession number E-MTAB-17337. Previously published data re-analysed here are cited in the relevant sections. Data from Suo et al.^10^ are accessible at https://developmental.cellatlas.io/fetal-immune (count matrices) and ArrayExpress, under E-MTAB-11343 (raw data). Data from Vinsland et al. ^27^ are available via GitHub at https://github.com/linnarsson-lab/human-meninges-development (count matrices), and data from Goh et al. are available at https://developmental.cellatlas.io/yolk-sac (ref. ^44^) (count matrices) and from ArrayExpress under E-MTAB-11673 (raw data). Source data for Figs. 1–5 and Extended Data Figs. 1–6, including processed count matrices, are available via Zenodo at https://doi.org/10.5281/zenodo.21642652 (ref. ^90^). All other data supporting the findings of this study are available from the corresponding author on reasonable request.

## Code availability

Custom code utilized in this study is available via GitHub at https://github.com/Teichlab/nichefinder and https://github.com/Teichlab/cell2home, as well as https://cell2home.readthedocs.io/en/latest/.
