# Supplementary material for: Spatially resolved multiomics of human meningeal development reveal lineage and disease dynamics
Source: Nat Cell Biol. 2026 Sep 28;28(10):2169–78. doi: 10.1038/s41556-026-02075-8 (PMC13661283; doi:10.1038/s41556-026-02075-8)
Supplement: Supplementary file 1 [file 41556_2026_2075_MOESM1_ESM.pdf]

nature portfolio

Corresponding author(s): Sarah Teichmann

Last updated by author(s): Jul 9, 2026

Reporting Summary

Nature Portfolio wishes to improve the reproducibility of the work that we publish. This form provides structure for consistency and transparency in reporting. For further information on Nature Portfolio policies, see our Editorial Policies and the Editorial Policy Checklist.

Statistics

For all statistical analyses, confirm that the following items are present in the figure legend, table legend, main text, or Methods section.

n/a	Confirmed	
☒	☐	The exact sample size (*n*) for each experimental group/condition, given as a discrete number and unit of measurement
☒	☐	A statement on whether measurements were taken from distinct samples or whether the same sample was measured repeatedly
☐	☒	The statistical test(s) used AND whether they are one- or two-sided<br>*Only common tests should be described solely by name; describe more complex techniques in the Methods section.*
☐	☒	A description of all covariates tested
☐	☒	A description of any assumptions or corrections, such as tests of normality and adjustment for multiple comparisons
☒	☐	A full description of the statistical parameters including central tendency (e.g. means) or other basic estimates (e.g. regression coefficient) AND variation (e.g. standard deviation) or associated estimates of uncertainty (e.g. confidence intervals)
☒	☐	For null hypothesis testing, the test statistic (e.g. *F*, *t*, *r*) with confidence intervals, effect sizes, degrees of freedom and *P* value noted<br>*Give P values as exact values whenever suitable.*
☒	☐	For Bayesian analysis, information on the choice of priors and Markov chain Monte Carlo settings
☒	☐	For hierarchical and complex designs, identification of the appropriate level for tests and full reporting of outcomes
☒	☐	Estimates of effect sizes (e.g. Cohen's *d*, Pearson's *r*), indicating how they were calculated

Our web collection on statistics for biologists contains articles on many of the points above.

Software and code

Policy information about availability of computer code

Data collection	no software was used for data collection
Data analysis	Cell2home version 1, and Nichefinder version 1 were custom code developepd within this manuscript, used for data analysis

For manuscripts utilizing custom algorithms or software that are central to the research but not yet described in published literature, software must be made available to editors and reviewers. We strongly encourage code deposition in a community repository (e.g. GitHub). See the Nature Portfolio guidelines for submitting code & software for further information.

Data

Policy information about availability of data

All manuscripts must include a data availability statement. This statement should provide the following information, where applicable:

- Accession codes, unique identifiers, or web links for publicly available datasets
- A description of any restrictions on data availability
- For clinical datasets or third party data, please ensure that the statement adheres to our policy

Processed snRNA–scATAC-seq, ISS data will be available for browsing and download from https://developmental.cellatlas.io/meninges-development upon publication.

Research involving human participants, their data, or biological material

Policy information about studies with human participants or human data. See also policy information about sex, gender (identity/presentation), and sexual orientation and race, ethnicity and racism.

Reporting on sex and gender	We do not focus on sex-specific analyses within this cell atlas data resource publication.<br>Sex information for each sample was determined through karyotyping data obtained through the Human Developmental Bioresource (HDBR) for samples within the second trimester. For first-trimester samples, Y chromosome gene-expression (or their absence of), was used to determine sex per sample.
Reporting on race, ethnicity, or other socially relevant groupings	We do not report on this within this cell atlas data resource publication.<br>This information is not routinely collected within the HDBR for embryonic samples.
Population characteristics	See above
Recruitment	as above, via the HDBR.
Ethics oversight	First trimester (< 12 PCW) meninges samples were obtained from elective terminations under REC 96/085 with written and informed consent obtained from all sample donors (East of England, with full approval from the Cambridge Central Research Ethics Committee).<br>Second trimester tissues (> 12 PCW) were obtained from the joint MRC/Wellcome Trust Human Developmental Biology Resource (http://hdbr.org; grant number MR/R006237/1) at the Institute of Child Health, London (Registration number 200683) under full ethical approval from the London-Fulham Research Ethics Committee. London Fulham Research Ethics Committee (18/LO/0822 IRAS 326492), (second trimester samples are additionally covered by bpas (British Pregnancy Advisory Service) REC Ref 2008/05/ HDBR

Note that full information on the approval of the study protocol must also be provided in the manuscript.

Field-specific reporting

Please select the one below that is the best fit for your research. If you are not sure, read the appropriate sections before making your selection.

☒ Life sciences ☐ Behavioural & social sciences ☐ Ecological, evolutionary & environmental sciences

For a reference copy of the document with all sections, see nature.com/documents/nr-reporting-summary-flat.pdf

Life sciences study design

All studies must disclose on these points even when the disclosure is negative.

Sample size	Sample sizes were chosen based on previous published work using cell atlasing technologies to profile developmental samples across age. No formal power calculations were performed prior to sample collection
Data exclusions	NA
Replication	NA
Randomization	NA
Blinding	NA

Reporting for specific materials, systems and methods

We require information from authors about some types of materials, experimental systems and methods used in many studies. Here, indicate whether each material, system or method listed is relevant to your study. If you are not sure if a list item applies to your research, read the appropriate section before selecting a response.

Materials & experimental systems

n/a	Involved in the study
☐	☒ Antibodies
☒	☐ Eukaryotic cell lines
☒	☐ Palaeontology and archaeology
☒	☐ Animals and other organisms
☒	☐ Clinical data
☒	☐ Dual use research of concern
☒	☐ Plants

Methods

n/a	Involved in the study
☒	☐ ChIP-seq
☒	☐ Flow cytometry
☒	☐ MRI-based neuroimaging

Antibodies

Antibodies used

The following antibodies were utilised: CD31 (EPR5251, Abcam, 2.0 ug/mL, Lot:210910), CD20 (L26, eBioscience, 1.0ug/mL, Lot: 210429), CD163 (EPR14647, Abcam, 2.0 ug/ml, Lot:210910), Vimentin (091d3, Biolegend, 2.0ug/mL, Lot:3), ACTA2 (1A4, Rarecyte in-house).

Validation

as manufacturer

Plants

Seed stocks

NA

Novel plant genotypes

NA

Authentication

NA
