# Supplementary material for: Spatially resolved multiomics of human meningeal development reveal lineage and disease dynamics
Source: Nat Cell Biol. 2026 Sep 28;28(10):2169–78. doi: 10.1038/s41556-026-02075-8 (PMC13661283; doi:10.1038/s41556-026-02075-8)
Supplement: Supplementary file 2 [file 41556_2026_2075_MOESM2_ESM.pdf]

nature portfolio

Peer Review File

Spatially resolved multiomics of human meninges development reveal lineage and disease dynamics

Corresponding Author: Professor Sarah Teichmann

Version 0:

Decision Letter:

*Please delete the link to your author homepage if you wish to forward this email to co-authors.

Dear Sarah,

Your manuscript, "Spatially resolved multiomics of human meninges development reveal lineage and disease dynamics", has now been seen by 4 referees: an expert in (human) brain development (referee 1); an expert in single-cell and spatial transcriptomics, chromatin analysis, cancer, computational biology (referee 2); referee 3, who is an early career researcher and co-reviewed the manuscript together with referee 2; and an expert in brain tumors, meningioma (referee 4). As you will see from their comments (attached below) they find this work of potential interest, but have raised substantial concerns, which in our view would need to be addressed with considerable revisions before we can consider publication in Nature Cell Biology.

Nature Cell Biology editors discuss the referee reports in detail within the editorial team, including the chief editor, to identify key referee points that should be addressed with priority, and requests that are overruled as being beyond the scope of the current study. To guide the scope of the revisions, I have listed these points below. I should stress that the referees' concerns point to a premature dataset and these points would need to be addressed with experiments and data, and reconsideration of the study for this journal and re-engagement of referees would depend on strength of these revisions.

In particular, it would be essential to:

(A) Ensure to provide full access to all raw data and code, both of which will need to be assessed thoroughly by the reviewers.

Reviewer #2 says:
"Neither codes nor data are available to test"

Reviewer #3 says:
"The authors provided the link https://github.com/Teichlab/cell2homes as the location for the code, but the link is not working."

(B) Reviewer #4 indicates multiple and serious issues regarding the meningioma analyses and data. We do value this part of the paper and we think it is a compelling addition, but after careful consideration and discussion within the editorial team, we have decided that the manuscript would be of interest to us even in the absence of these meningioma data. Therefore, and per the reviewer's comments, we request that you either completely remove any data, analyses, figures and claims or relevant discussion pertaining to the meningioma part of the study *or* (if that is what you prefer) you substantially revise the manuscript regarding this part, per reviewer #4's instructions.

(C) Address all the issues raised by reviewers #1, #2 and #3 in terms of providing thorough reporting and relevant analyses, as well as clarifications and addressing discrepancy and reproducibility points to flesh out the value of the dataset.

Reviewer #1 says:

"Sex information is absent from Figure 1b and supplementary data for each sample. Given that each developmental age is represented by a single time point, sex represents an uncontrolled confound for gene expression differences. The authors should provide sex information for all samples and, where possible (e.g., at 14 PCW where both Xenium and multiome data exist), test whether samples are from the same or different sexes and assess potential sex effects."

"Regional information is inadequately presented throughout. The UMAP in Figure 1c lacks annotation for sample age and region, and this information is not provided in supplementary data. It is unclear whether regions from the same age were pooled, whether all regions were collected from all ages, and how regional differences were analyzed. Spatial data throughout the manuscript similarly lacks regional annotation. Given known developmental differences in regional origins of meninges, this represents a significant analytical gap that

undermines the "spatially resolved" claim in the title."

"Nuclei numbers per age/sample are not reported, making it impossible to assess whether any ages are over- or under-represented in the analyses which may influence data interpretation."

"Time/developmental age is inconsistently represented across analyses. While shown in Figure 2c and Extended Figure 3, it is absent from many critical figures (e.g., Figure 1c UMAP, Figure 4b UMAP, Figure 4i spatial data), making it difficult to evaluate developmental trajectories and assess whether conclusions are fully supported by the data."

Reviewers #2 and #3 say:

"This resource paper included samples from 17 donors, from 6 to 21 PCB. However, clarification is needed on donor details. More detailed characteristics and potential confounding factors may affect the future analysis and interpretation of these valuable data."

"Authors need to provide detailed information on the quality control steps for these dataset, as well as the following computational analysis, how to ensure the robustness and reproducibility and mitigate the batch effects. It is disappointing that certain inconsistencies in the results are explained simply as “sample error.” This raises concerns regarding sample handling, quality control, and analytical robustness. For a Resource paper intended to serve as a foundational dataset, the authors should more rigorously investigate discrepancies across donors, perform additional validation, or clearly document how such sample-level anomalies were identified and addressed. Transparent resolution rather than attributing deviations to unspecified sample error is crucial for maintaining confidence in the dataset’s reliability. Moreover, it will be interesting to compare the newly generated data with open-source data to check the consistency. Though no study has integrated data, but there are different studies with single platform data publicly shared."

"The study demonstrated the interesting insights into T21 impact on meningeal development..." and "... the potential confounding factors, such as maternal health, gestational age, genetic background of donors are not sufficiently addressed."

(D) Address the issues indicated by reviewers #2 and #3 regarding Nichefinder:

"Nichefinder, a new tool proposed to study fibroblast populations in meningeal tissues, is not benchmarked with other spatial mapping and cluster algorithms. The lack of such benchmarking raises concerns about its robustness and generalizability. The inclusion of Nichefinder in this resource paper without validation diminishes the rigor, can lead to potential biases in interpreting biology, especially in draft serve as foundational resource."

"Lines752-755: The authors mentioned “we subset genes for model training to highly variable genes shared between the two datasets. Next, we construct a cell-cell connectivity graph using the k-nearest neighbors (k-NN) algorithm to identify spatial proximity between individual cells.” The description of the Nichefinder pipeline lacks key details."

"How are HVGs selected in Nichefinder? Does varying the number of HVGs produce stable niche structures, or do niche assignments change substantially?"

(E) Address the issue raised by reviewers #2 and #3 regarding the Cell2home algorithm. The algorithm should either be incorporated into the present manuscript, together with all necessary details, so that it can be properly presented and assessed by the reviewers *or* you should follow the reviewers' suggestion and use a different and published algorithm instead:

"Lines 717-730 (cell migration analysis): Cell2home algorithm presented in this section is not yet published (Dufva et al., in preparation) and not in the reference list. Description of the algorithm provided in this section also does not clearly explain how the algorithms work. I will suggest the authors to consider using a peer-reviewed, validated algorithm."

(F) All other referee concerns pertaining to strengthening existing data, providing clarifications, textual and presentation corrections, should also be addressed.

(G) Finally, please pay close attention to our guidelines on statistical and methodological reporting (listed below) as failure to do so may delay the reconsideration of the revised manuscript. In particular please provide:

- unprocessed images of all gels/blots in the form of a multi-page pdf file. Please ensure that blots/gels are labeled and the sections presented in the figures are clearly indicated.

- all numerical source data in Excel format, with data for different figures provided as different sheets within a single Excel file. The file should include source data giving rise to graphical representations and statistical descriptions in the paper and for all instances where the figures present representative experiments of multiple independent repeats, the source data of all repeats should be provided.

We would be happy to consider a revised manuscript that would satisfactorily address these points.

On the contrary, reviewers #2 and #3 indicated in terms of the T21 data: "Moreover, the disease meachnisam is underexplored". While we acknowledge the validity of this request, we have decided that addressing it with new data and analyses would be beyond the scope of the current study, which we are considering as a Resource. We therefore do not expect you to address this comment with new experiments and data. We would appreciate it if you could briefly acknowledge this in the discussion as a limitation and future direction.

When revising the manuscript please:

- ensure that it conforms to our format instructions and publication policies (see below and www.nature.com/nature/authors/).

- provide a point-by-point rebuttal to the full referee reports verbatim, as provided at the end of this letter.

- provide the completed Reporting Summary (found here https://www.nature.com/authors/policies/ReportingSummary.pdf). This is essential for reconsideration of the manuscript and this document will be available to editors and referees in the event of peer review. For more information see http://www.nature.com/authors/policies/availability.html or contact me.

Nature Cell Biology is committed to improving transparency in authorship. As part of our efforts in this direction, we are now requesting that all authors identified as ‘corresponding author’ on published papers create and link their Open Researcher and Contributor Identifier (ORCID) with their account on the Manuscript Tracking System (MTS), prior to acceptance. ORCID helps the scientific community achieve unambiguous attribution of all scholarly contributions. You can create and link your ORCID from the home page of the MTS by clicking on ‘Modify my Springer Nature account’. For more information please visit please visit www.springernature.com/orcid.

Please submit the revised manuscript files and the point-by-point rebuttal to the referee comments using this link:

Link Redacted

*This url links to your confidential home page and associated information about manuscripts you may have submitted or be reviewing for us. If you wish to forward this email to co-authors, please delete the link to your homepage.

We would like to receive a revised submission within six months. We would be happy to consider a revision even after this timeframe, however if the resubmission deadline is missed and the paper is eventually published, the submission date will be the date when the revised manuscript was received.

We hope that you will find our referees' comments, and editorial guidance helpful. Please do not hesitate to contact me if there is anything you would like to discuss.

Best wishes,

Stelios

Dr. Stylianos Lefkopoulos
He/him/his
Senior Editor, Nature Cell Biology
Springer Nature
Heidelberger Platz 3, 14197 Berlin, Germany

E-mail: stylianos.lefkopoulos@springernature.com
Bluesky: @slefkopoulos.bsky.social
LinkedIn: www.linkedin.com/in/stylianos-lefkopoulos-81b007a0

Reviewers' Comments:

Reviewer #1 (Remarks to the Author):

This manuscript presents comprehensive single-nucleus multiome n=10 datasets from human meninges spanning 6.7-21 weeks post-conception. These are complemented by Xenium spatial transcriptomic datasets from four individuals at distinct developmental stages and three trisomy 21 (T21) cases for a total of 17 donors. The authors analyze four primary meningeal lineages—endothelial, immune, and mesenchymal populations—comparing developmental profiles to T21 and adult meningioma samples. A novel computational pipeline, Nichefinder, is introduced for Xenium data segmentation.

Key findings include:
- depletion of stromal and erythroid cells with altered gene expression profiles in T21;
- identification of Pre-MACCs as potential CNS-resident microglia/border-associated macrophage progenitors until late second trimester;
- demonstration of the meninges as a critical site for embryonic and fetal B-cell lineage development;
- identification of similarities between fetal dural fibroblast states and meningiomas, particularly Dural-FIB1 signatures.

This work represents a valuable contribution to developmental neuroscience, providing rare and comprehensive datasets that will serve as an important resource for the community. The integration of single-nucleus multiome and spatial transcriptomic approaches across developmental time points is state of the art. The findings regarding meningeal B-cell development and Pre-MACC identity as potential progenitors are particularly significant and advance our understanding of meningeal biology. The comparative analysis with meningiomas provides preliminary insights into potential cells of origin for these tumors.

The experimental approach is state-of-the-art and technically sound. The statistical approaches employed are appropriate for the analyses presented. Nichefinder is a new important tool.

However, there are significant concerns regarding data completeness and presentation that limit the interpretability of results and must be addressed:

1. Sex information is absent from Figure 1b and supplementary data for each sample. Given that each developmental age is represented by a single time point, sex represents an uncontrolled confound for gene expression differences. The authors should provide sex information for all samples and, where possible (e.g., at 14 PCW where both Xenium and multiome data exist), test whether samples are from the same or different sexes and assess potential sex effects.

2. Regional information is inadequately presented throughout. The UMAP in Figure 1c lacks annotation for sample age and region, and this information is not provided in supplementary data. It is unclear whether regions from the same age were pooled, whether all regions were collected from all ages, and how regional differences were analyzed. Spatial data throughout the manuscript similarly lacks regional annotation. Given known developmental differences in regional origins of meninges, this represents a significant analytical gap that undermines the "spatially resolved" claim in the title.

3. Nuclei numbers per age/sample are not reported, making it impossible to assess whether any ages are over- or under-represented in the analyses which may influence data interpretation.

4. The rationale for why the 12 PCW sample was unsorted is unclear. I can find no sorting information in the Methods section

5. Time/developmental age is inconsistently represented across analyses. While shown in Figure 2c and Extended Figure 3, it is absent from many critical figures (e.g., Figure 1c UMAP, Figure 4b UMAP, Figure 4i spatial data), making it difficult to evaluate developmental trajectories and assess whether conclusions are fully supported by the data.

6. Several conclusions are well-supported by the data, particularly those regarding B-cell lineage development and Pre-MACC identity. However, some conclusions are either inadequately supported or overly speculative. Particularly problematics is the link hypothesized between MTORC1 pathway enrichment in the T21 immune compartment and downstream effects on Alzheimer's disease progression. This is inappropriate and should be removed entirely or substantially revised and supported by extensive functional data r. Also these hypotheses should be in the discussion section not the results.

7. Minor issue Figure 4 requires correction: panel l description is missing from the legend, and panel m is mislabeled as panel l

Overall Assessment
This manuscript presents valuable datasets and several important findings that advance our understanding of meningeal development. With the revisions, this work would represent an important contribution to the field and a valuable resource for the community.

Reviewer #2 (Remarks to the Author):

The authors aimed to build a multi-omics atlas of human meningeal development from 6 to21 post-conception weeks (PCW) by using paired snRNA-seq and snATAC-seq data, spatial transcriptomics. They studied the developmental trajectory of meningeal fibroblast, immune cell (B cell), and endothelial cells, and explore how trisomy 21 affect meningeal development. This study provides a comprehensive resource for studying both normal development and disease dynamics.

The work contained several strengths, including comprehensive data paired with advanced technology (single cell and spatial), as well as demonstrated new insight on meningeal immune ecology and T21.

There are several directions to improve:

1. This resource paper included samples from 17 donors, from 6 to 21 PCB. However, clarification is needed on donor details. More detailed characteristics and potential confounding factors may affect the future analysis and interpretation of these valuable data.

2. Authors need to provide detailed information on the quality control steps for these dataset, as well as the following computational analysis, how to ensure the robustness and reproducibility and mitigate the batch effects. It is disappointing that certain inconsistencies in the results are explained simply as “sample error.” This raises concerns regarding sample handling, quality control, and analytical robustness. For a Resource paper intended to serve as a foundational dataset, the authors should more rigorously investigate discrepancies across donors, perform additional validation, or clearly document how such sample-level anomalies were identified and addressed. Transparent resolution rather than attributing deviations to unspecified sample error is crucial for maintaining confidence in the dataset’s reliability. Moreover, it will be interesting to compare the newly generated data with open-source data to check the consistency. Though no study has integrated data, but there are different studies with single platform data publicly shared.
3. Nichefinder, a new tool proposed to study fibroblast populations in meningeal tissues, is not benchmarked with other spatial mapping and cluster algorithms. The lack of such benchmarking raises concerns about its robustness and generalizability. The inclusion of Nichefinder in this resource paper without validation diminishes the rigor, can lead to potential biases in interpreting biology, especially in draft serve as foundational resource.
4. The study demonstrated the interesting insights into T21 impact on meningeal development, however, the sample size is limited. Moreover, the disease meachnisam is underexplored. Also, the potential confounding factors, such as maternal health, gestational age, genetic background of donors are not sufficiently addressed.

Below are the comments from an early career researcher co-reviewed this paper:

This manuscript presents a paired snRNA-seq and snATAC-seq atlas of human meningeal development, integrates these data with spatial transcriptomics using a newly introduced algorithm (Nichefinder), and investigates key stromal, endothelial, and fibroblast populations. While the study offers valuable insights into meningeal development, several methodological clarifications are needed.
• Line 137: Please clarify how the label transfer on the integrated scRNA-seq atlas was performed.
• Line 140: The manuscript references “Extended Data Fig. a,e,” which is unclear. Please specify exactly which Extended Data Figures you are referring to.
• Why was Pia_FIB1 assigned the earliest pseudotime values in Extended Data Fig. 3? Do you predefined Pia_FIB1 to be the start of the trajectory and if so any biological justification for that?
• Lines 204-207: The authors cite “Supplementary Fig. 2,” which does not exist. Do you mean Extended Data Fig. 6? Please correct this.
• Please add a description of the stages depicted in the left panel to Fig. 3a legend.
• Lines 259-260: Please clarify what metadata PCA was performed on. Explain how PCA was applied to a metadata table containing both numerical and categorical variables, did you perform any preprocessing?
• Lines 269-270: The correlation between chemokine genes and NF-κB downregulation is unclear. Please provide a description at least in the Methods section of how this correlation analysis was performed.
• Consider revising Fig. 4d to display the actual Euclidean distance values on the axis for transparent reporting.
• The purpose of the dashed lines in Fig. 4g-h is unclear. If they indicate niche boundaries, please describe how the boundaries are defined on these networks. What criteria determine the end of the vascular niche, for example? Also, please correct the description of Fig 4g. in the figure legend.
• Lines 322-323: Here, how do you decide on the root of the trajectory (Pre_MACs)? Please provide the biological rationale for selecting this as the start of the trajectory.
• Lines 356-358: Please list the top enriched biological processes and report these results atleast in the Supplementary Materials.
• Fig. 6h appears not to be cited in the manuscript. Please review and add the appropriate citation.
• Lines 717-730 (cell migration analysis): Cell2home algorithm presented in this section is not yet published (Dufva et al., in preparation) and not in the reference list. Description of the algorithm provided in this section also does not clearly explain how the algorithms work. I will suggest the authors to consider using a peer-reviewed, validated algorithm.
• Lines752-755: The authors mentioned “we subset genes for model training to highly variable genes shared between the two datasets. Next, we construct a cell-cell connectivity graph using the k-nearest neighbors (k-NN) algorithm to identify spatial proximity between individual cells.” The description of the Nichefinder pipeline lacks key details.
- How many genes were selected as HVGs, and how were these determined?
- How many nearest neighbors (k) were used in the k-NN graph?
- Does the choice of nearest neighbor (k) influence the number or composition of detected niches?
• The manuscript states that “The soft-clustering property of NMF allows cell types to associate with multiple niches, capturing nuanced participation patterns across the tissue microenvironment.” Please elaborate on how this is achieved in practice, i.e., associating celltype with multiple niches. Additionally, please show niche structures directly on the tissue images prior to the network representation. This will allow readers to clearly see the niche structures and what celltypes are within each niche.
• Lines 774-778: How is the niche membership score computed? Please describe this and how the threshold was chosen.
• How are HVGs selected in Nichefinder? Does varying the number of HVGs produce stable niche structures, or do niche assignments change substantially?

Reviewer #2 (Remarks on code availability):

Neither codes nor data are available to test

Reviewer #3 (Remarks to the Author):

I co-reviewed this manuscript with one of the reviewers who provided the listed reports. This is part of the Nature Cell Biology initiative to facilitate training in peer review and to provide appropriate recognition for Early Career Researchers who co-review manuscripts.

Reviewer #3 (Remarks on code availability):

The authors provided the link https://github.com/Teichlab/cell2homes as the location for the code, but the link is not working.

Reviewer #4 (Remarks to the Author):

This manuscript by To et al presents an impressive developmental atlas of meningeal cell development in humans at the single cell level. Using single cell sequencing and single cell ATAC seq the authors have taken meninges at several time points during embryonic development and investigated the immune environment and cytokines, mesenchymal fibroblastic cells, blood brain barrier biology, signaling through several pathways including JAK/STAT and mTOR to connect this data with multiple disease states including Alzheimer's disease and trisomy 21. They finally look at parallels between fibroblasts at various embryonic states and meningiomas, a tumor that arises from the meninges. The data is an extension of similar published work done in mouse meningeal development, and extensive analysis of human meningioma.

Having such a dataset available, and the biology of the developing meninges explained at the single cell level would be highly advantageous for the research community. Further, I would suspect that this work would be highly cited by future papers investigating details of this very interesting developmental system.

I am not prepared to comment on the developmental aspects of the work, although it seems well done. I do wonder about the breadth of some of the claims regarding connections between meningeal development and some of the diseases that that are connected to it in this paper. However others should comment on this.

What I am prepared to say is that the data in figure 6 connecting development of the meninges and meningioma is not necessary in this paper, and if it were to be included, should be better thought through and analyzed.

1) The single cell data on meningiomas that is used here is from two published datasets (fig 6a). But the WHO grade and genetic background (ch22 loss, ch1 loss, ch10 loss, ch14 loss etc) of these tumors is not noted in this paper. For example, which of them are NF2 mutant, vs NF2 wildtype and mutant for other genes such as Akt, Smo, KLF4 etc? this matters because the biology of these tumors is different, especially given the discussion of the similarities between meningeal development and NF2 function. Yet in 6b, all the tumors have the most similarity to duraf1b1 fibroblasts, regardless of WHO grade or genetic background.

2) In 6c, the data is comforting in that the more aggressive tumors are more similar to the earlier fibroblasts. But what are the statistics here? Are there enough independent tumors of these grades to know this for sure (seems unlikely). And what are the genes that are driving this?

3) In 6d, there are a series of genes presented that connect WHO1, 2, and 3 with fibroblast developmental stages, but less than 10. Is that significant given the number of tumors of each grade and genetic background. I suspect that significantly more tumors would need to be analyzed by single cell analysis and included in this analysis to be able to make claims about these (less than 10) genes.

4) And the expression of these genes and the ones in 6g that are claimed to be "meningioma genes" (whatever that means) in published bulk RNA seq of large numbers of meningiomas (not referenced in this paper), is not what one would expect. Although some of these genes such as Max, and Robo1 are regulated across the population, the trend is not always in the direction that makes sense. In most cases the expression levels do not correlate with WHO grade or genetic background (which is seen for NF2 and to some degree SMARCB1). Which seems to go against the general claims of this part of the paper.

5) To be clear, the idea of connecting meningeal development to meningioma formation is very compelling. I just don't think that this data does that. And if I am wrong, there needs to be much more clarity in the story for this section, that could well be its own paper with proper analysis.

GUIDELINES FOR MANUSCRIPT SUBMISSION TO NATURE CELL BIOLOGY

READABILITY OF MANUSCRIPTS – Nature Cell Biology is read by cell biologists from diverse backgrounds, many of whom are not native English speakers. Authors should aim to communicate their findings clearly, explaining technical jargon that might be unfamiliar to non-specialists, and avoiding non-standard abbreviations. Titles and abstracts should concisely communicate the main findings of the study, and the background, rationale, results and conclusions should be clearly explained in the manuscript in a manner accessible to a broad cell biology audience. Nature Cell Biology uses British spelling.

MANUSCRIPT FORMAT – please follow the guidelines listed in our Guide to Authors regarding manuscript formats at Nature Cell Biology.

TITLE – should be no more than 100 characters including spaces, without punctuation and avoiding technical terms, abbreviations, and active verbs..

AUTHOR NAMES – should be given in full.

AUTHOR AFFILIATIONS – should be denoted with numerical superscripts (not symbols) preceding the names. Full addresses should be included, with US states in full and providing zip/post codes. The corresponding author is denoted by: "Correspondence should be addressed to [initials]."

ABSTRACT AND MAIN TEXT – please follow the guidelines that are specific to the format of your manuscript, as listed in our Guide to Authors (http://www.nature.com/ncb/pdf/ncb_gta.pdf) Briefly, Nature Cell Biology Articles, Resources and Technical Reports have 3500 words, including a 150 word abstract, and the main text is subdivided in Introduction, Results, and Discussion sections. Nature Cell Biology Letters have up to 2500 words, including a 180 word introductory paragraph (abstract), and the text is not subdivided in sections.

ACKNOWLEDGEMENTS – should be kept brief. Professional titles and affiliations are unnecessary. Grant numbers can be listed.

AUTHOR CONTRIBUTIONS – must be included after the Acknowledgements, detailing the contributions of each author to the paper (e.g. experimental work, project planning, data analysis etc.). Each author should be listed by his/her initials.

FINANCIAL AND NON-FINANCIAL COMPETING INTERESTS – the authors must include one of three declarations: (1) that they have

no financial and non-financial competing interests; (2) that they have financial and non-financial competing interests; or (3) that they decline to respond, after the Author Contributions section. This statement will be published with the article, and in cases where financial and non-financial competing interests are declared, these will be itemized in a web supplement to the article. For further details please see https://www.nature.com/licenceforms/nrg/competing-interests.pdf.

REFERENCES – are limited to a total of 70 for Articles, Resources, Technical Reports; and 40 for Letters. This includes references in the main text and Methods combined. References must be numbered sequentially as they appear in the main text, tables and figure legends and Methods and must follow the precise style of Nature Cell Biology references. References only cited in the Methods should be numbered consecutively following the last reference cited in the main text. References only associated with Supplementary Information (e.g. in supplementary legends) do not count toward the total reference limit and do not need to be cited in numerical continuity with references in the main text. Only published papers can be cited, and each publication cited should be included in the numbered reference list, which should include the manuscript titles. Footnotes are not permitted.

METHODS – Nature Cell Biology publishes methods online. The methods section should be provided as a separate Word document, which will be copyedited and appended to the manuscript PDF, and incorporated within the HTML format of the paper.

Methods should be written concisely, but should contain all elements necessary to allow interpretation and replication of the results. As a guideline, Methods sections typically do not exceed 3,000 words. The Methods should be divided into subsections listing reagents and techniques. When citing previous methods, accurate references should be provided and any alterations should be noted. Information must be provided about: antibody dilutions, company names, catalogue numbers and clone numbers for monoclonal antibodies; sequences of RNAi and cDNA probes/primers or company names and catalogue numbers if reagents are commercial; cell line names, sources and information on cell line identity and authentication. Animal studies and experiments involving human subjects must be reported in detail, identifying the committees approving the protocols. For studies involving human subjects/samples, a statement must be included confirming that informed consent was obtained. Statistical analyses and information on the reproducibility of experimental results should be provided in a section titled “Statistics and Reproducibility”.

All Nature Cell Biology manuscripts submitted on or after March 21 2016 must include a Data availability statement at the end of the Methods section. For Springer Nature policies on data availability see http://www.nature.com/authors/policies/availability.html; for more information on this particular policy see http://www.nature.com/authors/policies/data/data-availability-statements-data-citations.pdf. The Data availability statement should include:

• Accession codes for primary datasets (generated during the study under consideration and designated as "primary accessions") and secondary datasets (published datasets reanalysed during the study under consideration, designated as "referenced accessions"). For primary accessions data should be made public to coincide with publication of the manuscript. A list of data types for which submission to community-endorsed public repositories is mandated (including sequence, structure, microarray, deep sequencing data) can be found here http://www.nature.com/authors/policies/availability.html#data.

• Unique identifiers (accession codes, DOIs or other unique persistent identifier) and hyperlinks for datasets deposited in an approved repository, but for which data deposition is not mandated (see here for details http://www.nature.com/sdata/data-policies/repositories).

• At a minimum, please include a statement confirming that all relevant data are available from the authors, and/or are included with the manuscript (e.g. as source data or supplementary information), listing which data are included (e.g. by figure panels and data types) and mentioning any restrictions on availability.

• If a dataset has a Digital Object Identifier (DOI) as its unique identifier, we strongly encourage including this in the Reference list and citing the dataset in the Methods.

We recommend that you upload the step-by-step protocols used in this manuscript to protocols.io. More details can found at https://www.protocols.io/help/publish-articles.

DISPLAY ITEMS – main display items are limited to 6-8 main figures and/or main tables for Articles, Resources, Technical Reports; and 5 main figures and/or main tables for Letters. For Supplementary Information see below.

FIGURES – Colour figure publication costs $600 for the first, and $300 for each subsequent colour figure. All panels of a multi-panel figure must be logically connected and arranged as they would appear in the final version. Unnecessary figures and figure panels should be avoided (e.g. data presented in small tables could be stated briefly in the text instead).

All imaging data should be accompanied by scale bars, which should be defined in the legend.
Cropped images of gels/blots are acceptable, but need to be accompanied by size markers, and to retain visible background signal within the linear range (i.e. should not be saturated). The boundaries of panels with low background have to be demarked with black lines. Splicing of panels should only be considered if unavoidable, and must be clearly marked on the figure, and noted in the legend with a statement on whether the samples were obtained and processed simultaneously. Quantitative comparisons between samples on different gels/blots are discouraged; if this is unavoidable, it should only be performed for samples derived from the same experiment with gels/blots were processed in parallel, which needs to be stated in the legend.

Figures should be provided at approximately the size that they are to be printed at (single column is 86 mm, double column is 170 mm) and should not exceed an A4 page (8.5 x 11"). Reduction to the scale that will be used on the page is not necessary, but multi-panel figures should be sized so that the whole figure can be reduced by the same amount at the smallest size at which essential details in each panel are visible. In the interest of our colour-blind readers we ask that you avoid using red and green for contrast in figures. Replacing red with magenta and green with turquoise are two possible colour-safe alternatives. Lines with widths of less than 1 point should be avoided. Sans serif typefaces, such as Helvetica (preferred) or Arial should be used. All text that forms part of a figure should be rewritable and removable.

We accept files from the following graphics packages in either PC or Macintosh format:

- For line art, graphs, charts and schematics we prefer Adobe Illustrator (.AI), Encapsulated PostScript (.EPS) or Portable Document Format (.PDF). Files should be saved or exported as such directly from the application in which they were made, to allow us to restyle them according to our journal house style.

- We accept PowerPoint (.PPT) files if they are fully editable. However, please refrain from adding PowerPoint graphical effects to objects, as this results in them outputting poor quality raster art. Text used for PowerPoint figures should be Helvetica (preferred) or Arial.

- We do not recommend using Adobe Photoshop for designing figures, but we can accept Photoshop generated (.PSD or .TIFF) files only if each element included in the figure (text, labels, pictures, graphs, arrows and scale bars) are on separate layers. All text should be editable in 'type layers' and line-art such as graphs and other simple schematics should be preserved and embedded within 'vector smart objects' - not flattened raster/bitmap graphics.

- Some programs can generate Postscript by 'printing to file' (found in the Print dialogue). If using an application not listed above, save the file in PostScript format or email our Art Editor, Allen Beattie for advice (a.beattie@nature.com).

Regardless of format, all figures must be vector graphic compatible files, not supplied in a flattened raster/bitmap graphics format, but should be fully editable, allowing us to highlight/copy/paste all text and move individual parts of the figures (i.e. arrows, lines, x and y axes, graphs, tick marks, scale bars etc.). The only parts of the figure that should be in pixel raster/bitmap format are photographic images or 3D rendered graphics/complex technical illustrations.

All placed images (i.e. a photo incorporated into a figure) should be on a separate layer and independent from any superimposed scale bars or text. Individual photographic images must be a minimum of 300+ DPI (at actual size) or kept constant from the original picture acquisition and not decreased in resolution post image acquisition. All colour artwork should be RGB format.

FIGURE LEGENDS – must not exceed 350 words for each figure to allow fit on a single printed NCB page together with the figure. They must include a brief title for the whole figure, and short descriptions of each panel with definitions of the symbols used, but without detailing methodology.

TABLES – main tables should be provided as individual Word files, together with a brief title and legend. For supplementary tables see below.

SUPPLEMENTARY INFORMATION – Supplementary information is material directly relevant to the conclusion of a paper, but which cannot be included in the printed version in order to keep the manuscript concise and accessible to the general reader. Supplementary information is an integral part of a Nature Cell Biology publication and should be prepared and presented with as much care as the main display item, but it must not include non-essential data or text, which may be removed at the editor's discretion. All supplementary material is fully peer-reviewed and published online as part of the HTML version of the manuscript. Supplementary Figures and Supplementary Notes are appended at the end of the main PDF of the published manuscript.

Supplementary items should relate to a main text figure, wherever possible, and should be mentioned sequentially in the main manuscript, designated as Supplementary Figure, Table, Video, or Note, and numbered continuously (e.g. Supplementary Figure 1, Supplementary Figure 2, Supplementary Table 1, Supplementary Table 2 etc.).

Unprocessed scans of all key data generated through electrophoretic separation techniques need to be presented in a supplementary figure that should be labelled and numbered as the final supplementary figure, and should be mentioned in every relevant figure legend. This figure does not count towards the total number of figures and is the only figure that can be displayed over multiple pages, but should be provided as a single file, in PDF or TIFF format. Data in this figure can be displayed in a relatively informal style, but size markers and the figures panels corresponding to the presented data must be indicated.

The total number of Supplementary Figures (not including the "unprocessed scans" Supplementary Figure) should not exceed the number of main display items (figures and/or tables (see our Guide to Authors and March 2012 editorial http://www.nature.com/ncb/authors/submit/index.html#suppinfo; http://www.nature.com/ncb/journal/v14/n3/index.html#ed). No restrictions apply to Supplementary Tables or Videos, but we advise authors to be selective in including supplemental data.

Each Supplementary Figure should be provided as a single page and as an individual file in one of our accepted figure formats and should be presented according to our figure guidelines (see above). Supplementary Tables should be provided as individual Excel files. Supplementary Videos should be provided as .avi or .mov files up to 50 MB in size. Supplementary Figures, Tables and Videos much be accompanied by a separate Word document including titles and legends.

GUIDELINES FOR EXPERIMENTAL AND STATISTICAL REPORTING

REPORTING REQUIREMENTS – To improve the quality of methods and statistics reporting in our papers we have recently revised the reporting checklist we introduced in 2013. We are now asking all life sciences authors to complete two items: an Editorial Policy Checklist (found here https://www.nature.com/authors/policies/Policy.pdf) that verifies compliance with all required editorial policies and a reporting summary (found here https://www.nature.com/authors/policies/ReportingSummary.pdf) that collects information on experimental design and reagents. These documents are available to referees to aid the evaluation of the manuscript. Please note that these forms are dynamic 'smart pdfs' and must therefore be downloaded and completed in Adobe Reader. We will then flatten them for ease of use by the reviewers. If you would like to reference the guidance text as you complete the template, please access these flattened versions at http://www.nature.com/authors/policies/availability.html.

STATISTICS – Wherever statistics have been derived the legend needs to provide the n number (i.e. the sample size used to derive statistics) as a precise value (not a range), and define what this value represents. Error bars need to be defined in the legends (e.g. SD, SEM) together with a measure of centre (e.g. mean, median). Box plots need to be defined in terms of minima, maxima, centre, and percentiles. Ranges are more appropriate than standard errors for small data sets. Wherever statistical significance has been derived, precise p values need to be provided and the statistical test used needs to be stated in the legend. Statistics such as error bars must not be derived from n<3. For sample sizes of n<5 please plot the individual data points rather than providing bar graphs. Deriving statistics from technical replicate samples, rather than biological replicates is strongly discouraged. Wherever statistical significance has been derived, precise p values need to be provided and the statistical test stated in the legend.

Information on how many times each experiment was repeated independently with similar results needs to be provided in the legends and/or Methods for all experiments, and in particular wherever representative experiments are shown.

We strongly recommend the presentation of source data for graphical and statistical analyses as a separate Supplementary Table, and request that source data for all independent repeats are provided when representative experiments of multiple independent repeats, or averages of two independent experiments are presented. This supplementary table should be in Excel format, with data for different figures provided as different sheets within a single Excel file. It should be labelled and numbered as one of the supplementary tables, titled "Statistics Source Data", and mentioned in all relevant figure legends.

--------- Please don't hesitate to contact NCB@nature.com should you have queries about any of the above requirements ---------

Version 1:

Decision Letter:

*Please delete the link to your author homepage if you wish to forward this email to co-authors.

Dear Sarah,

Your manuscript, "Spatially resolved multiomics of human meninges development reveal lineage and disease dynamics", has now been seen by the original referees #1, #2 and #3. As you will see from their comments (attached below) they find this work of interest and improved, but reviewer #2 continues to raise some important points. Although we are also very interested in this study, we believe that their concerns should be addressed before we can consider publication in Nature Cell Biology. We would expect you to address all of their comments. Regarding the point about how Cell2home compares to established cell-cell interaction analysis frameworks, we would welcome such data if they exist, but we would be happy to also accept addressing this point only textually.

In addition, while revising your manuscript, please take into account the following formatting guidelines:

(A) Our Resources can have no less than 5 and up to 8 main figures; moreover, they can include up to 10 extended data figures. Your manuscript currently adheres to these guidelines. Please keep in mind, however, that all figures should adhere to a maximum page size of roughly 180mm wide x 200mm high to fit standard page format and use a font size of no smaller than 7pt Arial or Helvetica throughout the figures.

(B) Please ensure to provide all supplementary tables in one single excel workbook, with each table occupying one single tab; if any of your supplementary tables already include more than one tab, then you can provide each supplementary table as a separate excel workbook.

(C) Please ensure that your Abstract does not exceed 150 words.

(D) Please, add the header 'Introduction' right above the respective section.

(E) Please, rename your 'Materials and methods' section to 'Methods.

(F) In the first section of your Methods, in terms of ethics information, please also provide the relevant protocol numbers. Please ensure information on compensation is included. Please ensure that the Reporting Summary also includes both the committees who approved this work and relevant protocol numbers.

(G) Please, do not provide any tables in the Methods section.

(H) Please, include a "Statistics and Reproducibility" section in the Methods with all relevant information in terms of statistics, tests, reproducibility etc

(I) Please refrain from using words such as new/novel/first, when referring to the scientific findings.

(J) References associated with the Methods section should be listed separately at the end of the section (under the header 'Methods-Only References'), and numbered as a continuation of the main manuscript references.

Finally, please pay close attention to our guidelines on statistical and methodological reporting (listed below) as failure to do so may delay the reconsideration of the revised manuscript. In particular please provide:

- unprocessed images of all gels/blots in the form of a multi-page pdf file. Please ensure that blots/gels are labeled and the sections

presented in the figures are clearly indicated.

- all numerical source data in Excel format, with data for different figures provided as different sheets within a single Excel file. The file should include source data giving rise to graphical representations and statistical descriptions in the paper and for all instances where the figures present representative experiments of multiple independent repeats, the source data of all repeats should be provided.

We therefore invite you to take these points into account when revising the manuscript. In addition, when preparing the revision please:

- ensure that it conforms to our format instructions and publication policies (see below and www.nature.com/nature/authors/).

- provide a point-by-point rebuttal to the full referee reports verbatim, as provided at the end of this letter.

Nature Cell Biology is committed to improving transparency in authorship. As part of our efforts in this direction, we are now requesting that all authors identified as ‘corresponding author’ on published papers create and link their Open Researcher and Contributor Identifier (ORCID) with their account on the Manuscript Tracking System (MTS), prior to acceptance. ORCID helps the scientific community achieve unambiguous attribution of all scholarly contributions. You can create and link your ORCID from the home page of the MTS by clicking on ‘Modify my Springer Nature account’. For more information please visit please visit www.springernature.com/orcid.

Please submit the revised manuscript files and the point-by-point rebuttal to the referee comments using this link:

Link Redacted

*This url links to your confidential home page and associated information about manuscripts you may have submitted or be reviewing for us. If you wish to forward this email to co-authors, please delete the link to your homepage.

We would like to receive the revision within four weeks. If submitted within this time period, reconsideration of the revised manuscript will not be affected by related studies published elsewhere, or accepted for publication in Nature Cell Biology in the meantime. We would be happy to consider a revision even after this timeframe, but in that case we will consider the published literature at the time of resubmission when assessing the file.

We hope that you will find our referees' comments, and editorial guidance helpful. Please do not hesitate to contact me if there is anything you would like to discuss.

Best wishes,

Stelios

Dr. Stylianos Lefkopoulos
He/him/his
Senior Editor, Nature Cell Biology
Springer Nature
Heidelberger Platz 3, 14197 Berlin, Germany

E-mail: stylianos.lefkopoulos@springernature.com
Bluesky: @slefkopoulos.bsky.social
LinkedIn: www.linkedin.com/in/stylianos-lefkopoulos-81b007a0

Reviewers' Comments:

Reviewer #1 (Remarks to the Author):

The revised manuscript has significantly strengthened the work. The new details re sample metadata, additional details of new computational pipelines are important as are the text changes. The removal of Figure 6/cancer data is also appropriate. I have no further concerns

Reviewer #2 (Remarks to the Author):

We have gone through authors' reply. They have done a great job to address most of the concerns, though some are beyond the capability to fully resolve, but reasonable in our opinion. Here are a few technical comments, mainly to improve the reproducibility of the work.

The description of the nichefinder framework would benefit from a more formal presentation of the underlying mathematical steps/derivations. While the authors discuss the general pipeline (k-NN graph construction, projection to a cell type interaction matrix, normalization, and NMF-based clustering), the underlying mathematical steps/derivations connecting these steps are not clearly described.
In particular, the authors should explicitly detail:
how the cell-cell adjacency matrix is constructed from spatial coordinates (including whether it is binary, weighted, heat kernel, etc),
how this adjacency matrix is transformed into a cell type-cell type interaction matrix using the cell type probability matrix. Please show the mathematical operations performed to obtain the interaction matrix.
how the final interaction matrix under the randomized background model is computed and used for normalization, and
how the non-negative matrix factorization leads to a soft clustering of cell types into niches. While the authors state that NMF provides a soft clustering of cell types, it is not sufficiently explained how the resulting weight matrix translates into niches.
Providing these derivations in the manuscript would greatly improve clarity and interpretability.

Again, the description of how NMF leads to niche identification requires further clarification. By definition, NMF decomposes a non-negative matrix X into two non-negative matrices W and H, such that $X \simeq WH$. However, this decomposition does not inherently perform clustering of the original data. It is therefore unclear which of the decomposed matrices (W or H) is interpreted as the cell type-by-niche matrix, and how this interpretation is justified. The authors should explicitly state how soft clustering is derived from this matrix.
More importantly, the manuscript should clarify how a low-rank factorization of a global interaction matrix translates into niches, rather than abstract latent components.
The terminology used to describe the nichefinder method and its output requires reconsideration. Referring to the algorithm as "nichefinder" and its results as "niches" is potentially misleading, as the inferred patterns appear to reflect global cell type co-localization or interaction profiles rather than spatially localized microenvironments. In the current form, the results seem more consistent with cell-type interaction states than spatial niches. The authors should revisit this interpretation and either provide stronger evidence that the inferred factors correspond to spatially localized niches (e.g., through direct spatial visualization) or adopt more precise terminology that better reflects the nature of the results.
The authors introduce Cell2home as a tool for cell-cell interaction analysis. However, its performance is not validated. Given that this is a method focused contribution included within a resource paper, it is important to demonstrate that the proposed Cell2home provides clear added value or, at minimum, performs comparably to established cell-cell interaction analysis frameworks e.g. by comparing overlaps or differences in identified interactions.
Lastly, please ensure that all previously published algorithms and methods used in this study are appropriately cited in the relevant sections.

Reviewer #2 (Remarks on code availability):

The codes are available and with detailed instruction and toy notebook. But I haven't thoroughly tested their algorithm/pipelines.

Reviewer #3 (Remarks to the Author):

I co-reviewed this manuscript with one of the reviewers who provided the listed reports. This is part of the Nature Cell Biology initiative to facilitate training in peer review and to provide appropriate recognition for Early Career Researchers who co-review manuscripts.

GUIDELINES FOR SUBMISSION OF NATURE CELL BIOLOGY ARTICLES

READABILITY OF MANUSCRIPTS – Nature Cell Biology is read by cell biologists from diverse backgrounds, many of whom are not native English speakers. Authors should aim to communicate their findings clearly, explaining technical jargon that might be unfamiliar to non-specialists, and avoiding non-standard abbreviations. Titles and abstracts should concisely communicate the main findings of the study, and the background, rationale, results and conclusions should be clearly explained in the manuscript in a manner accessible to a broad cell biology audience. Nature Cell Biology uses British spelling.

ARTICLE FORMAT

TITLE – should be no more than 100 characters including spaces, without punctuation and avoiding technical terms, abbreviations, and active verbs..

AUTHOR NAMES – should be given in full.

AUTHOR AFFILIATIONS – should be denoted with numerical superscripts (not symbols) preceding the names. Full addresses should be included, with US states in full and providing zip/post codes. The corresponding author is denoted by: "Correspondence should be addressed to [initials]."

ABSTRACT – should not exceed 150 words and should be unreferenced. This paragraph is the most visible part of the paper and should briefly outline the background and rationale for the work, and accurately summarize the main results and conclusions. Key genes, proteins and organisms should be specified to ensure discoverability of the paper in online searches.

TEXT – the main text consists of the Introduction, Results, and Discussion sections and must not exceed 3500 words including the abstract. The Introduction should expand on the background relating to the work. The Results should be divided in subsections with subheadings, and should provide a concise and accurate description of the experimental findings. The Discussion should expand on the findings and their implications. All relevant primary literature should be cited, in particular when discussing the background and specific findings.

ACKNOWLEDGEMENTS – should be kept brief. Professional titles and affiliations are unnecessary. Grant numbers can be listed.

AUTHOR CONTRIBUTIONS – must be included after the Acknowledgements, detailing the contributions of each author to the paper (e.g. experimental work, project planning, data analysis etc.). Each author should be listed by his/her initials.

FINANCIAL AND NON-FINANCIAL COMPETING INTERESTS – the authors must include one of three declarations: (1) that they have no financial and non-financial competing interests; (2) that they have financial and non-financial competing interests; or (3) that they decline to respond, after the Author Contributions section. This statement will be published with the article, and in cases where financial and non-financial competing interests are declared, these will be itemized in a web supplement to the article. For further details please see https://www.nature.com/licenceforms/nrg/competing-interests.pdf.

REFERENCES – are limited to a total of 70 in the main text and Methods combined,. They must be numbered sequentially as they appear in the main text, tables and figure legends and Methods and must follow the precise style of Nature Cell Biology references. References only cited in the Methods should be numbered consecutively following the last reference cited in the main text. References only associated with Supplementary Information (e.g. in supplementary legends) do not count toward the total reference limit and do not need to be cited in numerical continuity with references in the main text. Only published papers can be cited, and each publication cited should be included in the numbered reference list, which should include the manuscript titles. Footnotes are not permitted.

METHODS – Nature Cell Biology publishes methods online. The methods section should be provided as a separate Word document, which will be copyedited and appended to the manuscript PDF, and incorporated within the HTML format of the paper.

Methods should be written concisely, but should contain all elements necessary to allow interpretation and replication of the results. As a guideline, Methods sections typically do not exceed 3,000 words. The Methods should be divided into subsections listing reagents and techniques. When citing previous methods, accurate references should be provided and any alterations should be noted. Information must be provided about: antibody dilutions, company names, catalogue numbers and clone numbers for monoclonal antibodies; sequences of RNAi and cDNA probes/primers or company names and catalogue numbers if reagents are commercial; cell line names, sources and information on cell line identity and authentication. Animal studies and experiments involving human subjects must be reported in detail, identifying the committees approving the protocols. For studies involving human subjects/samples, a statement must be included confirming that informed consent was obtained. Statistical analyses and information on the reproducibility of experimental results should be provided in a section titled “Statistics and Reproducibility”.

All Nature Cell Biology manuscripts submitted on or after March 21 2016, must include a Data availability statement as a separate section after Methods but before references, under the heading "Data Availability”. For Springer Nature policies on data availability see http://www.nature.com/authors/policies/availability.html; for more information on this particular policy see http://www.nature.com/authors/policies/data/data-availability-statements-data-citations.pdf. The Data availability statement should include:

• Accession codes for primary datasets (generated during the study under consideration and designated as "primary accessions") and secondary datasets (published datasets reanalysed during the study under consideration, designated as "referenced accessions"). For primary accessions data should be made public to coincide with publication of the manuscript. A list of data types for which submission to community-endorsed public repositories is mandated (including sequence, structure, microarray, deep sequencing data) can be found here http://www.nature.com/authors/policies/availability.html#data.

• Unique identifiers (accession codes, DOIs or other unique persistent identifier) and hyperlinks for datasets deposited in an approved repository, but for which data deposition is not mandated (see here for details http://www.nature.com/sdata/data-policies/repositories).

• At a minimum, please include a statement confirming that all relevant data are available from the authors, and/or are included with the manuscript (e.g. as source data or supplementary information), listing which data are included (e.g. by figure panels and data types) and mentioning any restrictions on availability.

• If a dataset has a Digital Object Identifier (DOI) as its unique identifier, we strongly encourage including this in the Reference list and citing the dataset in the Methods.

We recommend that you upload the step-by-step protocols used in this manuscript to protocols.io. More details can found at https://www.protocols.io/help/publish-articles.

DISPLAY ITEMS – main display items are limited to 6-8 main figures and/or main tables. For Supplementary Information see below.

FIGURES – Colour figure publication costs $395 per colour figure. All panels of a multi-panel figure must be logically connected and arranged as they would appear in the final version. Unnecessary figures and figure panels should be avoided (e.g. data presented in small tables could be stated briefly in the text instead).

All imaging data should be accompanied by scale bars, which should be defined in the legend.
Cropped images of gels/blots are acceptable, but need to be accompanied by size markers, and to retain visible background signal within the linear range (i.e. should not be saturated). The boundaries of panels with low background have to be demarked with black lines. Splicing of panels should only be considered if unavoidable, and must be clearly marked on the figure, and noted in the legend with a statement on whether the samples were obtained and processed simultaneously. Quantitative comparisons between samples on different gels/blots are discouraged; if this is unavoidable, it has be performed for samples derived from the same experiment with gels/blots were

processed in parallel, which needs to be stated in the legend.

Figures should be provided at approximately the size that they are to be printed at (single column is 86 mm, double column is 170 mm) and should not exceed an A4 page (8.5 x 11"). Reduction to the scale that will be used on the page is not necessary, but multi-panel figures should be sized so that the whole figure can be reduced by the same amount at the smallest size at which essential details in each panel are visible. In the interest of our colour-blind readers we ask that you avoid using red and green for contrast in figures. Replacing red with magenta and green with turquoise are two possible colour-safe alternatives. Lines with widths of less than 1 point should be avoided. Sans serif typefaces, such as Helvetica (preferred) or Arial should be used. All text that forms part of a figure should be rewritable and removable.

We accept files from the following graphics packages in either PC or Macintosh format:

- For line art, graphs, charts and schematics we prefer Adobe Illustrator (.AI), Encapsulated PostScript (.EPS) or Portable Document Format (.PDF). Files should be saved or exported as such directly from the application in which they were made, to allow us to restyle them according to our journal house style.

- We accept PowerPoint (.PPT) files if they are fully editable. However, please refrain from adding PowerPoint graphical effects to objects, as this results in them outputting poor quality raster art. Text used for PowerPoint figures should be Helvetica (preferred) or Arial.

- We do not recommend using Adobe Photoshop for designing figures, but we can accept Photoshop generated (.PSD or .TIFF) files only if each element included in the figure (text, labels, pictures, graphs, arrows and scale bars) are on separate layers. All text should be editable in 'type layers' and line-art such as graphs and other simple schematics should be preserved and embedded within 'vector smart objects' - not flattened raster/bitmap graphics.

- Some programs can generate Postscript by 'printing to file' (found in the Print dialogue). If using an application not listed above, save the file in PostScript format or email our Art Editor, Allen Beattie for advice (a.beattie@nature.com).

Regardless of format, all figures must be vector graphic compatible files, not supplied in a flattened raster/bitmap graphics format, but should be fully editable, allowing us to highlight/copy/paste all text and move individual parts of the figures (i.e. arrows, lines, x and y axes, graphs, tick marks, scale bars etc). The only parts of the figure that should be in pixel raster/bitmap format are photographic images or 3D rendered graphics/complex technical illustrations.

All placed images (i.e. a photo incorporated into a figure) should be on a separate layer and independent from any superimposed scale bars or text. Individual photographic images must be a minimum of 300+ DPI (at actual size) or kept constant from the original picture acquisition and not decreased in resolution post image acquisition. All colour artwork should be RGB format.

FIGURE LEGENDS – must not exceed 350 words for each figure to allow fit on a single printed NCB page together with the figure. They must include a brief title for the whole figure, and short descriptions of each panel with definitions of the symbols used, but without detailing methodology.

TABLES – main tables should be provided as individual Word files, together with a brief title and legend. For supplementary tables see below.

SUPPLEMENTARY INFORMATION – Supplementary information is material directly relevant to the conclusion of a paper, but which cannot be included in the printed version in order to keep the manuscript concise and accessible to the general reader. Supplementary information is an integral part of a Nature Cell Biology publication and should be prepared and presented with as much care as the main display item, but it must not include non-essential data or text, which may be removed at the editor's discretion. All supplementary material is fully peer-reviewed and published online as part of the HTML version of the manuscript. Supplementary Figures and Supplementary Notes are appended at the end of the main PDF of the published manuscript.

Supplementary items should relate to a main text figure, wherever possible, and should be mentioned sequentially in the main manuscript, designated as Supplementary Figure, Table, Video, or Note, and numbered continuously (e.g. Supplementary Figure 1, Supplementary Figure 2, Supplementary Table 1, Supplementary Table 2 etc.).

Unprocessed scans of all key data generated through electrophoretic separation techniques need to be presented in a supplementary figure that should be labeled and numbered as the final supplementary figure, and should be mentioned in every relevant figure legend. This figure does not count towards the total number of figures and is the only figure that can be displayed over multiple pages, but should be provided as a single file, in PDF or TIFF format. Data in this figure can be displayed in a relatively informal style, but size markers and the figures panels corresponding to the presented data must be indicated.

The total number of Supplementary Figures (not including the "unprocessed scans" Supplementary Figure) should not exceed the number of main display items (figures and/or tables (see our Guide to Authors and March 2012 editorial http://www.nature.com/ncb/authors/submit/index.html#suppinfo; http://www.nature.com/ncb/journal/v14/n3/index.html#ed). No restrictions apply to Supplementary Tables or Videos, but we advise authors to be selective in including supplemental data.

Each Supplementary Figure should be provided as a single page and as an individual file in one of our accepted figure formats and should be presented according to our figure guidelines (see above). Supplementary Tables should be provided as individual Excel files. Supplementary Videos should be provided as .avi or .mov files up to 50 MB in size. Supplementary Figures, Tables and Videos much be accompanied by a separate Word document including titles and legends.

GUIDELINES FOR EXPERIMENTAL AND STATISTICAL REPORTING

REPORTING REQUIREMENTS – To improve the quality of methods and statistics reporting in our papers we have recently revised the reporting checklist we introduced in 2013. We are now asking all life sciences authors to complete two items: an Editorial Policy Checklist (found here https://www.nature.com/authors/policies/Policy.pdf) that verifies compliance with all required editorial policies and a Reporting Summary (found here https://www.nature.com/authors/policies/ReportingSummary.pdf) that collects information on experimental design and reagents. These documents are available to referees to aid the evaluation of the manuscript. Please note that these forms are dynamic 'smart pdfs' and must therefore be downloaded and completed in Adobe Reader. We will then flatten them for ease of use by the reviewers. If you would like to reference the guidance text as you complete the template, please access these flattened versions at http://www.nature.com/authors/policies/availability.html.

STATISTICS – Wherever statistics have been derived the legend needs to provide the n number (i.e. the sample size used to derive statistics) as a precise value (not a range), and define what this value represents. Error bars need to be defined in the legends (e.g. SD, SEM) together with a measure of centre (e.g. mean, median). Box plots need to be defined in terms of minima, maxima, centre, and percentiles. Ranges are more appropriate than standard errors for small data sets. Wherever statistical significance has been derived, precise p values need to be provided and the statistical test used needs to be stated in the legend. Statistics such as error bars must not be derived from n<3. For sample sizes of n<5 please plot the individual data points rather than providing bar graphs. Deriving statistics from technical replicate samples, rather than biological replicates is strongly discouraged. Wherever statistical significance has been derived, precise p values need to be provided and the statistical test stated in the legend.

Information on how many times each experiment was repeated independently with similar results needs to be provided in the legends and/or Methods for all experiments, and in particular wherever representative experiments are shown.

We strongly recommend the presentation of source data for graphical and statistical analyses as a separate Supplementary Table, and request that source data for all independent repeats are provided when representative experiments of multiple independent repeats, or averages of two independent experiments are presented. This supplementary table should be in Excel format, with data for different figures provided as different sheets within a single Excel file. It should be labelled and numbered as one of the supplementary tables, titled "Statistics Source Data", and mentioned in all relevant figure legends.

--------- Please don't hesitate to contact NCB@nature.com should you have queries about any of the above requirements ---------

Version 2:

Decision Letter:

10th May 2026

Dear Sarah,

Thank you for submitting your revised manuscript "Spatially resolved multiomics of human meninges development reveal lineage and disease dynamics" (NCB-RS59949B). It has now been seen by the original referees #2 and #3 and their comments are below. The reviewers find that the paper has improved in revision, and therefore we'll be happy in principle to publish it in Nature Cell Biology, pending minor revisions to comply with our editorial and formatting guidelines.

If the current version of your manuscript is in a PDF format, please email us a copy of the file in an editable format (Microsoft Word or LaTex)-- we cannot proceed with PDFs at this stage.

We are now performing detailed checks on your paper and will send you a checklist detailing our editorial and formatting requirements in about 1 to 2 weeks. Please do not upload the final materials and make any revisions until you receive this additional information from us.

Thank you again for your interest in Nature Cell Biology. Please do not hesitate to contact me if you have any questions.

Best wishes,
Stelios

Dr. Stylianos Lefkopoulos
He/him/his
Senior Editor, Nature Cell Biology
Springer Nature
Heidelberger Platz 3, 14197 Berlin, Germany

E-mail: stylianos.lefkopoulos@springernature.com
Bluesky: @slefkopoulos.bsky.social
LinkedIn: www.linkedin.com/in/stylianos-lefkopoulos-81b007a0

Reviewer #2 (Remarks to the Author):

Authors have sufficiently addressed all comments.

Reviewer #3 (Remarks to the Author):

I co-reviewed this manuscript with one of the reviewers who provided the listed reports. This is part of the Nature Cell Biology initiative to facilitate training in peer review and to provide appropriate recognition for Early Career Researchers who co-review manuscripts.

Version 3:

Decision Letter:

Dear Sarah,

I am pleased to inform you that your manuscript, "Spatially resolved multiomics of human meninges development reveal lineage and disease dynamics", has now been accepted for publication in Nature Cell Biology. Congratulations!

However, I would like to point out the following: I noticed the code is on github and not Zenodo; I would be extremely grateful if you could deposit the code to Zenodo or Code Ocean so that there is an accession number that is associated with the exact code used in the paper, since GitHub code can be updated and if that happens will not show the code used for this paper. If you can do this, you can update the Code availability statement and accession numbers/links at proof stage.

Thank you for sending us the final manuscript files to be processed for print and online production, and for returning the manuscript checklists and other forms. Your manuscript will now be passed to our production team who will be in contact with you if there are any questions with the production quality of supplied figures and text.

Over the next few weeks, your paper will be copyedited to ensure that it conforms to Nature Cell Biology style. Once your paper is typeset, you will receive an email with a link to choose the appropriate publishing options for your paper and our Author Services team will be in touch regarding any additional information that may be required.

After the grant of rights is completed, you will receive a link to your electronic proof via email with a request to make any corrections within 48 hours. If, when you receive your proof, you cannot meet this deadline, please inform us at rjsproduction@springernature.com immediately.

You will not receive your proofs until the publishing agreement has been received through our system.

Due to the importance of these deadlines, we ask that you please let us know now whether you will be difficult to contact over the next month. If this is the case, we ask you provide us with the contact information (email, phone and fax) of someone who will be able to check the proofs on your behalf, and who will be available to address any last-minute problems.

If you have any questions about our publishing options, costs, Open Access requirements, or our legal forms, please contact ASJournals@springernature.com

Once your paper has been scheduled for online publication, the Nature press office will be in touch to confirm the details. An online order form for reprints of your paper is available at https://www.nature.com/reprints/author-reprints.html. All co-authors, authors' institutions and authors' funding agencies can order reprints using the form appropriate to their geographical region.

Publication is conditional on the manuscript not being published elsewhere and on there being no announcement of this work to any media outlet until the online publication date in Nature Cell Biology.

You may wish to make your media relations office aware of your accepted publication, in case they consider it appropriate to organize some internal or external publicity. Once your paper has been scheduled you will receive an email confirming the publication details. This is normally 3-4 working days in advance of publication. If you need additional notice of the date and time of publication, please let the production team know when you receive the proof of your article to ensure there is sufficient time to coordinate. Further information on our embargo policies can be found here: https://www.nature.com/authors/policies/embargo.html

Please note that *Nature Cell Biology* is a Transformative Journal (TJ). Authors may publish their research with us through the traditional subscription access route or make their paper immediately open access through payment of an article-processing charge (APC). Authors will not be required to make a final decision about access to their article until it has been accepted. Find out more about Transformative Journals

Authors may need to take specific actions to achieve compliance with funder and institutional open access mandates. If your research is supported by a funder that requires immediate open access (e.g. according to Plan S principles or the NIH public access policy) then you should select the gold OA route, and we will direct you to the compliant route where possible. Because authors warrant under our subscription licensing terms that they haven't committed to licensing any version of their article under a licence inconsistent with the terms of our agreement – including the applicable embargo period – publication under the subscription model isn't suitable for authors whose funders require no embargo.

To assist our authors in disseminating their research to the broader community, our SharedIt initiative provides you with a unique shareable link that will allow anyone (with or without a subscription) to read the published article. Recipients of the link with a subscription will also be able to download and print the PDF.

If your paper includes color figures, please be aware that in order to help cover some of the additional cost of four-color reproduction,

Nature Portfolio charges our authors a fee for the printing of their color figures. Please contact our offices for exact pricing and details.

As soon as your article is published, you will receive an automated email with your shareable link.

If you have not already done so, we strongly recommend that you upload the step-by-step protocols used in this manuscript to protocols.io (https://protocols.io), an open online resource that allows researchers to share their detailed experimental know-how. All uploaded protocols are made freely available and are assigned DOIs for ease of citation. Protocols and Nature Portfolio journal papers in which they are used can be linked to one another, and this link is clearly and prominently visible in the online versions of both. Authors who performed the specific experiments can act as primary authors for the Protocol as they will be best placed to share the methodology details, but the Corresponding Author of the present research paper should be included as one of the authors. By uploading your Protocols onto protocols.io, you are enabling researchers to more readily reproduce or adapt the methodology you use, as well as increasing the visibility of your protocols and papers. You can also establish a dedicated workspace to collect your lab Protocols. Further information can be found at https://www.protocols.io/help/publish-articles.

You can use a single sign-on for all your accounts, view the status of all your manuscript submissions and reviews, access usage statistics for your published articles and download a record of your refereeing activity for the Nature Portfolio.

Nature Cell Biology encourages authors presenting evidence for cell, biological, molecular, and genetic interactions to consider communicating these findings using Biofactoid (https://biofactoid.org/). This tool helps users share a searchable representation of interactions (e.g. binding, gene expression, post-translational modification) between genes, gene products, or chemicals. Information added to Biofactoid, with author attribution, is shared on social media and public databases, such as Pathway Commons, where it can be discovered and analyzed in the context of a large and growing corpus of knowledge.

Please feel free to contact us if you have any questions.

With kind regards,
Stelios

Dr. Stylianos Lefkopoulos
He/him/his
Senior Editor, Nature Cell Biology
Springer Nature
Heidelberger Platz 3, 14197 Berlin, Germany

E-mail: stylianos.lefkopoulos@springernature.com
Bluesky: @slefkopoulos.bsky.social
LinkedIn: www.linkedin.com/in/stylianos-lefkopoulos-81b007a0

Click here if you would like to recommend Nature Cell Biology to your librarian
http://www.nature.com/subscriptions/recommend.html#forms

** Visit the Springer Nature Editorial and Publishing website at www.springernature.com/editorial-and-publishing-jobs for more information about our career opportunities. If you have any questions please click here.**

Open Access This Peer Review File is licensed under a Creative Commons Attribution 4.0 International License, which permits use,

sharing, adaptation, distribution and reproduction in any medium or format, as long as you give appropriate credit to the original author(s) and the source, provide a link to the Creative Commons license, and indicate if changes were made.
In cases where reviewers are anonymous, credit should be given to 'Anonymous Referee' and the source.
The images or other third party material in this Peer Review File are included in the article's Creative Commons license, unless indicated otherwise in a credit line to the material. If material is not included in the article's Creative Commons license and your intended use is not permitted by statutory regulation or exceeds the permitted use, you will need to obtain permission directly from the copyright holder.
To view a copy of this license, visit https://creativecommons.org/licenses/by/4.0/

We thank the reviewers for their time, expertise, and detailed review of our manuscript. We believe their suggestions are constructive, and have significantly improved our work. We present our response to their comments, and the changes we have completed below. The format of our reply is in order of the comments outlined by the reviewers.

Changes to our manuscript that include new text, and new figures, are shown in blue text throughout the response below.

Response to Reviewer 1

	COMMENT	RESPONSE
1.1	Sex information is absent from Figure 1b and supplementary data for each sample.<br><br>Given that each developmental age is represented by a single time point, sex represents an uncontrolled confound for gene expression differences.<br><br>The authors should provide sex information for all samples and, where possible (e.g., at 14 PCW where both Xenium and multiome data exist), test whether samples are from the same or different sexes and assess potential sex effects.	For the first trimester samples (BRC- prefix), sex information of the samples were not available as karyotyping is not performed for samples obtained from the bioresource through which the samples were obtained, but all samples were deemed normal following maternal biochemical tests (as the ethical approvals do not include aneuploidy collections - REC 96/085).<br><br>For HDBR, samples (12PCW onward), with pre-fix HDBR_HST, as below, karyotype information obtained from HDBR (N Solanky, co-author) was used to assign trisomy status, and sex status.<br><br>We have now included text within the methods section to clarify this as follows:<br>"Karyotype information was provided by HDBR for all second trimester samples, allowing assignment of ploidy and sex status. For assignment of samples in the first trimester, not obtained from the HDBR, gene-expression of chromosome Y genes was used as a surrogate (Supplementary Table. 2)."<br><br>Within Extended data Fig. 2a and b, we also include a new panel of Y chromosome gene expression across the samples, and identify clear segregation of samples, allowing assignment of sex information. This is conveyed through average expressed Y chromosome genes (gene-set available within Supplementary Table. 2) in Extended Data Fig. 2a.<br><br>These are now cited within the text as follows:<br>"Our data comprised tissues from 17 donors, ranging from 6 to 21 post-conception weeks (PCW) in developmental age, and include three aneuploidy samples with trisomy 21 (Fig. 1b, Extended Data Fig. 1a-f) and both male and female samples (Supplementary Table. 1, Extended Data Fig. 2a-b)"

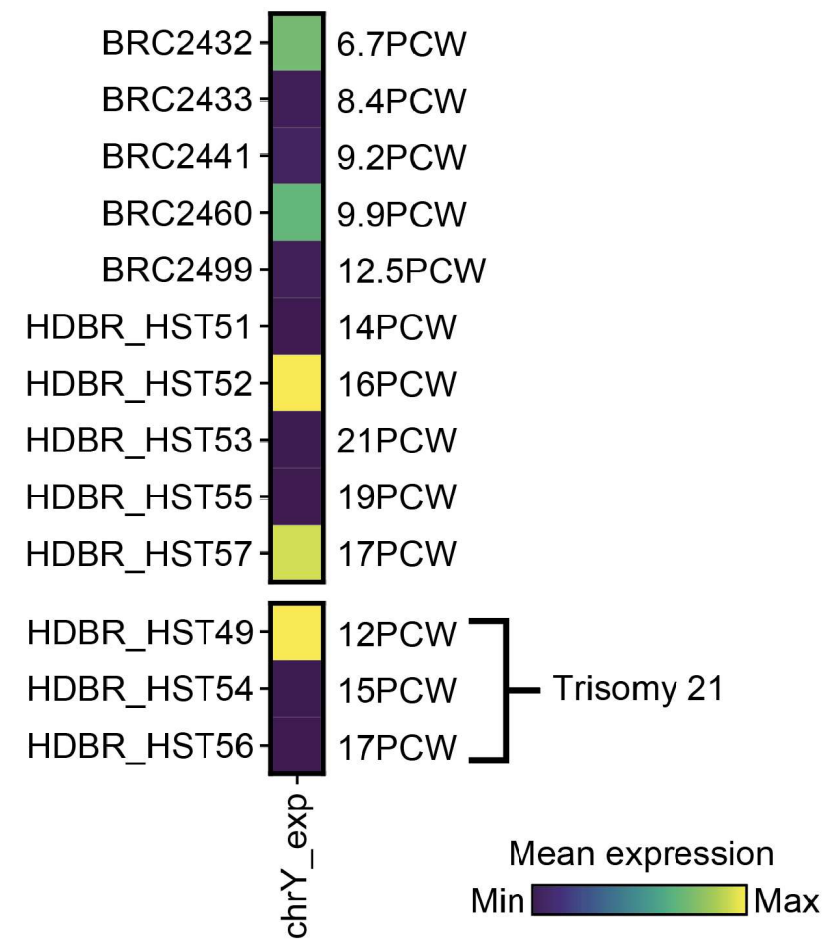

Extended Data Fig. 2**a.** Average expression of Chromosome Y genes within each donor sample.

A dotplot summarising the number of droplets per sample of the euploid atlas is then presented through Extended data fig. 2b.

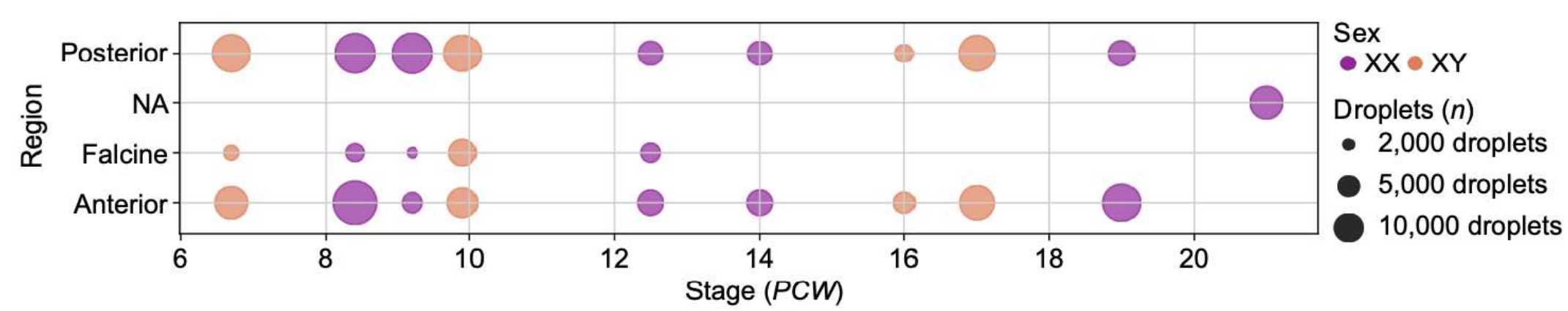

Extended Data Fig. 2b. Dotplot of sex and droplet number metadata for each donor.

Our spatial transcriptomics data generated do not contain Y chromosome gene-set probes, and we are therefore unable to infer sex information. However, we do not expect sex-specific effects within our dataset of non-gonadal early human development.

Within our study, we conducted a focused analysis on stage-differences in fibroblast cell states within our data (Fig. 2c). Here (Figure below), we repeat the analysis for female-only and male-only samples separately. These results were consistent with Fig. 2c, in recapitulating the differentially enriched cell states across early and later stages

		within the atlas. This suggests that broadly, large sex-associated transcriptional differences are not likely to be affecting our stage-related analyses within this work. We comment on this within the text as follows:<br><br>“A comparable pattern of enrichment was observed across both male and female samples (Extended Data Fig. 4a)”<br><br>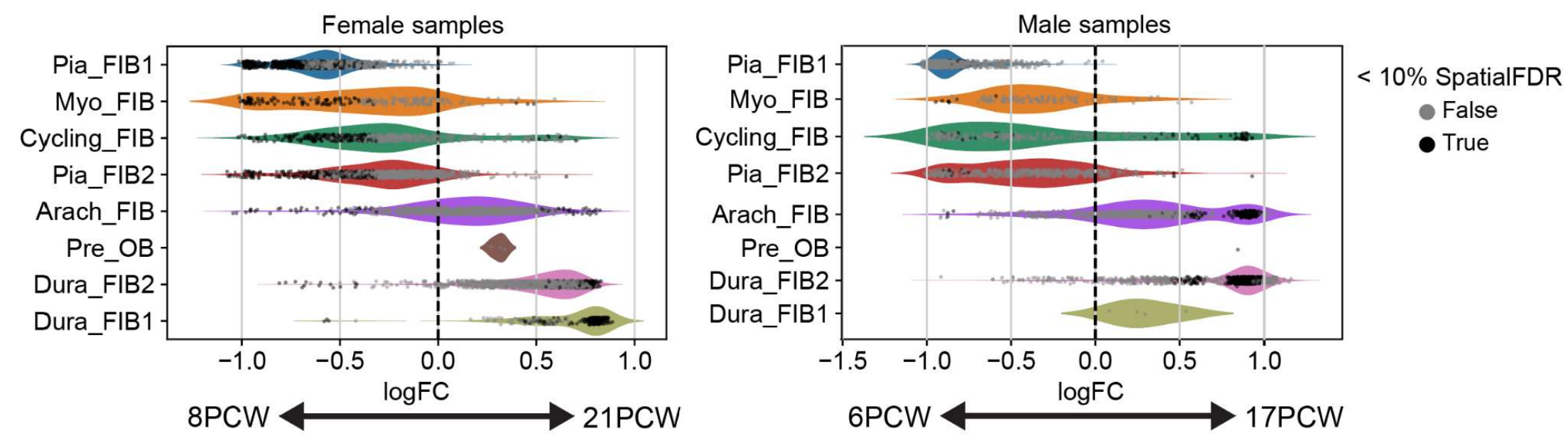<br><br><br>Extended Data Fig. 4a. Beeswarm plot of milo results comparing differential neighbourhood PCW enrichment of each fibroblast cluster within sex-subgrouped droplets.<br><br>We agree that given the number of samples studied per time-point, a future study investigating sex differences may be informative across fetal developmental data, however this may be beyond the scope of this current study, and may require significantly larger numbers of stage-matched samples to distinguish sex effects from stage-related effects on meningeal cell states within early-mid gestation. We comment on this in the revised manuscript as follows:<br><br>“Confounders to these possible trisomy 21 effects include maternal factors, genetic background and gestational stage, sex and therefore future work targeting a larger scale profiling of stage and sex matched aneuploidy states, and normal meningeal development will accelerate research into this important area of human developmental biology.”
1.2	Regional information is inadequately presented throughout. The UMAP in Figure 1c lacks annotation for sample age and region, and this information is not provided in supplementary data. It is	Our droplet dataset was “spatially resolved” in the context of mapping cell states onto spatial transcriptomic datasets. However, we did not focus on regionally-resolving meningeal fibroblast development. The main reason for this is because the anterior-enriched progenitors, namely neural crest cells, were not captured within our atlas.<br><br>The *bona fide* form of neural crest progenitors do not persist beyond 4PCW, and sample acquisition of early stage embryos is a challenging endeavour logistically. We have now performed some region-based analyses, and gene-set enrichment analysis of the anterior-derived fibroblasts, we hope this information, available within this dataset, stimulates interest and is useful for future work performing focused analyses of anterior-versus-posterior

<table>
<tr>
<td></td>
<td>unclear whether regions from the same age were pooled, whether all regions were collected from all ages, and how regional differences were analyzed. Spatial data throughout the manuscript similarly lacks regional annotation.

Given known developmental differences in regional origins of meninges, this represents a significant analytical gap that undermines the "spatially resolved" claim in the title.</td>
<td>derived cell states within the meninges.

We have now included summary dotplots, and supplementary tables with information regarding sample age, region and sex representation. We additionally generate UMAP plots in Extended data figure 2c, d and e.
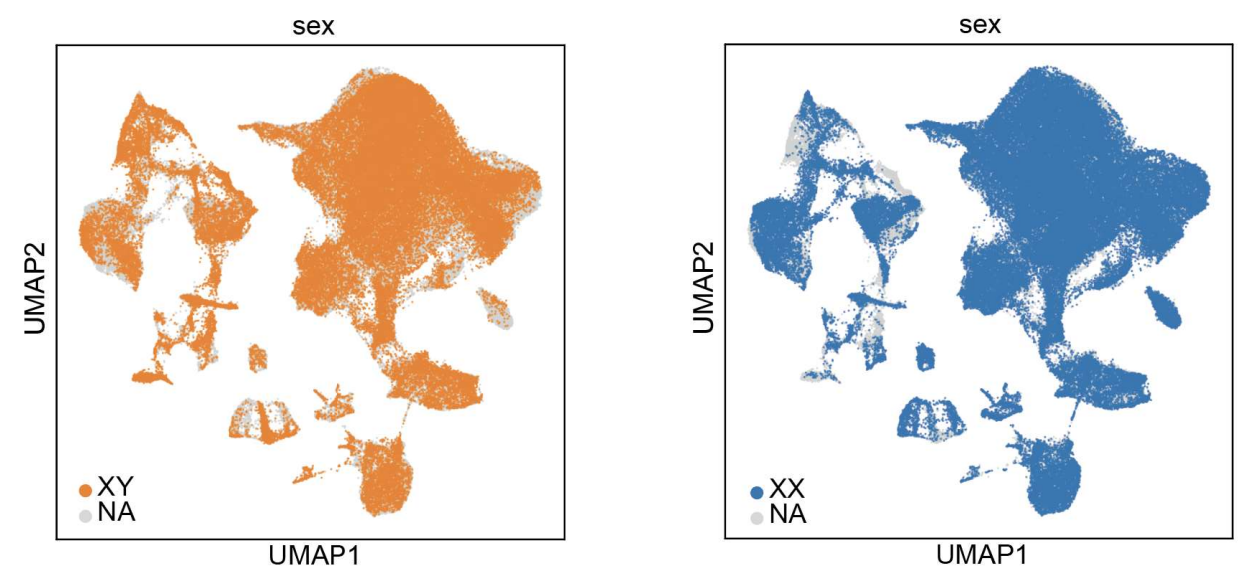

Extended Data Fig. 2c. UMAP plots of sample sex metadata
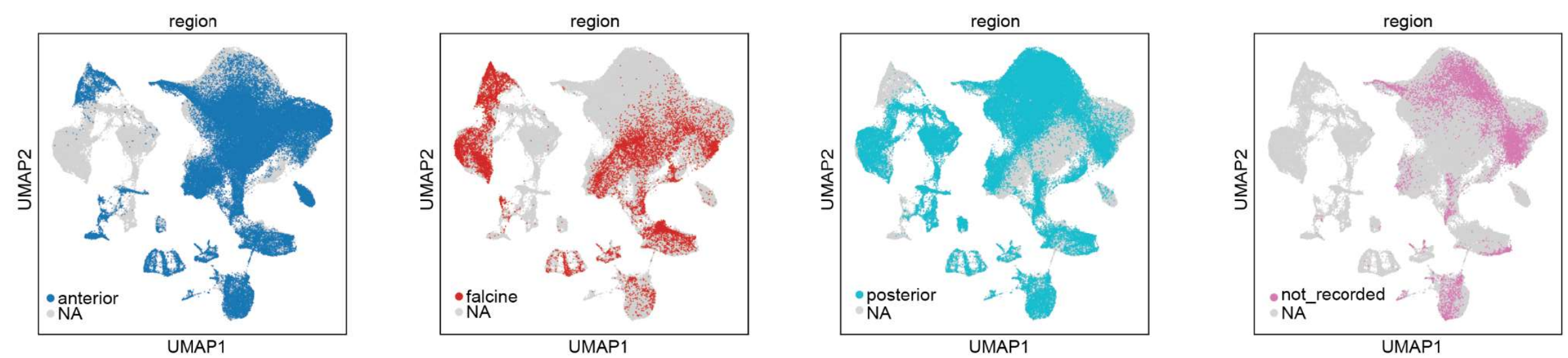

Extended Data Fig. 2d. UMAP plots of sample region metadata</td>
</tr>
</table>

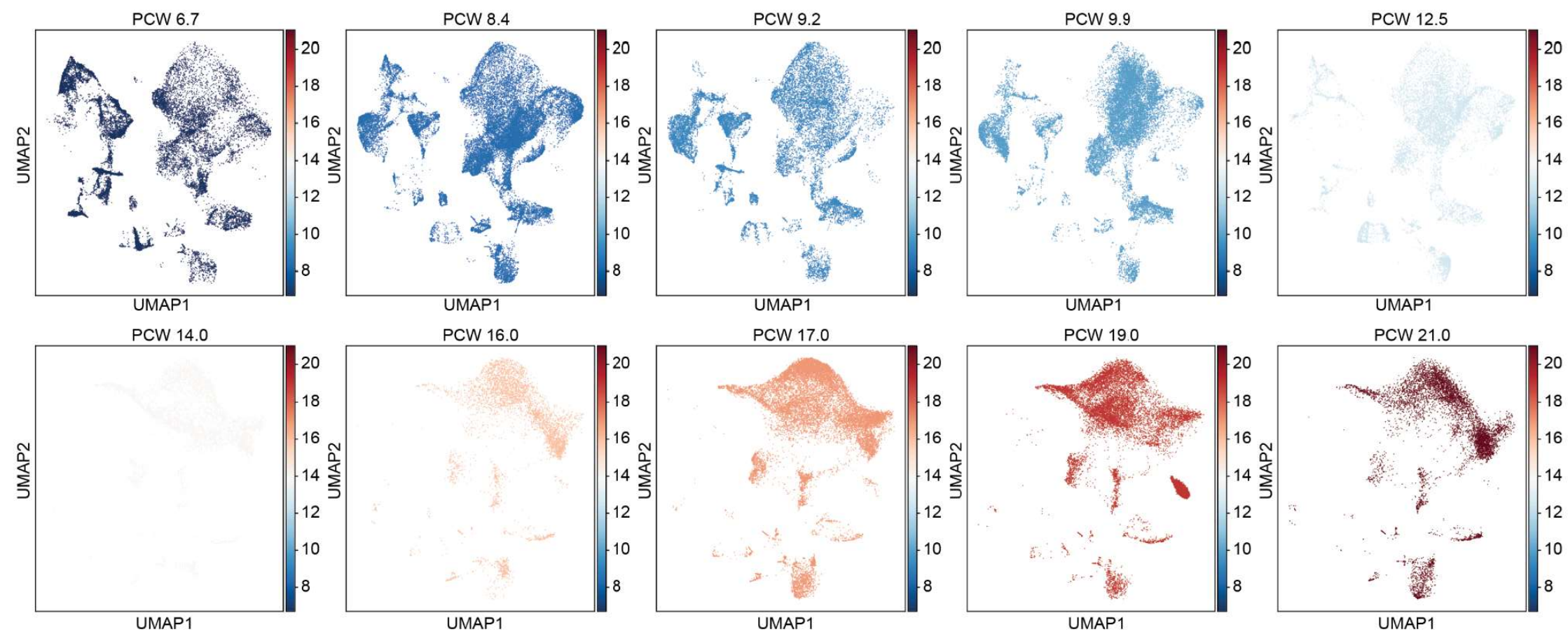

Extended Data Fig. 2e. UMAP plots of sample stage (/PCW)

For spatial data, we include access (see 2.22) to an interactive portal. Information pertaining to each xenium sample used within this study (matching for PCW stage) are now available within Supplementary Table 1 & 4.

We have now also analysed region differences for gene pathways within fibroblasts, and include these findings in Extended data fig. 4b as shown below:

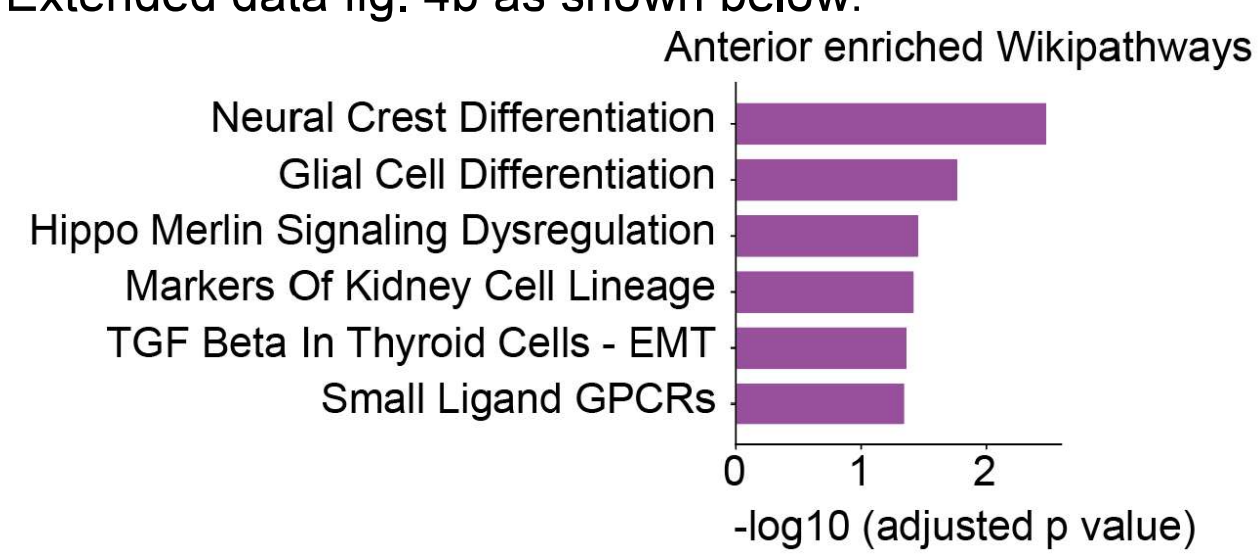

Extended Data Fig. 4b. Functional enrichment of Wikipathway database terms in fibroblasts (grouped) from the anterior region samples.

The output of this analysis, with per gene information, is available in tabulated form, within Supplementary Table 3. We reference these findings and region-specific genes within the main results section as follows:

<table>
<tr><td></td><td></td><td>“Although neural crest progenitors were not captured in our dataset, gene-set enrichment analysis of anterior region-sampled fibroblast populations together, highlighted neural crest differentiation as the top enriched pathway (Extended Data Fig. 4b, Supplementary table. 3). “

Within the discussion section, we speculate the need for sampling at earlier embryonic stages to evaluate neural crest differentiation related to regionally-resolved biology as follows:

“However, the earlier neural crest progenitors of the anterior cranial meninges were not captured, and future studies that profile human developmental stages before 5 PCW will facilitate their characterisation.“</td></tr>
<tr><td>1.3</td><td>Nuclei numbers per age/sample are not reported, making it impossible to assess whether any ages are over- or under-represented in the analyses which may influence data interpretation.</td><td>As above, this information is now summarised within Fig. 1d and the exact numbers, and regions, are shown within Supplementary table 1 and 4, for both euploid and trisomy 21 droplets, and spatial data as below.
<table>
<tr><th>donor_id</th><th>anterior</th><th>falcine</th><th>NA</th><th>posterior</th><th>ploidy</th><th>sex</th><th>pcw</th></tr>
<tr><td>2432</td><td>2470</td><td>685</td><td>0</td><td>3775</td><td>euploid</td><td>M</td><td>6.7</td></tr>
<tr><td>2433</td><td>11856</td><td>1296</td><td>0</td><td>5205</td><td>euploid</td><td>F</td><td>8.4</td></tr>
<tr><td>2441</td><td>2163</td><td>819</td><td>0</td><td>4902</td><td>euploid</td><td>F</td><td>9.2</td></tr>
<tr><td>2460</td><td>4650</td><td>1003</td><td>0</td><td>7141</td><td>euploid</td><td>M</td><td>9.9</td></tr>
<tr><td>2499</td><td>2686</td><td>1754</td><td>0</td><td>2635</td><td>euploid</td><td>F</td><td>12.5</td></tr>
<tr><td>HST51</td><td>3754</td><td>0</td><td>0</td><td>3380</td><td>euploid</td><td>F</td><td>14</td></tr>
<tr><td>HST52</td><td>2452</td><td>0</td><td>0</td><td>2105</td><td>euploid</td><td>M</td><td>16</td></tr>
<tr><td>HST53</td><td>0</td><td>0</td><td>5973</td><td>0</td><td>euploid</td><td>F</td><td>21</td></tr>
<tr><td>HST55</td><td>9014</td><td>0</td><td>0</td><td>3635</td><td>euploid</td><td>F</td><td>19</td></tr>
<tr><td>HST57</td><td>7125</td><td>0</td><td>0</td><td>7821</td><td>euploid</td><td>M</td><td>17</td></tr>
<tr><td>HST49</td><td>5498</td><td>0</td><td>0</td><td>0</td><td>trisomy 21</td><td>M</td><td>12</td></tr>
<tr><td>HST54</td><td>0</td><td>0</td><td>1700</td><td>0</td><td>trisomy 21</td><td>F</td><td>15</td></tr>
<tr><td>HST56</td><td>1205</td><td>0</td><td>0</td><td>374</td><td>trisomy 21</td><td>F</td><td>17</td></tr>
</table>
Supplementary table 1. Droplet data metadata and summary information
<table>
<tr><th>donor_id</th><th>region</th><th>stage(/PCW)</th></tr>
<tr><td>Hob23</td><td>Frontal</td><td>19PCW</td></tr>
<tr><td>C166</td><td>whole_sagittal</td><td>9PCW</td></tr>
<tr><td>C172</td><td>whole_sagittal</td><td>7PCW</td></tr>
</table>
Supplementary table 4. Spatial data metadata and summary information</td></tr>
</table>

<table>
<tr>
<td>1.4</td>
<td>The rationale for why the 12 PCW sample was unsorted is unclear. I can find no sorting information in the Methods section</td>
<td>We did not perform flow cytometry enrichment by cell markers, but did enrichment for nuclei, using 7-AAD, to reduce the amount of debris prior to droplet capture. This was performed for all samples from 12PCW onward, as debris within the dissociated cell suspension increased. There was minimal debris for earlier gestation samples.

We have now clarified this within the methods section of the manuscript as follows:

“For samples within the first trimester of gestation (< 12 PCW), the resulting single-cell suspension contained minimal debris upon visual inspection under the microscope. However, for second trimester samples (>12 PCW), significant amounts of debris was present within the single-cell suspension and nuclei enrichment was performed, using a ThermoFisher Bigfoot Spectral Sorter following 7-AAD staining (BioLegend 420403).“</td>
</tr>
<tr>
<td>1.5</td>
<td>Time/developmental age is inconsistently represented across analyses. While shown in Figure 2c and Extended Figure 3, it is absent from many critical figures (e.g., Figure 1c UMAP, Figure 4b UMAP, Figure 4i spatial data), making it difficult to evaluate developmental trajectories and assess whether conclusions are fully supported by the data.</td>
<td>As above, we have now included a panel demonstrating PCW metadata for the global euploid atlas UMAP within Extended data fig. 2e.

We have now also included a UMAP of PCW metadata for the myeloid lineage, within Fig. 4b. For myeloid development, we use label transfer, integration, and marker gene expression to infer the progenitor origins (PRE_MAC), and postulate (consistent with mouse literature), that within human, cranial myeloid cells also derive from a yolk-sac myeloid-like progenitor that continuously seed the brain, and are therefore present across samples in development stages, rather than exclusively at early developmental stages (which would indicate two parallel sources of progenitors).
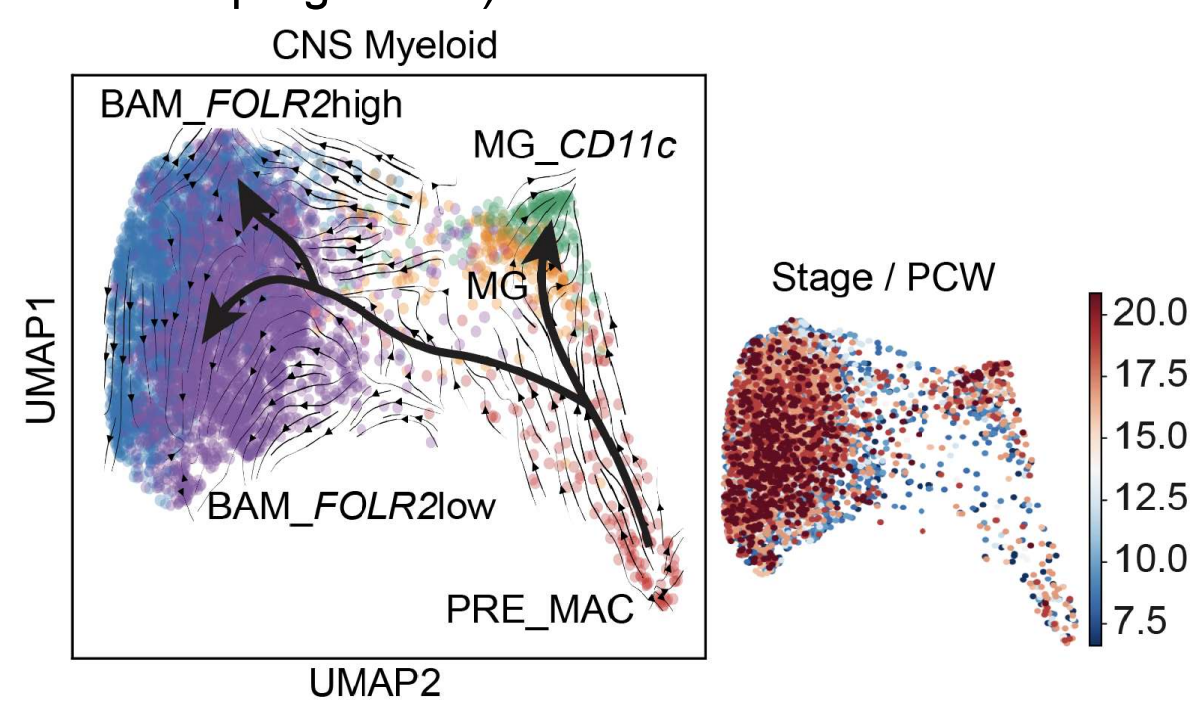

Fig. 4b: UMAP of CNS myeloid cells, with pseudotime arrows overlaid, and sample stage (/PCW) overlaid.</td>
</tr>
<tr>
<td>1.6</td>
<td>Several conclusions are well-supported by the data, particularly those regarding</td>
<td>We thank the reviewer for stating that our conclusions regarding B and myeloid lineage development are well-supported by our data.</td>
</tr>
</table>

	B-cell lineage development and Pre-MACC identity. However, some conclusions are either inadequately supported or overly speculative. Particularly problematics is the link hypothesized between MTORC1 pathway enrichment in the T21 immune compartment and downstream effects on Alzheimer's disease progression. This is inappropriate and should be removed entirely or substantially revised and supported by extensive functional data.<br><br>Also these hypotheses should be in the discussion section not the results.	We agree with your comments and have removed entirely any part of text suggesting a link between MTORC1 pathway enrichment and development of Alzheimer's in T21. Our updated text focuses on stating relevant findings previously reported within the literature, and we no longer mention Alzheimer's disease in any part of the manuscript.<br><br>Within the results section the updated text now reads as follows:<br>"We also observe MTORC1 signalling enrichment. In T21, hyperactivation of MTOR signalling has previously been linked to deficits in microglia-mediated processes such as autophagy and mitophagy."<br><br>We have moved text to the discussion section, and removed any discussion of Alzheimer's in T21, retaining the discussion of published findings on insulin resistance in T21; the updated text now reads as follows:<br>"Our finding of upregulated MTOR signalling is less clear. Increased MTOR signaling has been shown to be associated with insulin resistance in T21, possibly explaining the metabolic phenotype. However, robust functional experiments are needed to investigate."
1.7	Minor issue Figure 4 requires correction: panel l description is missing from the legend, and panel m is mislabeled as panel l	We have updated this figure legend accordingly, this is located underneath Figure 4 within the manuscript as follows, from panel g to m.<br><br>"**g, h.** Plot of membership nodes of spatial niches (numbered) for myeloid and vascular cell states in the first (9PCW) and second trimester (19PCW). Dotted line demarcates niches that include endothelial cell states (red), from those that do not, directly. **i.** Spatial plot of label transfer of Art_EC and BAM_FOLR2low cell states in ISS dataset. **j.** Protein staining images of perivascular macrophages in the meninges, likely representing BAM_FOLR2low. **k.** CellphoneDB results showing ligand-receptor interaction enrichment per cell type pair. **l.** Functional enrichment of angiogenesis modules in BAM cell states. **m.** MHC-II expression across BAM populations"

Response to Reviewer 2 & 3 (co-reviewers)

	COMMENT	RESPONSE
2.1	This resource paper included samples from 17 donors, from 6 to 21 PCB. However, clarification is needed on donor details. More detailed characteristics and potential confounding factors may affect the future analysis and interpretation of these valuable data.	We agree fully with the reviewer that these elements may affect future analysis and interpretation, which are beyond the scope of the current resource manuscript.<br><br>As per 1.1 and 1.2 , we have included dotplots showing donor droplet numbers, age, and sex in the euploid atlas, and have included supplementary table 1, which summarises droplets per donor, per region in the euploid as well as trisomy atlas. We also include more details on the spatial datasets (as above).<br><br>Additional details of these donors are not available, as maternal information is anonymised within both the BRC resource (first trimester samples), and mostly anonymised through the HDBR resource (second trimester samples).
2.2	Authors need to provide detailed information on the quality control steps for these dataset, as well as the following computational analysis, how to ensure the robustness and reproducibility and mitigate the batch effects. It is disappointing that certain inconsistencies in the results are explained simply as “sample error.” This raises concerns regarding sample handling, quality control, and analytical robustness.	We agree that robustness in sampling protocol is beneficial in resource papers. We wish to clarify that we did not experience sampling error, but instead discuss sampling bias, which may be present in all cell atlas studies that involve microdissection.<br><br>To address this we have undertaken the following:<br><br>1. Update main text (see below) to clarify our exploration of ACTA2 protein staining in our section and citation of available atlases of the adult meninges and first-trimester meninges (scRNAseq), which captured small numbers of *ACTA2*+ populations, and identified staining of ACTA2 within deeper regions of the meninges.<br><br>2. Updated Extended data fig. 3c to indicate superficial regions sampled, and deeper unsampled brain regions, including brain parenchyme regions.

	For a Resource paper intended to serve as a foundational dataset, the authors should more rigorously investigate discrepancies across donors, perform additional validation, or clearly document how such sample-level anomalies were identified and addressed.<br><br>Transparent resolution rather than attributing deviations to unspecified sample error is crucial for maintaining confidence in the dataset's reliability.<br><br>Moreover, it will be interesting to compare the newly generated data with open-source data to check the consistency.<br><br>Though no study has integrated data, but there are different studies with single platform data publicly shared.	c 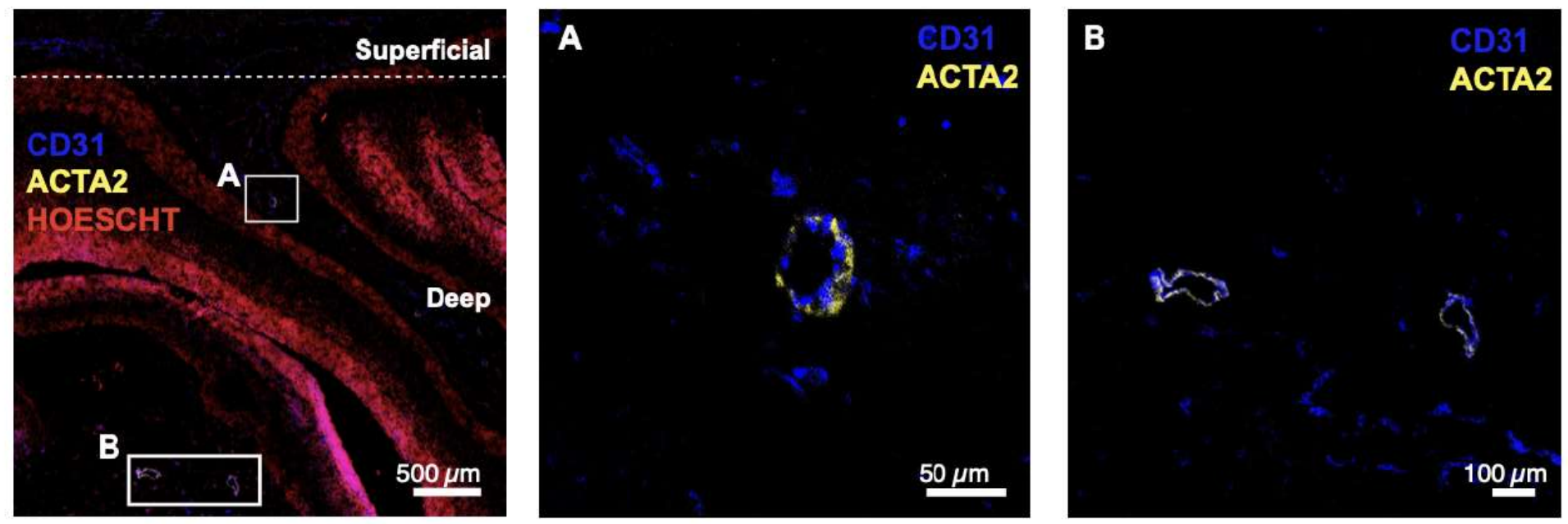 <br>3. Update main text (see below) to clarify that preosteoblasts contained within our samples may be due to inclusion of bone tissue in our sampling.<br><br>4. To validate our newly described fibroblast cell states externally, as advised by the reviewer, we have compared expression of layer-specific marker genes from our dataset against other available datasets of human fetal development, noting that no other available atlas provides comparable 2nd trimester samples for a direct comparison (please kindly see below within this response)<br><br>Text updates regarding ACTA2+ populations within the results section:<br>"We identify *ACTA2+* stromal cells in our data (Fig. 2a, b), and other publicly available single-cell datasets have identified *ACTA2+* mural populations in the developing and adult meninges, capturing them in small numbers (Vinsland et al, https://www.biorxiv.org/content/10.1101/2025.07.08.663122v2, and Degenhard et al, https://www.biorxiv.org/content/10.1101/2024.11.13.623440v1). We sought to investigate the spatial localisation of *ACTA2*-positive populations, within our samples, (Extended Data Fig. 3c) and applied protein staining of ACTA2 and CD31 to identify perivascular smooth muscle cells colocalization with endothelial cells within a 9 PCW sample that included large areas of brain regions. This revealed ACTA2+ populations around large-diameter blood vessels penetrating the brain parenchyma deeply, suggesting sampling of deeper brain regions may enrich for the cells of these vessels, whereas the the meninges overlying the brain surface, where we have sampled from, may contain less of these (Fig. 1e)."<br><br>Text updates regarding osteoblastic populations within the results section:<br>"We also detected a minute population of pre-osteoblasts (Pre_OB), strongly expressing *RUNX2*, a master regulator of osteogenesis, and *HHIP* which has been described to mark human cranial suture pre-osteoblasts, possibly related to inclusion of bone tissue adherent to the dura."

		We have now evaluated the *Vinsland et al* meninges scRNAseq atlas (5 - 13 PCW only) and have compared layer-specific marker expression in fibroblast clusters annotations. Both atlases report new descriptions of fibroblasts transcriptomes within the developing human meninges, and therefore was the focus of this comparison. Through this, we have identified consistent expression of marker genes across the linnarsson meninges layer populations. This is now included within Extended data figure 4c, and discussed within the main text as follows:<br>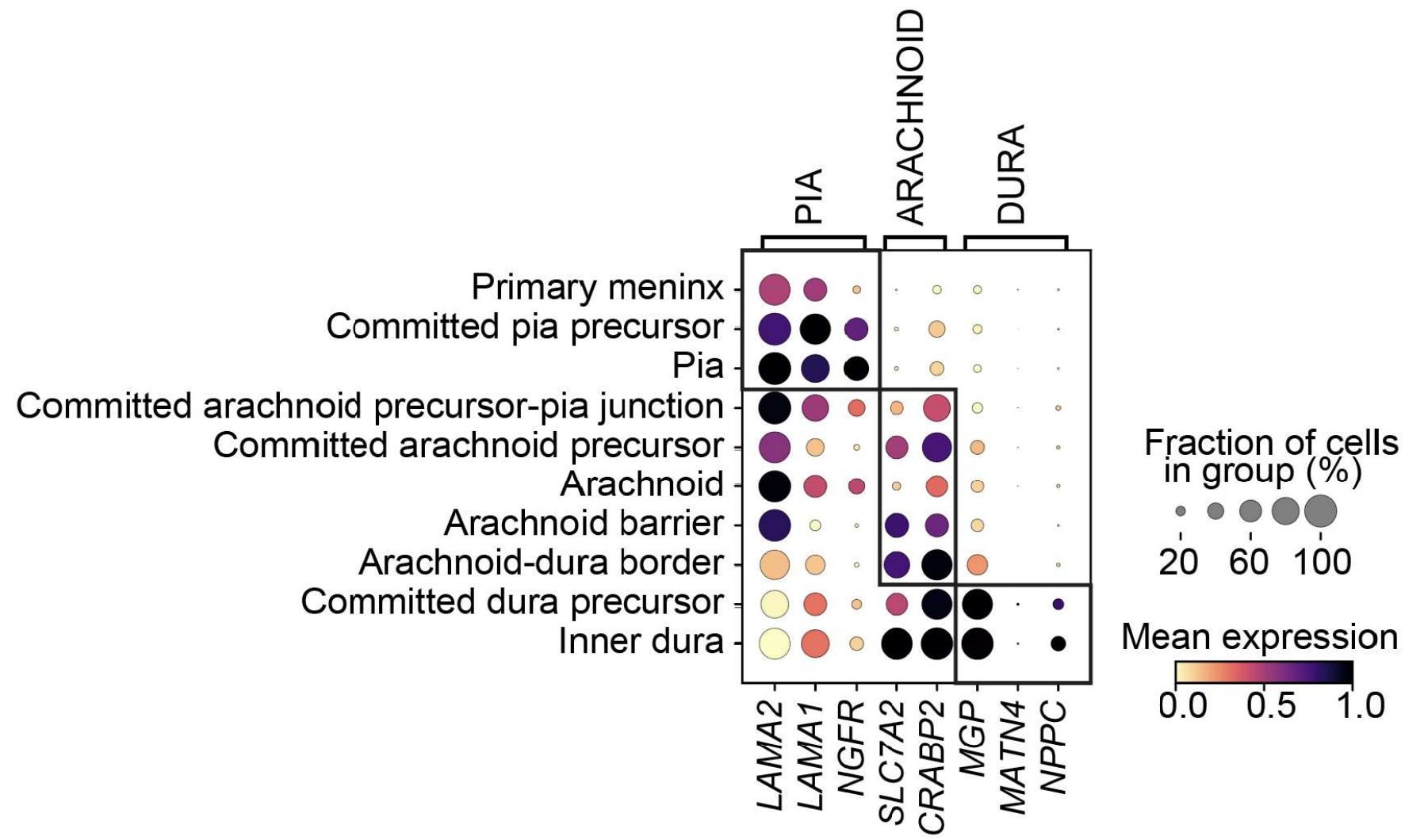<br><br>"Layer-specific marker genes from our dataset showed concordant expression patterns in an independent first-trimester human meningeal dataset (Vinsland et al. 2025), consistent with its layer-specific cell-type annotations (Extended Data Fig. 4c)."
2.3	Nichefinder, a new tool proposed to study fibroblast populations in meningeal tissues, is not benchmarked with other spatial mapping and cluster algorithms. The lack of such benchmarking	We conceptualised Nichefinder as a pipeline, or toolkit of individually established and interpretable steps. This is now explained in the methods section in more detail:<br><br>"To quantify the interactions between cell types in our atlas on each spatial slide, we sought after a direct and simple method. Most existing tools frame niche-detection as a problem of labelling each cell with a categorical niche-label. However, as our main goal was to contextualise the cell states annotated within the atlas using spatial information, we defined niche-detection in a more abstract way, as identifying groups of co-localising cell types. To this end, we set up a modular analysis pipeline of independent steps (Extended Data Fig. 6)".

<table>
<tr>
<td></td>
<td>raises concerns about its robustness and generalizability. The inclusion of Nichefinder in this resource paper without validation diminishes the rigor, can lead to potential biases in interpreting biology, especially in draft serve as foundational resource.</td>
<td>

This includes:

(1) cell type label-transfer to the spatial data,
(2) quantifying cell-type interactions and
(3) clustering cell-type interactions into niches.

This is now also illustrated as below:

a

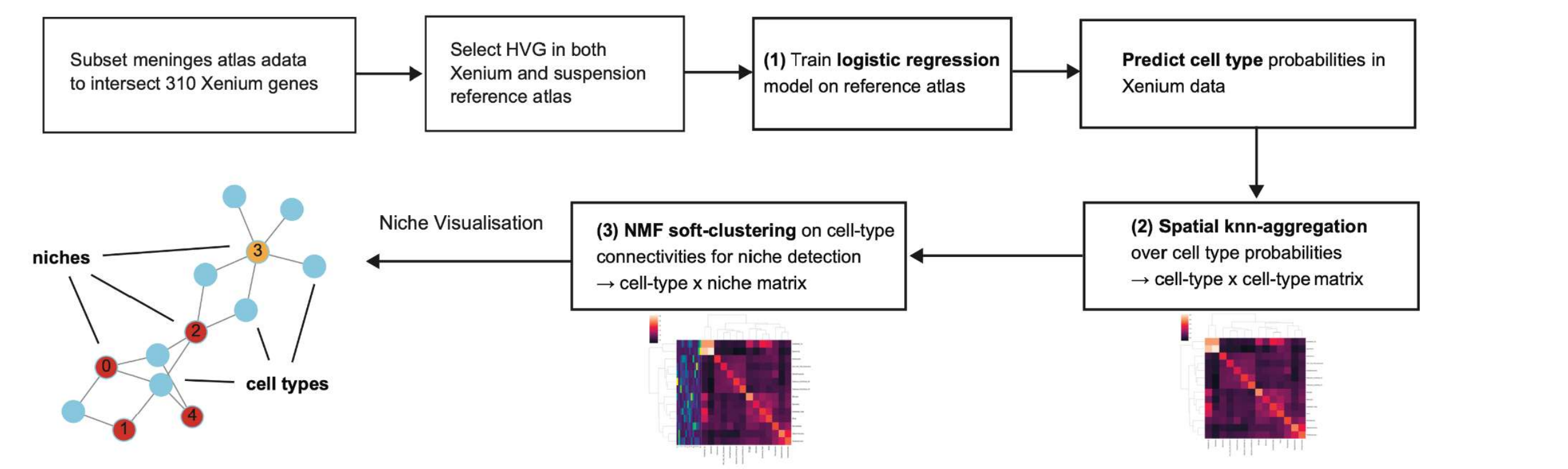

Extended Data Fig. 6 **a.** Nichefinder workflow showing highly variable gene (HVG) selection, followed by label predictions and negative matrix factorization (NMF) to identify spatial niches.

Therefore, we separate the use of gene expression profiles and spatial information in different steps: we first transfer cell type labels based on the former and quantify cell type interactions only based on the latter, making each step more interpretable. For all steps we choose methods that are established in single cell and spatial transcriptomics analyses (logistic regression, kNN-graph aggregation and NMF, respectively). Importantly, the last step only adds extra interpretation of the cell-type interaction matrix in the form of niche assignments. Focusing on cell-type-level interactions rather than labelling individual cells further circumvents dealing with single-cell-level noise and is easier to achieve without the use of complex modelling, thus simplifying the interpretation of results.

We now also include more detailed explanations for each step and a comparison with published methods in the methods section.
</td>
</tr>
</table>

	Cell-level niche assignment	Cell-cell interaction analysis
Simple complexity	e.g. Seurat neighbourhood k-means	nichefinder
High complexity	e.g. NicheCompass, SpaGCN, GraphST	e.g. NiCO, SpaceMarkers, Niche-DE

As nichefinder approaches niche-analysis in a unique way, direct comparison with published methods is difficult. NiCO(Agrawal et al. 2024) uses gene expression profiles for interaction analysis, but also focuses on cell-type interactions. We therefore, benchmarked nichefinder against NiCO on simulated data from the NiCO paper (Extended Data Fig. 6b-i):

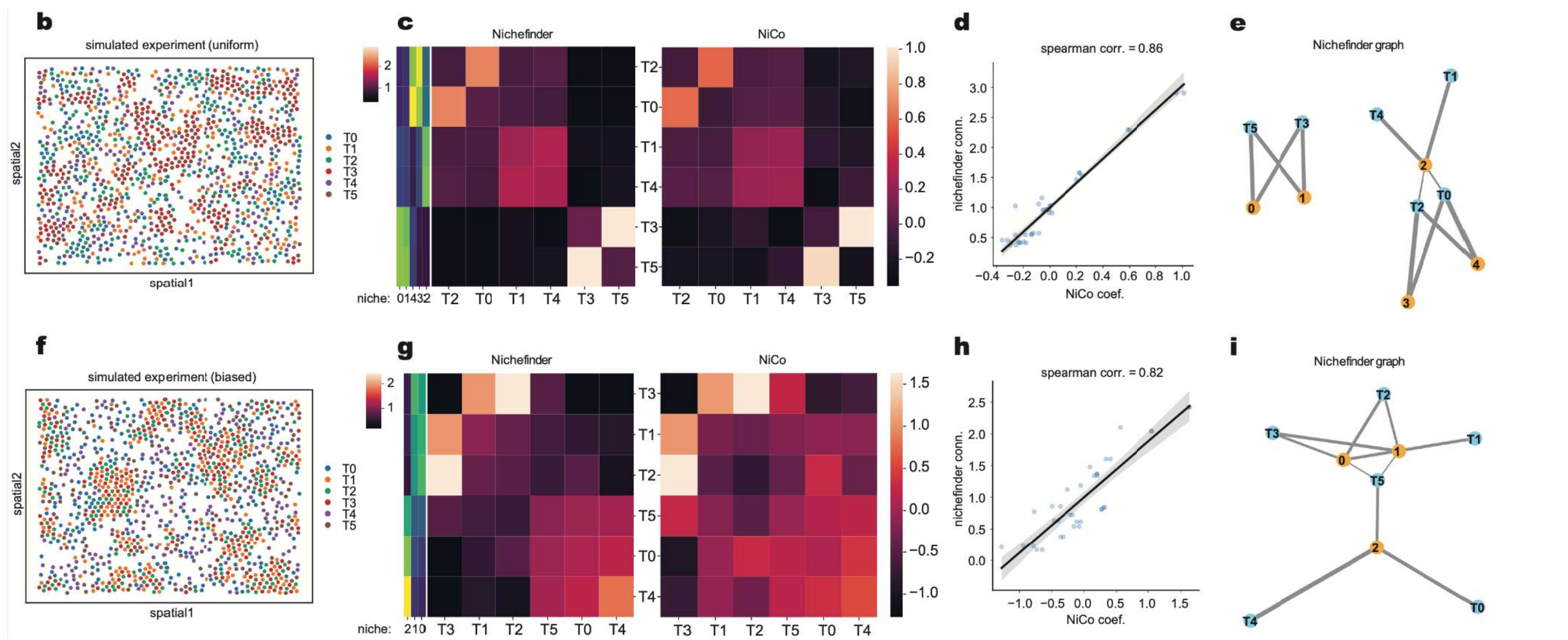

Extended Data Fig. 6 **b.** Simulated spatial transcriptomics data (uniform) from(Agrawal et al. 2024). **c.** Cell type interaction matrices predicted by nichefinder and NiCO for b. **d.** Scatter plot and correlation between nichefinder and NiCO cell type interaction matrices for c. **e.** Bipartite graph representation of d. **f.** Simulated spatial transcriptomics data (biased) from(Agrawal et al. 2024). **g.** Cell type interaction matrices predicted by nichefinder and NiCO for f. **h.** Scatter

		plot and correlation between nichefinder and NiCO cell type interaction matrices for g. **i.** Bipartite graph representation of i.<br><br>The cell type interactions detected by nichefinder and NiCO are nearly identical, with high correlations between them for two different simulated scenarios (0.86 and 0.82, Extended Data Fig. 6d,h), thereby validating the nichefinder approach. In addition, NMF-derived niches add another level of interpretation.<br><br>Although difficult to benchmark due to the different tasks that each method addresses, we aimed at comparing the nichefinder approach with cell-level niche assignments, using NicheCompass as an example (Extended Data Fig. 6j-p). Applied to mouse hippocampus data(Stickels et al. 2021), NicheCompass clearly annotates broad spatial domains (Extended Data Fig. 6k). To be able to compare the methods, we mapped nichefinder niche-memberships back to cells using the cell-type probability matrix (Extended Data Fig. 6i). Compared to NicheCompass, the nichefinder niches are more dispersed and resemble the original cell type label distribution more closely. When niche labels are mapped onto individual cell neighborhoods (a cell and its k nearest neighbors) using a weighted geometric mean to ensure all cell types in a niche are represented (using niche-membership weights), patterns of individual niches can be highlighted well (Extended Data Fig. 6m-o). This includes a relatively dispersed vascular niche 0, a localised niche 2 consisting of mostly ependymal cells and niche 10, a mix of ependymal cells and endothelial tip cells. When clustering Nichecompass and nichefinder niches together based on their cell-type memberships (Extended Data fig. 6p), a matching nichefinder niche can be found for at least half of the Nichecompass niches. Unsurprisingly, a block of NicheCompass niches that cannot be identified from the cell type labels (0, 1, 5, 11, 14), but map to structural domains(Birk et al. 2025), are not distinguished by nichefinder. In contrast, nichefinder focuses more niches on dispersed cell types such as astrocytes, microglia and endothelial cells, detecting more informative patterns for them (Extended Data Fig. 6m) compared to the

<table>
<tr>
<td></td>
<td></td>
<td>broad localised areas obtained from NicheCompas (Extended Data Fig. 6k).
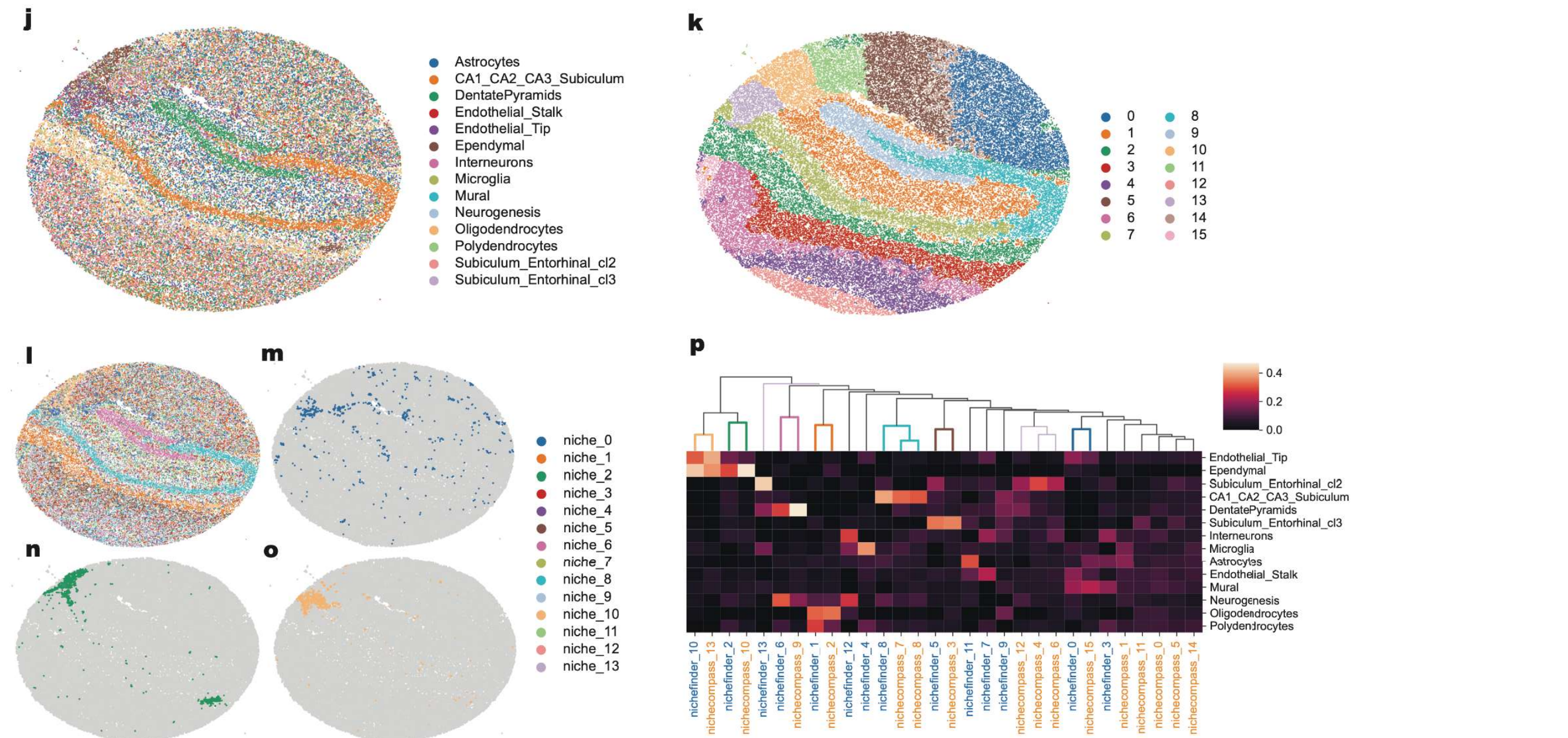

Extended Data Fig. 6 **j.** Cell type labels on mouse hippocampus data(Stickels et al. 2021). **k.** NicheCompass niche predictions for j. **l.** Nichefinder niches mapped back to individual cells using the cell type probability matrix. **m-o.** Nichefinder niches 0, 2 and 10 mapped to individual neighborhoods (each cell and its k=5 nearest neighbors) using a geometric mean weighted by niche-membership weights. **p.** Clustering of nichfinder and NicheCompass niches based on niche membership distributions, showing matches as well as distinct branches.

We have now included text within the methods section for all of the above points.</td>
</tr>
<tr>
<td>2.4</td>
<td>The study demonstrated the interesting insights into T21 impact on meningeal development, however, the sample size is limited.</td>
<td>We agree fully that these interesting insights could potentially be confounded by factors unaccounted for. In order to obtain information on these factors, a larger scale prospective study, with the appropriate ethical approvals may be required.

We have confirmed with our bioresource collaborators that most of this information is anonymised and cannot be obtained through the current ethical approvals. We have however now included sex information, and karyotype</td>
</tr>
</table>

	Moreover, the disease meachnisam is underexplored. Also, the potential confounding factors, such as maternal health, gestational age, genetic background of donors are not sufficiently addressed.	information, within Supplementary table 1.<br><br>We have also acknowledged these limitations within the discussion section as follows:<br><br>"Confounders to these putative trisomy 21 effects include maternal factors, and genetic background and gestational stage, and therefore future work targeting a larger scale profiling of aneuploidy states will accelerate research into this important area of human developmental biology."
2.5	Line 137: Please clarify how the label transfer on the integrated scRNA-seq atlas was performed.	We have now included a methods section describing this entitled "Label transfer using celltypist", including citation of celltypist algorithm, and the atlas from which the labels were derived, as follows:<br><br>"For characterisation of meningeal endothelial cell states, we performed automated annotation using a Celltypist model obtained from authors of an integrated atlas of human vascular cell types. Default parameters of the celltypist.annotate() function within celltypist 1.7.1 was used. Marker gene expression was then used to evaluate transferred labels."
2.6	Line 140: The manuscript references "Extended Data Fig. a,e," which is unclear. Please specify exactly which Extended Data Figures you are referring to.	This relates to extended data fig. 3a and e. The text has now been updated to reflect this.
2.7	Why was Pia_FIB1 assigned the earliest pseudotime values in Extended Data Fig. 3? Do you predefined Pia_FIB1 to be the start of the trajectory and if so any biological justification for that?	We appreciate this comment, and that pseudotime analysis is inferential and predictive in nature. For the root of the trajectory, this was defined through three main determinants. First, for the meningeal fibroblast populations, the Pia_FIB1 cell state was most differentially abundant for early PCW stages (Fig. 2c). Next, within the force-directed embedding it formed a visually discernible point within the embedding, suggesting it is likely to either be a terminal branch or root point. Then, based on the PCW metadata, overlaid on the forced-directed embedding, it most-correlated with early PCW, suggesting an earlier represented, possible progenitor cell state. Referring to Fig. 2d, we also observe the highest and earliest expression of *FAP*, a marker of activated fibroblasts, suggesting that this is possibly a progenitor population.<br><br>The text has been updated as follows:<br>"We modelled pseudotime, which correlated with PCW metadata, and predicted pia cell states as progenitors to arachnoid fibroblasts, and dural fibroblasts, with Pia_FIB1 assigned the earliest pseudotime values (Extended Data Fig. 4a), given its early PCW enrichment."

2.8	Lines 204-207: The authors cite "Supplementary Fig. 2," which does not exist. Do you mean Extended Data Fig. 6? Please correct this.	We apologise for this mislabelling, the reviewer is correct that this refers to extended data figure 6, the correct citation is reflected in the text now.
2.9	Please add a description of the stages depicted in the left panel to Fig. 3a legend.	We have now improved clarity on the PCW stages that are included in the trisomy analysis. This included 12-17PCW from three trisomic 21 samples (12, 15, 17 PCW) and the matched data from our euploid atlas within 12-17PCW (12,12.5,14,16 PCW). We have now included text both within the main text and the figure legend as follows:<br><br>Results section::<br>"We first age-matched aneuploidy samples (12, 15, 17 PCW) with euploid samples (12,12.5, 14, 16 PCW), and re-embedded the data together to allow direct comparisons (Fig. 3a)."<br><br>Fig. 3 legend:<br>"**a.** UMAP of the trisomy 21 samples from 12 to 17 PCW (12, 15, 17 PCW) and age-matched euploid samples (12, 12.5, 14, 16 PCW). Left panel shows overlaid metadata of stages, in PCW."
2.10	Lines 259-260: Please clarify what metadata PCA was performed on. Explain how PCA was applied to a metadata table containing both numerical and categorical variables, did you perform any preprocessing?	We have now included text within the methods section, entitled: "Pseudobulk enrichment analysis for trisomy samples", to explain the analysis undertaken, and citing the relevant tools, as follows:<br><br>"For the age-matched trisomy-euploid atlas, containing 12-17 PCW samples, the python implementation of decoupler was used to perform pseudobulking with default parameters. Briefly, pseudobulk profiles were generated for each cell type, and donor sample. Principal components were then computed, and each inferred PC was tested against covariates individually, including PCW stage, trisomy, and cell type, which captured a significant degree of variance (PC1 , PC2, PC3). Default parameters were utilised to filter for genes by min_total_counts=15. Differential expression analysis was conducted using the python implementation of DESeq2."
2.11	Lines 269-270: The correlation between chemokine genes and NF-κB downregulation is unclear. Please provide a description	We have sought to improve the clarity of the plot, and methods section. We have now stated the axis of the plots within the figure legends. I.e. that the y axis represents t-values, and the x axis are the weights. This is now updated within the legend of Fig.3 as follows:<br><br>'**i.** Target genes correlation with NFkB pathway enriched in stromal cells; x-axis values represent weights, and y axis are t-values…………………**l.** Target genes correlation with JAK-STAT pathway enriched in immune cells.x-axis values

<table>
<tr><td></td><td>at least in the Methods section of how this correlation analysis was performed.</td><td>represent weights, and y axis are t-values.’
Within the methods section under “Pseudobulk enrichment analysis for trisomy samples”, we have included text to explain the method used to calculate these as follows:

“For pathway enrichment analysis, we utilised the PROGENy pathway database, and calculated pathway scores using the univariate linear model (ulm) function within the decoupler package.”</td></tr>
<tr><td>2.12</td><td>Consider revising Fig. 4d to display the actual Euclidean distance values on the axis for transparent reporting.</td><td>We have now updated Fig. 4d to display the euclidean distances on each axis. Each axis has been updated, to begin from 0, increasing from left to right, bottom to top, as follows:
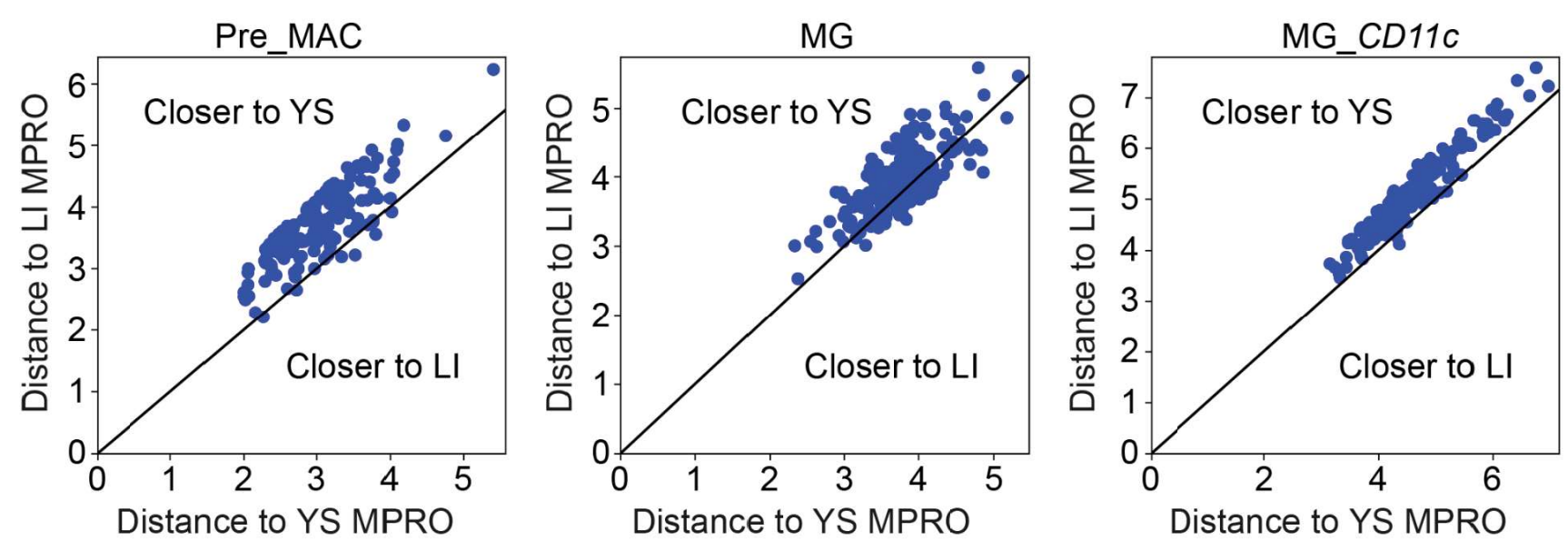

Fig. 4d. Plot of euclidean distance of scVI latent variable between MG_CD11c, MG and Pre_MAC SCVI to yolk-sac (YS) or liver (LI) myeloid progenitors when integrated with myeloid clusters from Suo et al.</td></tr>
<tr><td>2.13</td><td>The purpose of the dashed lines in Fig. 4g-h is unclear. If they indicate niche boundaries, please describe how the boundaries are defined on these networks. What criteria determine the end of the vascular niche, for example? Also, please correct the description of Fig 4g. in the figure legend.</td><td>Thank you for raising this issue. The dashed lines in Fig. 4g-h are manually included to separate the niches (numbered) that include endothelial cells, versus those that do not.

The figure legend for Fig. 4 has been updated to clarify this aspect as follows:
“**g, h.** Plot of membership nodes of spatial niches (numbered) for myeloid and vascular cell states in the first (9PCW) and second trimester (19PCW). Dotted line demarcates niches that include endothelial cell states (red), from those that do not, directly”

We have updated the legend of Fig. 5h accordingly, as that figure is related to methodology.</td></tr>
</table>

2.14	Lines 322-323: Here, how do you decide on the root of the trajectory (Pre_MACs)? Please provide the biological rationale for selecting this as the start of the trajectory.	Thank you for raising this point. The Cellrank package, CytoTRACE kernel, which has been benchmarked and tested on developmental haematopoietic lineages (Kang et al. 2025), was utilised to predict the myeloid differentiation trajectory (Fig. 4b). This did not require a root to be set, and therefore was an agnostic prediction of trajectory.<br><br>For myeloid populations within our data, we also use marker gene expression (Fig. 4a), label transfer (Fig. 4c), and integration (Fig. 4d), to infer our progenitor cluster (PRE_MAC), and include text to clarify this.<br><br>"Pre_MACs also closely resembled yolk-sac pre-macrophages and myeloid progenitors transcriptionally (Fig. 4c), gleaned by label transfer from atlases of the yolk sac (Goh et al), and pan-fetal tissues (Suo et al.)."<br><br>We have also updated the wording of the text to clarify that the pseudotrajectory is a prediction, as follows:<br><br>"Consistent with this, the Pre_MAC cluster expressed YS macrophage precursor markers *RAN* and *STMN1*, suggesting the possibility that these meningeal-resident YS-derived progenitors may be a source of meningeal myeloid cells. Furthermore by modelling pseudotime trajectory, we predict that Pre_MACs may differentiate toward BAMs and MG (Fig. 4a, b)."
2.15	Lines 356-358: Please list the top enriched biological processes and report these results atleast in the Supplementary Materials.	We have now included the gene-set utilised for scoring of this biological process within Supplementary table x.<br><br>We have also included a methods section to explain how this was undertaken, under "Gene set enrichment analysis for angiogenesis", as follows:<br><br>"For analysis of angiogenesis pathway-associated gene expression within the BAM populations, we obtained the Gene ontology (GO) biological process 2023 sprouting angiogenesis geneset (GO:0002040). For the cell states of interest, the scanpy function sc.tl.score_genes was utilised to compute the average expression of the gene set."<br><br>The main text has also been updated to reflect the above as follows:<br><br>"We performed gene-set expression analysis of angiogenesis pathways across BAMs populations (see methods) and highlighted enrichment in BAM_*FOLR2low* (Fig. 4l), which also expressed class II MHC strongly (Fig. 4m).."
2.16	Fig. 6h appears not to be cited in the manuscript. Please review and add the appropriate citation.	As advised by comments from reviewer 4, and the editor, regarding the overall contents of the manuscript, we have removed figure 6 and its associated analyses, results, and methods.

<table>
<tr>
<td>2.17</td>
<td>Lines 717-730 (cell migration analysis): Cell2home algorithm presented in this section is not yet published (Dufva et al., in preparation) and not in the reference list. Description of the algorithm provided in this section also does not clearly explain how the algorithms work. I will suggest the authors to consider using a peer-reviewed, validated algorithm.</td>
<td>We have now included the first description of the cell2home tool as part of this manuscript. We have included a description of the key steps of the tool in Results as well as a detailed description (Methods), along with a code repository (https://github.com/Teichlab/cell2home) and detailed function documentation with a vignette of the tool to allow users to perform analyses on their datasets (https://cell2home.readthedocs.io/en/latest/).
We have included new panels in Fig. 5, including a schematic summarising the tool workflow (Fig. 5f), and a downstream analysis of the strongest predicted receptor-ligand interactions driving the aggregated homing scores between B cells and vascular/stromal cells (Fig. 5h). Additionally, we have added a new Extended Data Figure 7 showing a full cell homing interaction matrix of all immune cell types and their attraction by vascular, stromal and other immune cell subsets.
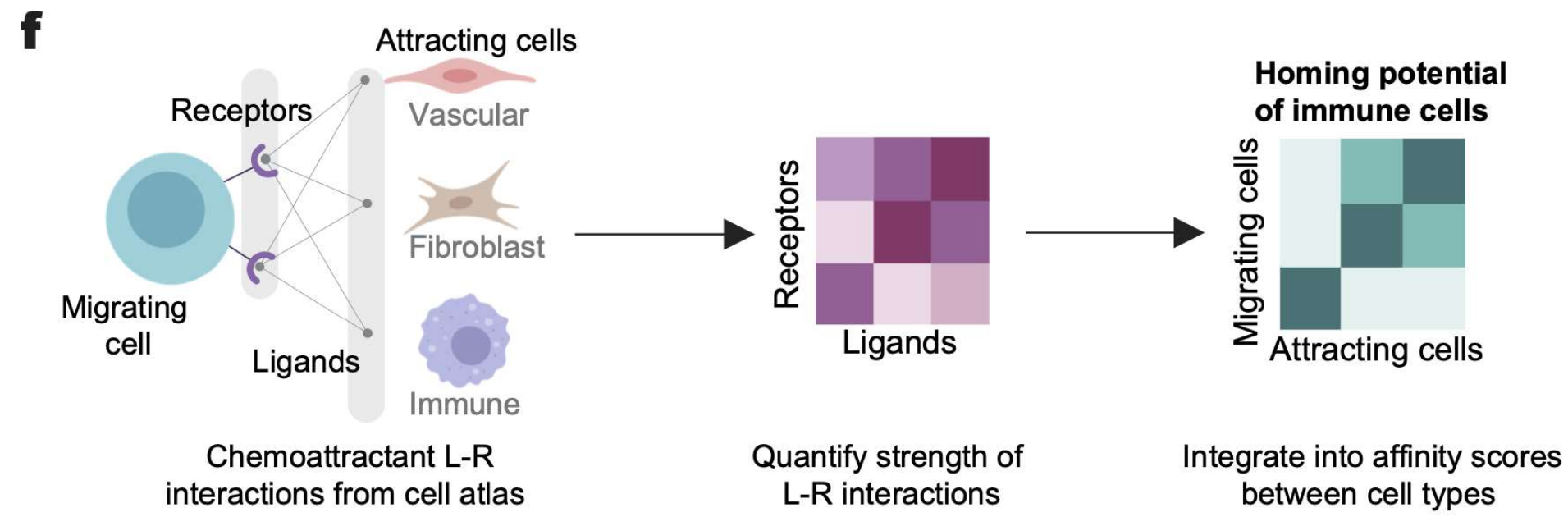

Fig. 5f: Schematic of the cell2home computational workflow.
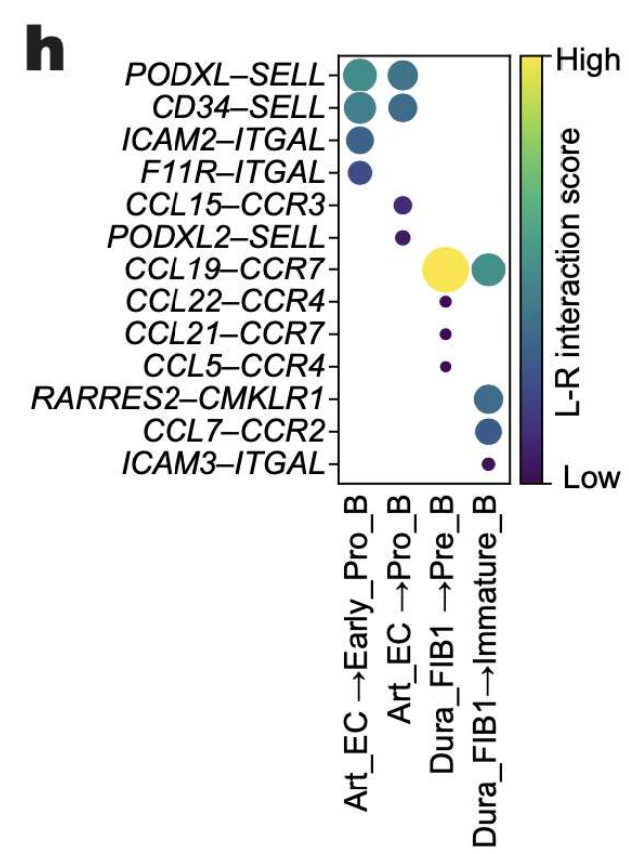

</td>
</tr>
</table>

Fig. 5h: Cell2home results showing ligand-receptor interaction enrichment per cell type pair, with the pair with highest contribution to the aggregate homing affinity scores shown.

Extended Data Figure 7a-b:

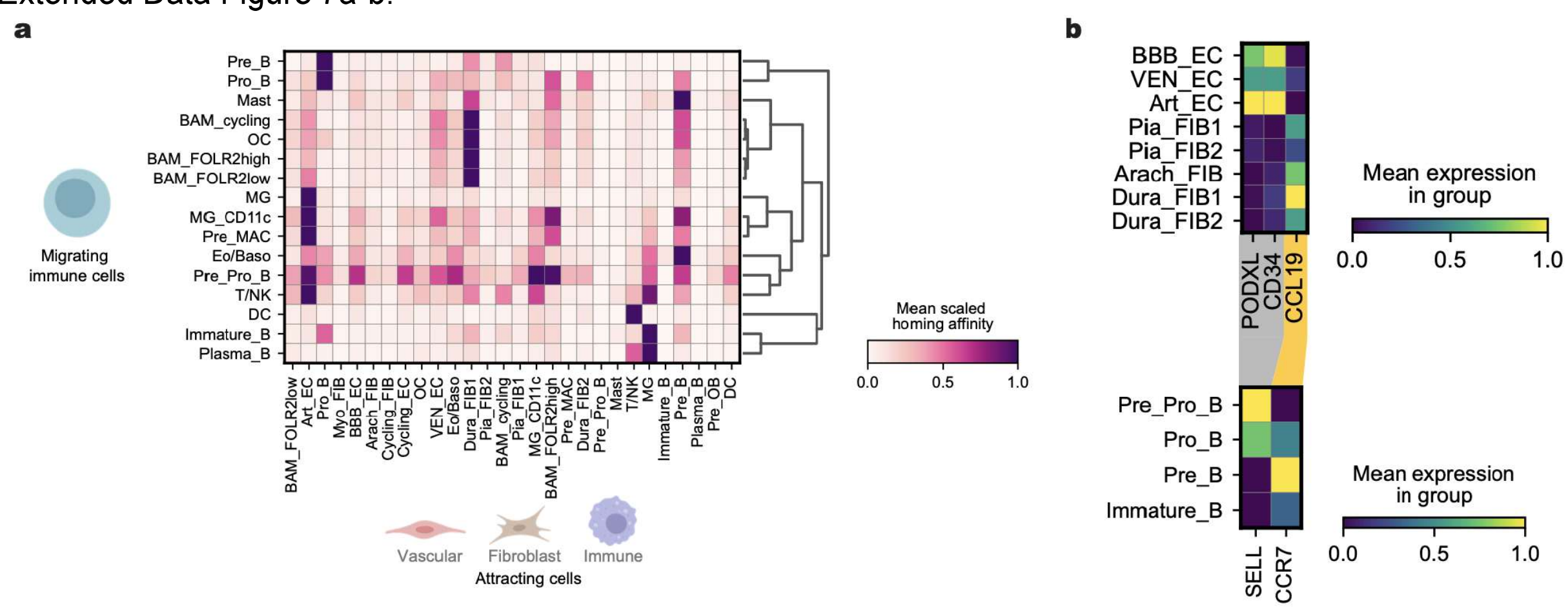

Extended Data Fig. 7. **a.** Homing affinity scores of immune cell subtypes towards vascular, stromal and other immune cells in the developing meninges. **b.** Expression of ligands for the top ligand-receptor interactions contributing to B cell homing affinity scores towards endothelial cells and fibroblasts and expression of corresponding receptors in B cell subtypes.

Text updates regarding cell2home within the results section:

"To comprehensively understand the trafficking of immune cells to tissue niches during meningeal development, we developed cell2home, a tool to computationally infer cell-cell homing interactions. Cell2home predicts which cell types (such as vascular, fibroblast other immune cells) immune cell subsets are attracted to based on a global chemokine receptor-ligand network. Cell2home first computes receptor-ligand interaction scores by multiplying the expression levels of each chemoattractant receptor with those of their cognate receptors, and then summarises these homing signals between a migrating immune cell and an attracting cell, repeating this across all migrating and attracting cell types to achieve a global immune cell homing landscape in the developing meninges (Fig. 5f, Extended Data Fig. 6a)."

"These homing interactions were predicted to be primarily driven by the CD34/PODXL-SELL ligand-receptor pairs between Early_Pro_B/Pro_B and ECs, and CCL19-CCR7 between Pre_B/Immature_B and Dural_FIB1, with a switch from SELL to CCR7 expression upon B cell maturation underlying the change in homing affinity (Extended Data Fig. 6b)."

		<u>Text updates regarding cell2home within the methods section:</u><br>"To predict homing of migrating immune cells towards attracting cells, we developed an analysis toolkit cell2home (implemented as a python package: https://github.com/Teichlab/cell2home). As a biological function-driven basis for the computational framework, we curated chemoattractant receptors from the literature into a homing interaction dictionary. We included only chemoattractant receptors that have been demonstrated to regulate migration of affinity towards cells expressing their cognate ligands in order to be able to summarise these signaling inputs from a variety of receptors into a single functional output, cell migration. These receptors include the CC, CXC, CX3C and XC chemokine receptor families as well as adhesion-mediating integrin receptors. Each ligand was linked to its cognate receptors using CellPhoneDB. This homing interaction dictionary is provided as part of the cell2home package.<br><br>First, a reference of chemoattractant ligand expression levels across attracting cell types is generated by pseudobulking the single-cell transcriptomes by cell type (and optionally additional cell metadata variables such as donor groups) by summing raw counts (with an option to pseudobulk using mean log-normalised counts for applicability across dataset types). The package comes with a streamlined, matrix operation based function for category-level pseudobulking capable of quick, memory efficient processing of larger datasets. This is followed by normalisation, log1p transformation, scaling, and extracting the resulting expression levels of the chemoattractant ligands for each pseudobulk profile. Ligand-receptor interaction scores are then computed by multiplying the expression of each receptor in the single cells with the cognate ligand expression in the reference cell types. The resulting interaction scores are averaged for each receptor across all ligands of the receptor in a given reference cell type to summarise all incoming migration cues received by one receptor from an attracting cell (receptor homing affinity). Subsequently the receptor level scores are aggregated using mean by attracting cell type to obtain scores measuring predicted affinity of the single cell to all attracting cell types. The resulting scores, including 1) ligand-receptor interaction scores, 2) receptor homing affinity scores and 3) final cell-level homing affinity scores are stored in a MuData object to facilitate downstream analyses and visualisation. Using the MuData object, cell2home allows extraction of the ligand-receptor pairs with the strongest predicted contribution to a homing interaction between two cell types and ranking of these interactions based on the ligand-receptor interaction scores."
2.18	Lines752-755: The authors mentioned "we subset genes for model training to highly variable genes shared between the two datasets. Next, we construct a cell-cell connectivity graph using the k-nearest neighbors (k-NN) algorithm to identify spatial	We have now added more details to the methods section and structured the text describing nichefinder more clearly to highlight the independent steps of the analysis workflow. This now better separates highly variable gene (HVG) selection, which is done as part of cell type label transfer (step 1), from k-NN construction, which is done as part of creating a cell-type by cell-type connectivity matrix (step 2). As above, we have further updated the flow diagram (Extended Data Fig. 6a) to reflect this separation into three steps, and we've also updated text within the methods section to clarify this as follows:<br><br><u>Specific added details include the number of HVGs and the selection process:</u><br>"Given the limited plexity of the ISS data, we subset genes for logistic-regression training to highly variable genes (HVGs) shared between the two datasets. Using scanpy "sc.pp.highly_variable_genes" with "min_disp=0.1" for HVG detection on both datasets this yields around 30-100 overlapping HVGs, depending on gene expression complexity of the analysed

	proximity between individual cells." The description of the Nichefinder pipeline lacks key details.<br><br>How many genes were selected as HVGs, and how were these determined?<br><br>How many nearest neighbors (k) were used in the k-NN graph?<br><br>Does the choice of nearest neighbor (k) influence the number or composition of detected niches?	section."<br><br>The number of nearest neighbors:<br>"We first construct a cell-cell connectivity graph using the k-nearest neighbors (k-NN) algorithm on spatial coordinates (with k=5) to characterise the proximity between individual cells."<br><br>Does the choice of k influence the number or composition of detected niches?<br>To evaluate the impact of different values for k on our data analysis, we have repeated nichefinder steps 2 and 3 multiple times for different values of k (3, 5, 15, 30, 60) using our meninges atlas and one of our Xenium slides (PCW 19). Using k=5 used in our analysis as a reference, for each k we calculated the correlation of the cell-type-by-cell-type connectivities obtained after step 2 (left panel) and the niche assignments obtained after step 3 (right panel) with the reference:<br>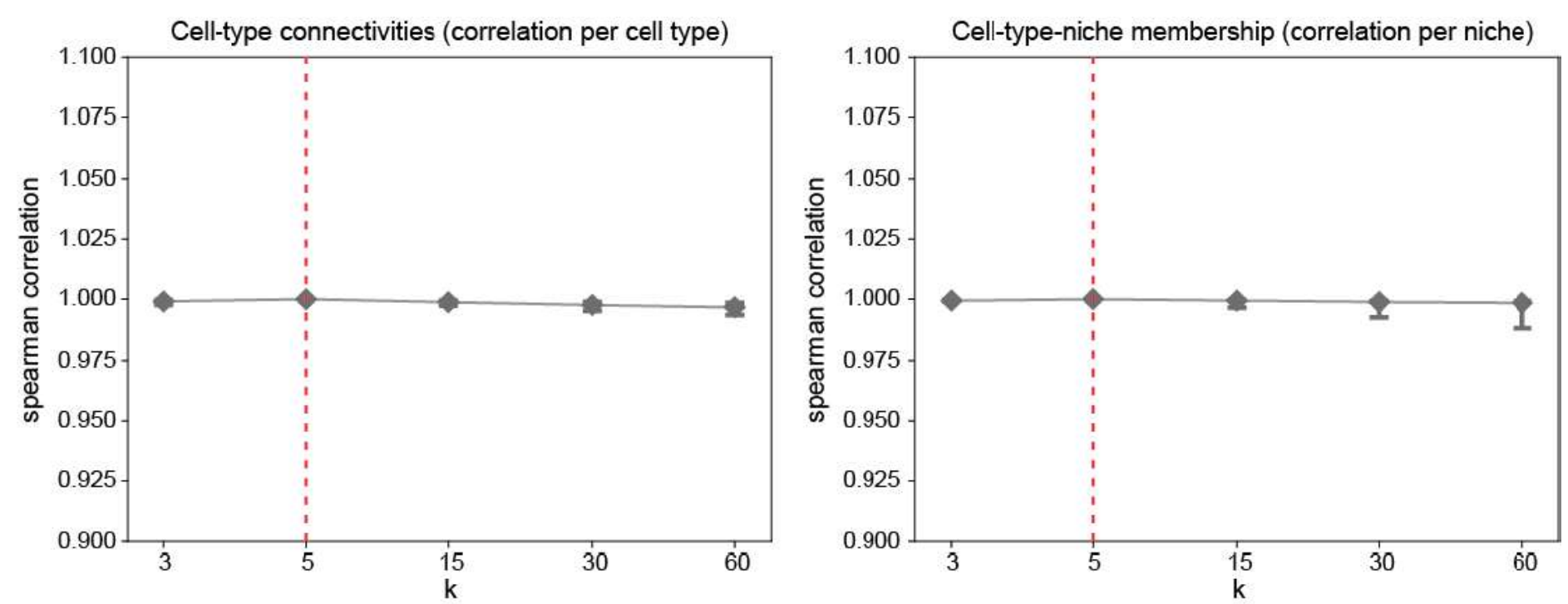<br><br>Results: For all tested values of k the aggregated cell-type connectivities are very similar, resulting in correlations near 1.0, which is also reflected in almost identical niche-assignments over the same automatically selected number of niches. We therefore conclude that the choice of k only has a minor effect on the niche assignments, which may reflect that downstream analyses are performed on matrices aggregated over many cells, improving robustness.
2.19	The manuscript states that "The soft-clustering property of NMF allows cell types to associate with multiple niches, capturing nuanced participation patterns across	In contrast to clustering methods such as k-means or leiden clustering, NMF returns a matrix of weights in which each cell type can be assigned to multiple clusters and vice versa (soft-clustering).<br><br>We have now highlighted this in the methods section:<br>"Finally, for niche clustering (3), the normalized cell-type by cell-type matrix is decomposed using non-negative matrix factorization (NMF), yielding a soft-clustering of cell types into niches (a cell-type by niche matrix of membership weights). The soft-clustering property of NMF guarantees that the same cell type can be part of multiple niches to capture more

	the tissue microenvironment." Please elaborate on how this is achieved in practice, i.e., associating celltype with multiple niches.<br><br>Additionally, please show niche structures directly on the tissue images prior to the network representation. This will allow readers to clearly see the niche structures and what celltypes are within each niche.	nuanced participation patterns (e.g. the same immune cell type in different spatial domains)."<br><br>Further, we now better clarify the purpose of nichefinder and how it differs from other methods:<br><br>"Most existing tools frame niche-detection as a problem of labelling each cell with a categorical niche-label. However, as our main goal was to contextualise the cell states annotated within the atlas using spatial information, we defined niche-detection in a more abstract way, as identifying groups of cell types that often co-localise."<br><br>As such, we do not map individual cells to niches, but compute an abstract representation of niches on the level of cell-types. This has the advantage of reduced noise after aggregating over cells (also see response to 2.18), but does not assign labels to individual cells or compute niche "structures". Therefore, we instead plot the network representation, which more directly represents the output of nichefinder.
2.20	Lines 774-778: How is the niche membership score computed? Please describe this and how the threshold was chosen.	We now describe that membership scores (or weights) are an output of NMF:<br><br>"Finally, for niche clustering (3), the normalized cell-type by cell-type matrix is decomposed using non-negative matrix factorization (NMF), yielding a soft-clustering of cell types into niches (a cell-type by niche matrix of membership weights)."<br><br>Further, we clarify that the use of a threshold is purely for visualisation purposes:<br><br>"The cell-type to niche assignments can also be visualised as a bipartite graph for an abstract representation of cell-type interactions bundled as niches. Similar to PAGA, a threshold on the weights in the cell-type by niche membership matrix can be applied to obtain a decluttered visualisation."
2.21	How are HVGs selected in Nichefinder? Does varying the number of HVGs produce stable niche structures, or do niche assignments change substantially?	We have now added additional details about HVG selection to the methods section as described above:<br><br>"Given the limited plexity of the ISS data, we subset genes for logistic-regression training to highly variable genes (HVGs) shared between the two datasets. Using scanpy "sc.pp.highly_variable_genes" with "min_disp=0.1" for HVG detection on both datasets this yields around 30-100 overlapping HVGs, depending on gene expression complexity of the analysed section."<br><br>Does varying the number of HVGs produce stable niche structures?

		We now also describe more clearly that HVG selection is only used for cell type prediction in the spatial transcriptomics data. Cell type prediction using a logistic-regression model with feature selection is widely used in the literature (Domínguez Conde et al. 2022) and it is expected that the selection of HVGs impacts cell type assignments. To test the effect of varying the number of HVGs on cell type prediction, we have run nichefinder multiple times for different values of the min_disp parameter (0.001, 0.01, 0.05, 0.1, 0.2, 0.5, 0.8) in scanpy "sc.pp.highly_variable_genes", which controls the number of selected genes, using our meninges atlas and one of our Xenium slides (PCW 19). Using min_disp=0.1 used in our analysis as a reference, for each min_disp value, we calculated the correlation of the cell-type assignments over all cells (left panel) with the reference:<br>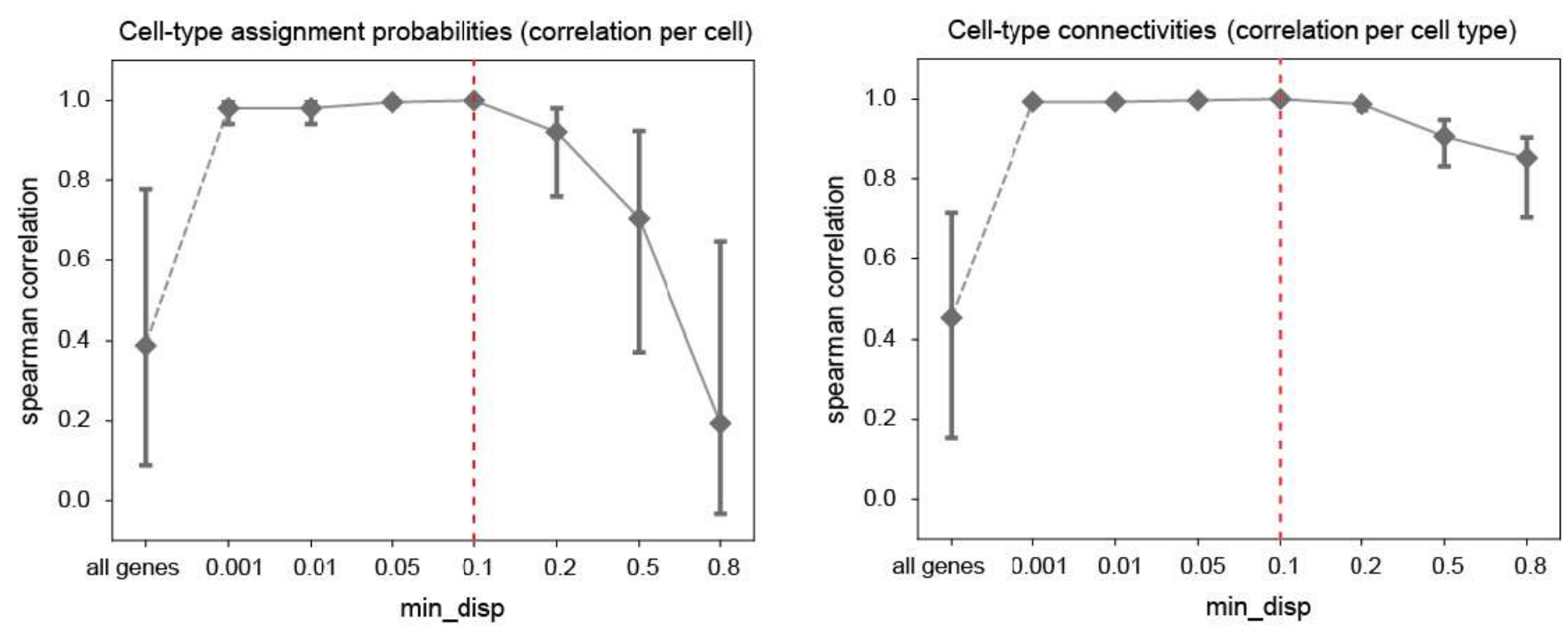<br><br>Results: As expected, larger values of min_disp, which decrease the number of HVGs below 30, result in differing cell type assignments, presumably reflecting insufficient information to distinguish cell types (left panel). Using all genes (no HVG selection) likewise leads to differing cell type assignments. To optimise the label transfer, we evaluated for different parameter choices whether cell types map to anatomical structures in a biologically expected way. Therefore, worse performance when assigning cell types using "all genes" may be due to increased noise from those additional genes. To this end, feature selection had been proposed for logistic-regression based label transfer (Xu et al. 2023).<br><br>Interestingly, when we examine the effect of altering the number of HVGs on downstream steps (step 2) in nichefinder (see right panel), we find that the reduction in correlation is less strong. Similar to altering the number of neighbors (see above), this may reflect the fact that we analyse matrices aggregated over many cells, which mitigates noise and inaccuracies on the single-cell-level. In addition, we make use of the full cell-type probability matrix of label-assignments to increase robustness.
2.22	Neither codes nor data are available to test	We acknowledge the importance of this for a resource manuscript and have had the data and code set on private mode, for public release upon publication. They can now be accessed as follows:

		For our droplet and spatial datasets, these can be accessed at the official human developmental cell atlas portal through the following link. It is currently password protected and will be made publicly available upon publication:<br>Link: https://developmental.cellatlas.io/meninges-development<br>Password: RpbYyqzY<br><br>The code and vignette for cell2home can be accessed in the following links:<br>https://github.com/Teichlab/cell2home<br>https://cell2home.readthedocs.io/en/latest/<br><br>The code and vignette for Nichefinder can be accessed in the following link:<br>https://github.com/Teichlab/nichefinder
2.23	The authors provided the link https://github.com/Teichlab/cell2homes as the location for the code, but the link is not working.	We have now made the cell2home tool source code available at https://github.com/Teichlab/cell2home and the tool is available for users to install as a package through pip. Detailed documentation of the package function and a Jupyter notebook vignette using an example dataset are available at https://cell2home.readthedocs.io/en/latest/.

Response to Reviewer 4

	COMMENT	RESPONSE
4.1	What I am prepared to say is that the data in figure 6 connecting development of the meninges and meningioma is not necessary in this paper, and if it were to be included, should be better thought through and analyzed.	We thank the reviewer for this important comment, and given the options and suggestions presented by the editorial team, we have moved to remove all aspects relating to the meningioma analysis from our manuscript. We thank the reviewer for their valuable insights below.
4.2	The single cell data on meningiomas that is used here is from two published datasets (fig 6a). But the WHO grade and genetic background (ch22 loss, ch1 loss, ch10 loss, ch14 loss etc) of these tumors is not noted in this paper. For example, which of them are NF2 mutant, vs NF2 wildtype and mutant for other genes such as Akt, Smo, KLF4 etc? this matters because the biology of these tumors is different, especially given the discussion of the similarities between meningeal development and NF2 function. Yet in 6b, all the tumors have the most similarity to duraf1b1	We have removed the meningioma analysis, as per the recommendations of the reviewers and the editorial team.<br><br>We agree there is a large degree of heterogeneity, within a small meningioma dataset. Ideally a large, expanded meningioma single-cell atlas would be the best input for a comparative analysis. This is likely to require a large, extensive and challenging piece of future work.<br><br>Within our (now removed) analysis, we postulated that the consistent cell-type matching to dura_fib1 is likely due to common, shared transcriptional programs, across meningioma. However, we acknowledge that hypothesis requires functional validation, and prospective sample collection, which is outside the scope of this work.

	fibroblasts, regardless of WHO grade or genetic background.	
4.3	In 6c, the data is comforting in that the more aggressive tumors are more similar to the earlier fibroblasts. But what are the statistics here? Are there enough independent tumors of these grades to know this for sure (seems unlikely). And what are the genes that are driving this?	We have removed the meningioma analysis, as per the recommendations of the reviewers and the editorial team.<br><br>We agree strongly with the reviewer, a greater number of meningioma samples, in a more comprehensive tumour atlas is needed to substantiate this analysis in future work.
4.4	In 6d, there are a series of genes presented that connect WHO1, 2, and 3 with fibroblast developmental stages, but less than 10. Is that significant given the number of tumors of each grade and genetic background. I suspect that significantly more tumors would need to be analyzed by single cell analysis and included in this analysis to be able to make claims about these (less than 10) genes.	We have removed the meningioma analysis, as per the recommendations of the reviewers and the editorial team.<br><br>As per our response above, we agree with the reviewer, that a significant piece of atlasing work of meningioma, across WHO grades, is needed to allow more robust analysis to be undertaken.

4.5	And the expression of these genes and the ones in 6g that are claimed to be “meningioma genes” (whatever that means) in published bulk RNA seq of large numbers of meningiomas (not referenced in this paper), is not what one would expect. Although some of these genes such as Max, and Robo1 are regulated across the population, the trend is not always in the direction that makes sense. In most cases the expression levels do not correlate with WHO grade or genetic background (which is seen for NF2 and to some degree SMARCB1). Which seems to go against the general claims of this part of the paper.	We have removed the meningioma analysis, as per the recommendations of the reviewers and the editorial team.<br><br>These were based on frequency of mutations within genes, reported in WGS studies of meningioma, curated on cbioportal.<br><br>We agree this analysis, if kept, would need to be further explored and in greater detail.

4.6	To be clear, the idea of connecting meningeal development to meningioma formation is very compelling. I just don't think that this data does that. And if I am wrong, there needs to be much more clarity in the story for this section, that could well be its own paper with proper analysis.	We agree this is a compelling line of enquiry, possibly through an independent study.

REFERENCES

Agrawal, Ankit, Stefan Thomann, Sukanya Basu, and Dominic Grün. 2024. “NiCo Identifies Extrinsic Drivers of Cell State Modulation by Niche Covariation Analysis.” *Nature Communications* 15 (1): 10628.

Birk, Sebastian, Irene Bonafonte-Pardàs, Adib Miraki Feriz, et al. 2025. “Quantitative Characterization of Cell Niches in Spatially Resolved Omics Data.” *Nature Genetics* 57 (4): 897–909.

Domínguez Conde, C., C. Xu, L. B. Jarvis, et al. 2022. “Cross-Tissue Immune Cell Analysis Reveals Tissue-Specific Features in Humans.” *Science (New York, N.Y.)* 376 (6594): eabl5197.

Kang, Minji, Gunsagar S. Gulati, Erin L. Brown, et al. 2025. “Improved Reconstruction of Single-Cell Developmental Potential with CytoTRACE 2.” *Nature Methods* 22 (11): 2258–2263.

Stickels, Robert R., Evan Murray, Pawan Kumar, et al. 2021. “Highly Sensitive Spatial Transcriptomics at near-Cellular Resolution with Slide-seqV2.” *Nature Biotechnology* 39 (3): 313–319.

Vinsland, E., S. M. Salas, I. Kapustová, et al. 2025. “Cell Atlas of the Developing Human Meninges Reveals a Dura Origin of Meningioma.” In *bioRxiv : The Preprint Server for Biology*. July 13. https://doi.org/10.1101/2025.07.08.663122.

Xu, C., M. Prete, S. Webb, et al. 2023. “Automatic Cell-Type Harmonization and Integration across Human Cell Atlas Datasets.” *Cell* 186 (26). https://doi.org/10.1016/j.cell.2023.11.026.

We thank the reviewers for their time, expertise, and detailed review of our manuscript. We believe their suggestions have significantly improved our work. We present our response to their comments, and the changes we have completed below. The format of our reply is in order of the comments outlined by the reviewers.

Changes to our manuscript that include new text, and new figures, are shown in blue text throughout the response below.

Response to Reviewer 1

	**COMMENT**	**RESPONSE**
1.1	The revised manuscript has significantly strengthened the work. The new details resample metadata, additional details of new computational pipelines are important as are the text changes. The removal of Figure 6/cancer data is also appropriate. I have no further concerns	We thank reviewer 1 for their time in reviewing our manuscript, their input has significantly improved our work.

Response to Reviewer 2 (Co-reviewed with reviewer 3)

	COMMENT	RESPONSE
1.1	We have gone through authors' reply. They have done a great job to address most of the concerns, though some are beyond the capability to fully resolve, but reasonable in our opinion. Here are a few technical comments, mainly to improve the reproducibility of the work.	We thank the reviewers for their time and comments.
1.2	The description of the nichefinder framework would benefit from a more formal presentation of the underlying mathematical steps/derivations. While the authors discuss the general pipeline (k-NN graph construction, projection to a cell type interaction matrix, normalization, and NMF-based clustering), the underlying mathematical steps/derivations connecting these steps are not clearly described.<br><br>In particular, the authors should explicitly detail:<br><br>(i) how the cell-cell adjacency matrix is constructed from spatial coordinates (including whether it is binary, weighted, heat kernel, etc),	We thank the reviewer for this suggestion and now present the mathematical framework more formally, which adds clarity to the method's description. In particular, we have addressed the raised individual sub-points as follows:<br><br>(i) how the cell-cell adjacency matrix is constructed from spatial coordinates (including whether it is binary, weighted, heat kernel, etc),<br><br>We now clarify, within the methods section, that the input spatial knn-graph can be computed with any method of choice, but that we use scanpy for that purpose in this manuscript:<br><br>"For spatial knn-aggregation nichefinder also requires a precomputed spatial k-NN graph in the AnnData.obsp['connectivities'] slot, which can be easily achieved using scanpy (sc.pp.neighbors) or squidpy (sq.gr.spatial_neighbors). Here we use default scanpy settings on 2D coordinates with k=5 to capture proximal interactions. While this uses a UMAP-style connectivity kernel[1], producing a sparse weighted connectivity matrix C, nichefinder is agnostic to the weighting scheme as long as $C \geq 0$. "<br><br>(ii) how this adjacency matrix is transformed into a cell type-cell type interaction matrix using the cell type probability matrix. Please show the mathematical operations performed to obtain

<table>
<tr>
<td></td>
<td>(ii) how this adjacency matrix is transformed into a cell type-cell type interaction matrix using the cell type probability matrix. Please show the mathematical operations performed to obtain the interaction matrix.
how the final interaction matrix under the randomized background model is computed and used for normalization, and

(iii) how the non-negative matrix factorization leads to a soft clustering of cell types into niches.

(iv) While the authors state that NMF provides a soft clustering of cell types, it is not sufficiently explained how the resulting weight matrix translates into niches.

Providing these derivations in the manuscript would greatly improve clarity and interpretability.</td>
<td>the interaction matrix. how the final interaction matrix under the randomized background model is computed and used for normalization

We now include formulas in the methods section to describe how the knn-graph and cell-type probability-matrix are transformed into a cell type interaction matrix, and how normalisation is performed:

“The cell type interaction network is then obtained by a single bilinear projection:

$$A = P^T C P,$$
where $A \in \mathbb{R}^{t \times t}$, $A \geq 0$ is the resulting cell type interaction matrix, $C \in \mathbb{R}^{n \times n}$, $C \geq 0$ the spatial connectivity matrix and $P \in \mathbb{R}^{n \times t}$, $P \in [0, 1]$ the cell-type probability matrix for n cells, t cell types and k niches. When using probabilistic cell type assignments in P, cells with fractional type membership contribute proportionally to every pairwise interaction term. Note that fixed cell type assignments can be easily used, too, by setting the probability of the respective assignments to 1.

To remove trivial enrichment arising from cell-type abundance differences, nichefinder normalises A by a randomized background model A^{rand}. In addition, we introduce a parameter $\delta \in [0, 1]$ to reduce the weight of self-interactions, which may dominate in slides with spatially segregated cell types, and focus on more informative cross-cell-type interactions. The default of $\delta = 0$ corresponds to no reduction of same-cell-type interactions, 1 to complete removal and values in between to partial reduction. With this, the random background normalisation is calculated as follows:

Step 1, estimate marginal frequencies $\hat{p}_b \in \mathbb{R}^t$ from a diagonal-deflated copy $\tilde{A} \in \mathbb{R}^{t \times t}$:</td>
</tr>
</table>

		$$\tilde{A} = A - \delta \cdot diag(A), \quad \hat{p}_b = \frac{\sum_a \tilde{A}_{ab}}{\sum_{a,b} \tilde{A}_{ab}}$$ Step 2, form the random background matrix $A^{rand} \in \mathbb{R}^{t \times t}$, calculating non-diagonal and diagonal elements as: $$A^{rand}_{ab} = \hat{p}_a \cdot \sum_c A_{cb}$$ for $a \neq b$ and otherwise $$A^{rand}_{aa} = \delta \cdot A_{aa} + (1 - \delta) \cdot \hat{p}_a \cdot \sum_c A_{ca}.$$ Step 3, element-wise normalisation into a matrix $\hat{A} \in \mathbb{R}^{t \times t}$: $$\hat{A}_{ab} = \frac{A_{ab}}{A^{rand}_{ab}}$$ After that, values $\hat{A}_{ab} > 1$ indicate co-localisation enrichment and values < 1 indicate depletion relative to the spatial random baseline. Thus, $\hat{A}$ constitutes a cell type-to-cell type adjacency matrix that quantifies the spatial association strength between pairs of cell types.” <br>(iii) how the non-negative matrix factorization leads to a soft clustering of cell types into niches. <br>We now explain more clearly how NMF is applied and how the soft-clustering matrix is obtained as follows (for a justification of cell type clustering and niche-interpretation see point iv):

		“Finally, for niche clustering (3), the normalized cell-type by cell-type matrix $\hat{A} \geq 0$ is factorised using non-negative matrix factorization (NMF) as: $$\hat{A} \approx W H,\ W \in \mathbb{R}^{t\times k} \geq 0, H \in \mathbb{R}^{k\times t} \geq 0,$$ using the NMF implementation in sklearn[2], which minimises the Frobenius reconstruction error $min_{W,H \geq 0} \lVert\hat{A} - WH\rVert_F^2$. If desired, the optimal rank k can be selected as the elbow of the reconstruction-error curve $\left\{ \lVert\hat{A} - W_k H_k\rVert_F \right\}_{k=1}^{k_{max}}$ via the second discrete-derivative method. In the case of a directed, asymmetric cell-type adjacency matrix $\hat{A}$, the resulting matrices W and H could be interpreted as sender and receiver profiles of niches. However, for the standard knn-based co-localisation analysis performed here, W and H are blended into a single niche-membership matrix $S \in \mathbb{R}^{t\times k}$: $$S = \alpha \cdot col\_norm(W) + (1 - \alpha) \cdot col\_norm(H^T),$$ where $\alpha = 0.5$. This is justified if $col\_norm(W) \approx col\_norm(H^T)$, which is usually the case for symmetric $\hat{A}$, and can be assessed via the mean column-wise cosine similarity between col-normalised W and H^T; values close to 1 (>0.95) indicate the two factors encode equivalent niche profiles. As an alternative, we implemented a symmetric NMF variant that solves $\hat{A} \approx H H^T$ via a multiplicative update with column renormalisation in each step to prevent column collapse to zero[3]: $H \leftarrow H \odot \frac{\hat{A} H}{H H^T H + \varepsilon}$, producing a single niche-membership matrix H.”<br><br>(iv) While the authors state that NMF provides a soft clustering of cell types, it is not sufficiently explained how the resulting weight matrix translates into niches.

		We now provide some justification why the NMF components correspond to clusters and may be interpreted as niches, while also putting this interpretation into perspective:<br><br>"The weights of the k factors in $S \in \mathbb{R}^{t \times k}$ over t cell-types can be interpreted as membership weights of cell-types in clusters, or "topics" (motifs) of frequently co-localising cell types. Similar to the application of NMF in document text analysis and other tasks[4,5], cell types fall into clusters, because since W and H are non-negative, the columns of S are additive and cannot cancel each other, forcing each NMF component to explain a distinct, non-overlapping portion of the observed co-localisation signal—while optimising the low-rank approximation of the original cell-type interaction matrix incentivises the capture of frequent motifs[6]. Thus, nichefinder yields a soft-clustering (the matrix S) of cell types into co-localisation motifs, where each cell type can be part of multiple motifs, allowing to capture nuanced participation patterns (e.g. the same immune cell type in different microenvironments), not forcing cell types to "pick" a cluster. Such global cell-type co-localization motifs can be interpreted as cell-type niches depending on which aspects to highlight in the definition of a niche: nichefinder motifs are likely to match microenvironments that are characterized by cell type composition and re-occur across space. Thus, nichefinder focuses on distinguishing global archetypical interaction patterns from noise and background distributions. On the other hand, rare structurally characterised microenvironments (e.g. a small diseased area) or microenvironments characterised by molecule concentrations (such as secreted ligands) may be better identified with other respectively specialised methods."
1.3	Again, the description of how NMF leads to niche identification requires further clarification. By definition, NMF decomposes a non-negative matrix X into two non-negative matrices W and H, such that X≃WH.	Please see our response to point 1.2.<br><br>In particular, we now clarify the handling of the factorization output (W and H):<br><br>"In the case of a directed, asymmetric cell-type adjacency matrix $\hat{A}$, the resulting matrices W and H could be interpreted as sender and receiver profiles of niches. However, for the standard knn-based co-localisation analysis performed here,

	However, this decomposition does not inherently perform clustering of the original data. It is therefore unclear which of the decomposed matrices (W or H) is interpreted as the cell type-by-niche matrix, and how this interpretation is justified.<br><br>The authors should explicitly state how soft clustering is derived from this matrix.	W and H are blended into a single niche-membership matrix $S \in \mathbb{R}^{t \times k}$:<br><br>$S = \alpha \cdot col\_norm(W) + (1 - \alpha) \cdot col\_norm(H^{T})$,<br><br>where $\alpha = 0.5$. This is justified if $col\_norm(W) \approx col\_norm(H^{T})$, which is usually the case for symmetric $\hat{A}$, and can be assessed via the mean column-wise cosine similarity between col-normalised W and H^{T}; values close to 1 (>0.95) indicate the two factors encode equivalent niche profiles. As an alternative, we implemented a symmetric NMF variant that solves $\hat{A} \approx H H^{T}$ via a multiplicative update with column renormalisation in each step to prevent column collapse to zero[3]: $H \leftarrow H \odot \frac{\hat{A} H}{H H^{T} H + \varepsilon}$, producing a single niche-membership matrix H."<br><br>And mention the inherent clustering property of NMF, which has been described in more detail in the literature:<br><br>"Similar to the application of NMF in document text analysis and other tasks[4,5], cell types fall into clusters, because since W and H are non-negative, the columns of S are additive and cannot cancel each other, forcing each NMF component to explain a distinct, non-overlapping portion of the observed co-localisation signal—while optimising the low-rank approximation of the original cell-type interaction matrix incentivises the capture of frequent motifs[6]. Thus, nichefinder yields a soft-clustering (the matrix S) of cell types into co-localisation motifs, where each cell type can be part of multiple motifs, allowing to capture nuanced participation patterns (e.g. the same immune cell type in different microenvironments), not forcing cell types to "pick" a cluster."
1.4	More importantly, the manuscript should clarify how a low-rank factorization of a global interaction matrix translates into niches, rather than abstract latent components.	Please see our responses to points 1.2 and 1.3.<br><br>In addition to describing the clustering property of NMF, we now also justify the interpretation as niches in put it into context in the methods section:

		"Such global cell-type co-localization motifs can be interpreted as cell-type niches depending on which aspects to highlight in the definition of a niche: nichefinder motifs are likely to match microenvironments that are characterized by cell type composition and re-occur across space. Thus, nichefinder focuses on distinguishing global archetypical interaction patterns from noise and background distributions. On the other hand, rare structurally characterised microenvironments (e.g. a small diseased area) or microenvironments characterised by molecule concentrations (such as secreted ligands) may be better identified with other respectively specialised methods."
1.5	The terminology used to describe the nichefinder method and its output requires reconsideration. Referring to the algorithm as "nichefinder" and its results as "niches" is potentially misleading, as the inferred patterns appear to reflect global cell type co-localization or interaction profiles rather than spatially localized microenvironments. In the current form, the results seem more consistent with cell-type interaction states than spatial niches. The authors should revisit this interpretation and either provide stronger evidence that the inferred factors correspond to spatially localized niches (e.g., through direct spatial visualization) or adopt more precise terminology that better reflects the nature of the results.	Indeed, as the reviewer notes, the soft-clusters identified by nichefinder are solely based on spatial co-localisation patterns of cell types (gene expression input is incorporated indirectly via the annotation of cell types or states). Therefore, nichefinder clusters may be described as cell-type interaction motifs, spatially localised cell-type patterns that re-occur across a slide (these may not be localised to only one region, but are localised nonetheless). Depending on the purpose of a given spatial analysis, the most useful definition of "niche" may differ. We reason that the local composition of cell states, which nichefinder assigns into clusters, is one of such useful definitions of a niche. For example, it may identify that the same immune cell type occurs in two different archetypical contexts on a slide linked with distinct groups of other cell types.<br><br>We now describe this reasoning more clearly within the methods section and also compare the nichefinder output to niche-definitions with a different focus (also see point 1.4):<br><br>"Such global cell-type co-localization motifs can be interpreted as cell-type niches depending on which aspects to highlight in the definition of a niche: nichefinder motifs are likely to match microenvironments that are characterized by cell type composition and re-occur across space. Thus, nichefinder focuses on distinguishing global archetypical interaction patterns from noise and background distributions. On the other hand, rare structurally characterised microenvironments (e.g. a small diseased area) or microenvironments characterised by

		molecule concentrations (such as secreted ligands) may be better identified with other respectively specialised methods."
1.6	The authors introduce Cell2home as a tool for cell-cell interaction analysis. However, its performance is not validated. Given that this is a method focused contribution included within a resource paper, it is important to demonstrate that the proposed Cell2home provides clear added value or, at minimum, performs comparably to established cell-cell interaction analysis frameworks e.g. by comparing overlaps or differences in identified interactions.	We thank the editor for their comment: "*Regarding the point about how Cell2home compares to established cell-cell interaction analysis frameworks, we would welcome such data if they exist, but we would be happy to also accept addressing this point only textually.*"<br><br>The reviewer brings up an important point regarding distinguishing cell2home from other cell-cell interaction analysis algorithms. The key advance of cell2home is to enable data-driven prediction of cell homing to other cells, something that no other tool to our knowledge does. This lack of functionality in existing tools motivated us to develop the algorithm.<br><br>Crucially, the key feature of aggregating all migration cues received by a cell from another cell, a central feature of cell2home, is not provided by any other tool. While tools like CellPhoneDB and CellChat enable in silico screens to identify all predicted ligand-receptor interactions between two cell types, they do not provide an estimate of which of these interactions influence cell homing and importantly do not provide an aggregated score enabling ranking cell types based on homing affinity. Thus, the key unique functionality of cell2home is the homing affinity between cell types, not identification of individual ligand-receptor interactions. However, as an additional feature, cell2home allows dissecting the contributions of individual ligand-receptor pairs to the aggregated affinity score.<br><br>Based on the unique functionality of cell2home, we consider it appropriate to focus on the added value as suggested by the reviewer and clarify the specific use cases of cell2home in the manuscript text, instead of attempting benchmarking between tools that perform different functions.

		We have now clarified these points in the discussion as follows:<br><br>“We developed cell2home to provide insights into homing interactions establishing B cell localisation in vascular and fibroblast niches in the developing meninges. This computational approach is based on the key biologically-grounded insight that the functional output of chemoattractant receptor signalling in response to ligand engagement, across all of the different receptors, is to increase migration of the receptor-expressing cell towards increasing density of the ligand. Therefore these ligand-receptor interactions with identical outputs can be aggregated together into a single homing affinity score between two cell types. The homing affinity score can then be used to identify which cell type a given cell is predicted to home towards. While tools like CellPhoneDB and CellChat answer the question of which ligand-receptor interactions are predicted to occur between two cell types, cell2home answers the question of which cell types are predicted to attract a given cell to home towards them. We envision cell2home to have utility in deciphering how immune cells are recruited to tissue niches during development across organs as well as in inflammation and cancer.”
1.7	Lastly, please ensure that all previously published algorithms and methods used in this study are appropriately cited in the relevant sections.	We have now included citations that are main-text only, and methods only, as recommended by the editor, to align with journal requirements and to improve clarity.

References

1. Wolf, F. A., Angerer, P. & Theis, F. J. SCANPY: large-scale single-cell gene expression data analysis. *Genome Biol* **19**, 15 (2018).
2. Pedregosa, F. *et al.* Scikit-learn: Machine Learning in Python. *Journal of Machine Learning Research* 2825–2830 (2011).
3. Kuang, D., Ding, C. & Park, H. Symmetric Nonnegative Matrix Factorization for Graph Clustering. in *Proceedings of the 2012 SIAM International Conference on Data Mining* 106–117 (2012).
4. Brunet, J.-P., Tamayo, P., Golub, T. R. & Mesirov, J. P. Metagenes and molecular pattern discovery using matrix factorization. *Proc Natl Acad Sci U S A* **101**, 4164–4169 (2004).
5. Xu, W., Liu, X. & Gong, Y. Document clustering based on non-negative matrix factorization. in *Proceedings of the 26th annual international ACM SIGIR conference on Research and development in informaion retrieval* 267–273.
6. Simon, H. D., Ding, C., He, X. & Jin, R. *On the Equivalence of Nonnegative Matrix Factorization and K-Means- Spectral Clustering*. (2005).
